# Synthesis, Structure–Activity Relationships, and Antiviral Profiling of 1-Heteroaryl-2-Alkoxyphenyl Analogs as Inhibitors of SARS-CoV-2 Replication

**DOI:** 10.3390/molecules27031052

**Published:** 2022-02-04

**Authors:** Dorothée Bardiot, Laura Vangeel, Mohamed Koukni, Philippe Arzel, Marleen Zwaagstra, Heyrhyoung Lyoo, Patrick Wanningen, Shamshad Ahmad, Linlin Zhang, Xinyuanyuan Sun, Adrien Delpal, Cecilia Eydoux, Jean-Claude Guillemot, Eveline Lescrinier, Hugo Klaassen, Pieter Leyssen, Dirk Jochmans, Karolien Castermans, Rolf Hilgenfeld, Colin Robinson, Etienne Decroly, Bruno Canard, Eric J. Snijder, Martijn J. van Hemert, Frank van Kuppeveld, Patrick Chaltin, Johan Neyts, Steven De Jonghe, Arnaud Marchand

**Affiliations:** 1 Centre for Innovation and Stimulation of Drug Discovery (CISTIM), Gaston Geenslaan 2, 3001 Leuven, Belgium; dorothee.bardiot@cistim.be (D.B.); mohamed.koukni@cistim.be (M.K.); philippe.arzel@cistim.be (P.A.); hugo.klaassen@cistim.be (H.K.); karolien.castermans@cistim.be (K.C.); patrick.chaltin@kuleuven.be (P.C.); 2 Laboratory of Virology and Chemotherapy, KU Leuven, Department of Microbiology, Immunology and Transplantation, Rega Institute for Medical Research, Herestraat 49, 3000 Leuven, Belgium; laura.vangeel@kuleuven.be (L.V.); pieter.Leyssen@kuleuven.be (P.L.); dirk.jochmans@kuleuven.be (D.J.); johan.neyts@kuleuven.be (J.N.); 3 Virology Section, Infectious Disease and Immunology Division, Department of Biomolecular Health Sciences, Faculty of Veterinary Medicine, Utrecht University, 3584 CL Utrecht, The Netherlands; m.zwaagstra@uu.nl (M.Z.); h.r.lyoo@uu.nl (H.L.); f.j.m.vankuppeveld@uu.nl (F.v.K.); 4 Department of Medical Microbiology, Leiden University Medical Center, 2300 RC Leiden, The Netherlands; p.wanningen@lumc.nl (P.W.); e.j.snijder@lumc.nl (E.J.S.); m.j.van_hemert@lumc.nl (M.J.v.H.); 5 Drug Discovery Unit, School of Life Sciences, University of Dundee, Dundee DDI 5EH, UK; s.a.ahmad@dundee.ac.uk (S.A.); c.x.robinson@dundee.ac.uk (C.R.); 6 Institute of Molecular Medicine, University of Lübeck, 23562 Lübeck, Germany; llzhang@biochem.uni-luebeck.de (L.Z.); xinyuanyuan.sun@uni-luebeck.de (X.S.); rolf.hilgenfeld@uni-luebeck.de (R.H.); 7 Laboratory Architecture et Fonction des Macromolécules Biologiques (AFMB), UMR 7257, Centre National de la Recherche Scientifique (CNRS), Aix Marseille University, CEDEX 9, 13288 Marseille, France; adrien.delpal@univ-amu.fr (A.D.); cecilia.eydoux@univ-amu.fr (C.E.); jean-claude.guillemot@univ-amu.fr (J.-C.G.); etienne.decroly@univ-amu.fr (E.D.); bruno.canard@univ-amu.fr (B.C.); 8 Medicinal Chemistry, Rega Institute for Medical Research, KU Leuven, Herestraat 49, 3000 Leuven, Belgium; eveline.lescrinier@kuleuven.be; 9 German Center for Infection Research (DZIF), Hamburg-Lübeck-Borstel-Riems Site, University of Lübeck, 23562 Lübeck, Germany; 10 Center for Drug Design and Development (CD3), KU Leuven R&D, Waaistraat 6, 3000 Leuven, Belgium

**Keywords:** COVID-19, SARS-CoV-2, 1,2,4-oxadiazole, 1-heteroaryl-2-alkoxyphenyl analogs

## Abstract

The severe acute respiratory syndrome coronavirus 2 (SARS-CoV-2), the causative agent of COVID-19, has led to a pandemic, that continues to be a huge public health burden. Despite the availability of vaccines, there is still a need for small-molecule antiviral drugs. In an effort to identify novel and drug-like hit matter that can be used for subsequent hit-to-lead optimization campaigns, we conducted a high-throughput screening of a 160 K compound library against SARS-CoV-2, yielding a 1-heteroaryl-2-alkoxyphenyl analog as a promising hit. Antiviral profiling revealed this compound was active against various beta-coronaviruses and preliminary mode-of-action experiments demonstrated that it interfered with viral entry. A systematic structure–activity relationship (SAR) study demonstrated that a 3- or 4-pyridyl moiety on the oxadiazole moiety is optimal, whereas the oxadiazole can be replaced by various other heteroaromatic cycles. In addition, the alkoxy group tolerates some structural diversity.

## 1. Introduction

The severe acute respiratory syndrome coronavirus 2 (SARS-CoV-2) is the causative agent of the human coronavirus disease 2019 (COVID-19). SARS-CoV-2 is a newly discovered coronavirus that was first identified in December 2019 in Wuhan, China, and spread quickly throughout the world, infecting and causing death to millions of people [1,2]. On 11 March 2020, the World Health Organization (WHO) officially declared COVID-19 a global pandemic. As of October 2021, more than 235 million people were infected with SARS-CoV-2 and almost 5 million people have died due to COVID-19 [3].

Major research efforts were carried out to control this pandemic. Using a number of different technology platforms (such as messenger RNA and vector-based vaccines), the development of various vaccines moved forward at an unparalleled speed and several of them received marketing approval [4]. Despite the success of these SARS-CoV-2 vaccines, there is still an urgent need for small molecule therapeutics. As a drug discovery program aiming at the development of SARS-CoV-2-specific antiviral drugs from scratch would take a long time, the initial focus has been on the repurposing of known drugs and drug candidates. Several large-scale phenotypic screening campaigns of different repurposing compound libraries have been described in the literature. With different SARS-CoV-2-infected cell lines and different readouts being used, the outcome has been heterogeneous and a wide variety of compounds has been discovered [5,6,7,8].

Drug discovery programs focusing on viral proteins that are essential for SARS-CoV-2 replication are also being pursued. The viral genome of SARS-CoV-2 encodes for 16 non-structural proteins (nsp1–nsp16). These are highly conserved among various coronaviruses and many of them are excellent candidates as targets for the discovery of antiviral agents [9]. Examples include the cap guanine N7-methyltransferase and 3′–5′ exonuclease activity of nsp14 [10], the nsp15 endoribonuclease [11], and the papain-like protease (PLpro) activity of nsp3 [12]. Targets that are most intensively studied are nsp5, also known as the chymotrypsin-like protease (3CLpro) or the viral main protease (Mpro) [13] and the RNA-dependent RNA polymerase (RdRp, encoded by nsp12) [14,15]. Drug discovery programs on the latter two enzymes have afforded drugs and drug candidates for the treatment of SARS-CoV-2 viral infections. PF-00835231 is a potent SARS-CoV-2 Mpro inhibitor, when evaluated in a biochemical assay and shows potent SARS-CoV-2 antiviral activity in a cell-based antiviral assay. Its corresponding phosphate prodrug, also known as PF-07304814, is currently undergoing clinical trials for COVID-19 treatment, after intravenous administration [16]. More recently, Pfizer disclosed PF-07321332, an orally bioavailable SARS-CoV-2 Mpro inhibitor, displaying excellent in vitro potency in biochemical and cell-based antiviral assays. Moreover, it is endowed with good activity in SARS-CoV-2 mouse and hamster models, after oral administration [17]. Pfizer recently reported interim data of a phase II/III clinical trial (unpublished data) demonstrating a marked reduction in progression to severe COVID-19 or hospitalization if treatment in high-risk patients is initiated during the first 5 days of onset of symptoms.

The viral RNA-dependent RNA polymerase (RdRp) is another attractive target for the treatment of COVID-19 patients. Remdesivir is a phosphoramidate prodrug of the *C*-nucleoside GS-441524 that received FDA approval for the treatment of adult and pediatric (aged > 12 years) SARS-CoV-2-infected patients. Since it requires intravenous administration, remdesivir usage is limited to hospital settings [18]. Yet, Gilead reported (unpublished) that early intravenous treatment of at risk-patients markedly reduced progression to severe COVID-19. Molnupiravir, an ester prodrug of *N*-4-hydroxycytidine, is orally bioavailable and has shown promising activity in various SARS-CoV-2 preclinical animal models [19] and was licensed for medical use in the United Kingdom and the EU for use in patients suffering from mild to moderate COVID-19 and who have at least one risk factor for developing severe illness.

Cell-based phenotypic based screening campaigns of structurally diverse, drug-like compound libraries are another important method to discover novel antiviral hit compounds which serve as a basis for further development. Advantages of this approach are that such screens are physiologically more relevant than target-based screens and that there is a priori no bias towards a particular target and hence allows to select antiviral agents targeting different viral, as well as cellular, factors [20]. To the best of our knowledge, this type of screening for the discovery of novel hit matter against SARS-CoV-2 has not been described in the literature. In this manuscript, the discovery of a novel SARS-CoV-2 hit compound, its preliminary structure–activity relationship (SAR) study, and its antiviral profile are discussed.

## 2. Results

### 2.1. Hit Identification

**Figure 1:**
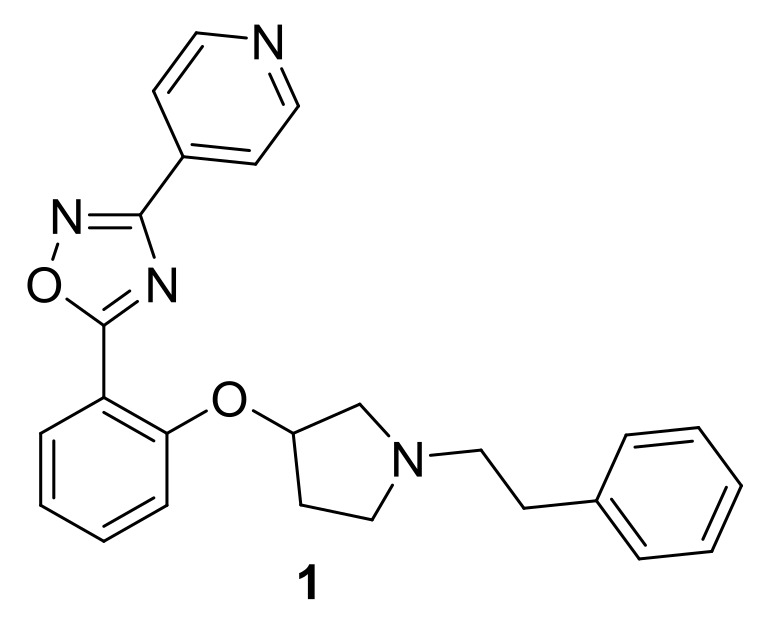
Structure of hit compound **1**.

In order to identify potential hits, the CD3 (Centre for Drug Design and Discovery, KU Leuven) small molecule library was screened against SARS-CoV-2 in VeroE6 cells, which constitutively expresses the enhanced green fluorescent protein (eGFP) [21]. A reduced eGFP expression correlates with the cytopathogenic effect (CPE) of SARS-CoV-2 and, hence, the measurement of the fluorescence by high-content imaging can be used as an indication for the antiviral potency. In the presence of a non-toxic antivirally active agent, the cytopathogenicity is inhibited and the fluorescent signal is maintained. In the first round of screening, compounds were tested at a single concentration of 10 µM in a 384-well plate format. Initial hits were followed up by full-dose response analysis allowing to express the antiviral activity as the concentration producing 50% antiviral effect (EC_50_). In parallel, the cellular toxicity of the compounds, which was expressed as the 50% cytotoxic concentration (CC_50_), was determined using mock-infected VeroE6 cells. Among the various hits, 5-(2-((1-phenethylpyrrolidin-3-yl)oxy)phenyl)-3-(pyridin-4-yl)-1,2,4-oxadiazole **1** (Figure 1) displayed an EC_50_ value of 4.7 µM and a CC_50_ value of 21 µM, and was considered a promising starting point for further chemistry.

### 2.2. Chemistry

**Scheme 1:**
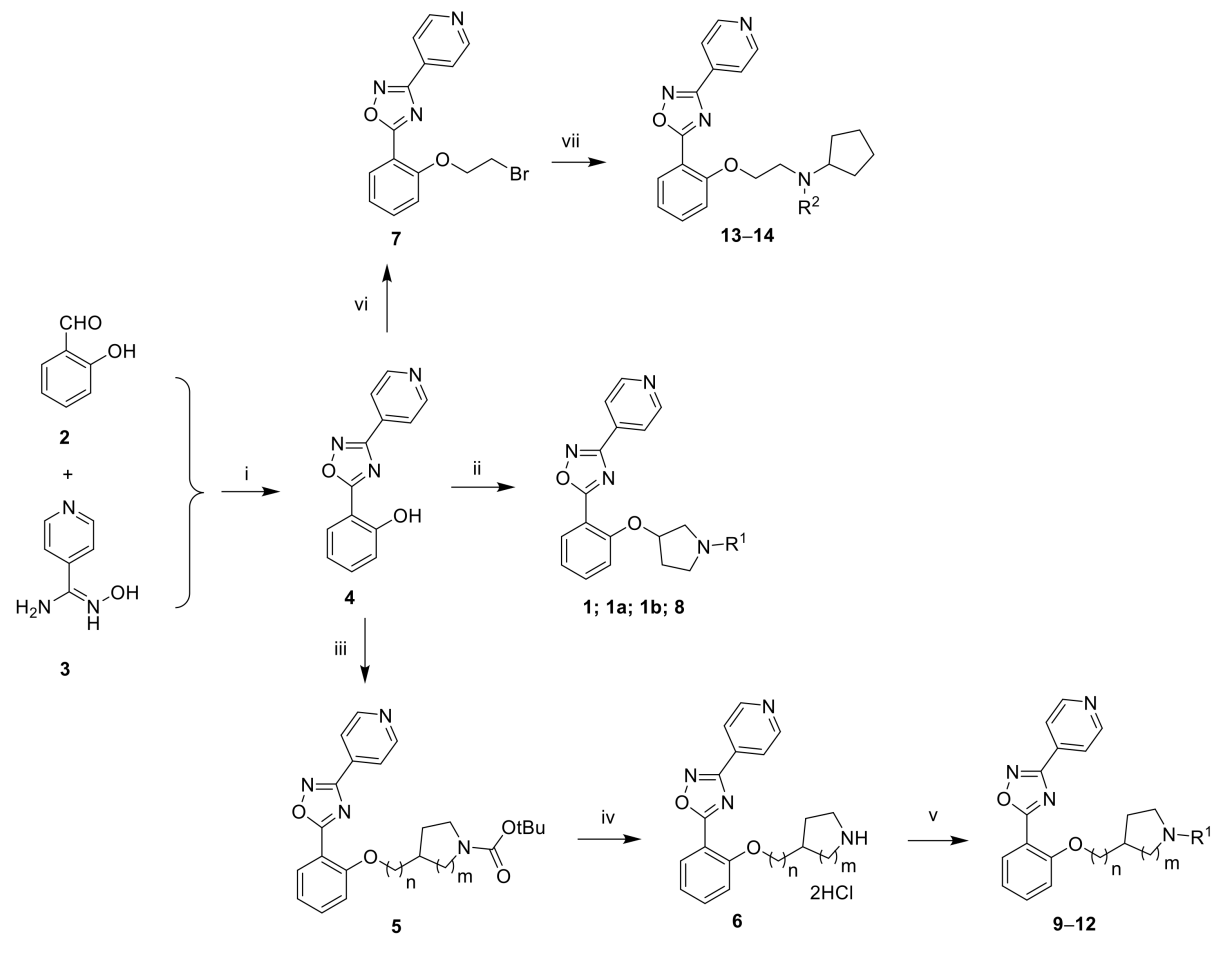
Synthetic routes to compound **1** and analogs with modification of the alkoxy substituent. Reagents and conditions: (i) EtOH, MW irradiation, 160 °C, 1 h, 26%; (ii) nBu_3_P, ADDP, alcohol, THF, rt, 2 d, 51%; (iii) PPh_3_, DIAD, alcohol, THF, rt, overnight, 95–99%; (iv) 4N HCl in dioxane, dioxane, rt, 4 to 16 h, 100%; (v) R^1^-X, K_2_CO_3_, DMF, 80 °C, 2 h, 26–40%; (vi) dibromoethane, K_2_CO_3_, DMF, 80 °C, 2 h, 46%; (vii) amine, K_2_CO_3_, DMF, 80 °C, 2 h, 20%.

Compound **1** and analogs with various alkoxy substituents on the phenyl ring were prepared following one of the three synthetic routes as shown in Scheme 1. In a first step, the key intermediate 1,2,4-oxadiazole **4** was synthesized by condensation under microwave irradiation [22] of the benzaldehyde **2** and the N’-hydroxy-isonicotinamidine **3**. Several methods were used to introduce the alkoxy substituents. Mitsunobu reactions with the corresponding 3-hydroxypyrrolidine analog using 1,1′-(azodicarbonyl)dipiperidine (ADDP) [23] and tributylphosphine provided the desired compounds **1** and **8** in one step. To introduce the desired substituents (R^1^ group) on the cyclic amine at the final step, an alternative three-step sequence was developed. A Mitsunobu reaction with commercially available alcohols bearing a Boc-protected cyclic amine afforded intermediates **5**, which were deprotected in acidic conditions to release the free amines **6**, which were subsequently alkylated with various alkyl or cycloalkyl halides, affording target compounds **9**–**12**. Alternatively, alkylation of **4** with dibromoethane, followed by bromine displacement with cyclopentylamines, allowed to prepare compounds **13** and **14** with an ethyleneoxy linker between the phenyl and the amine moiety.

**Scheme 2:**
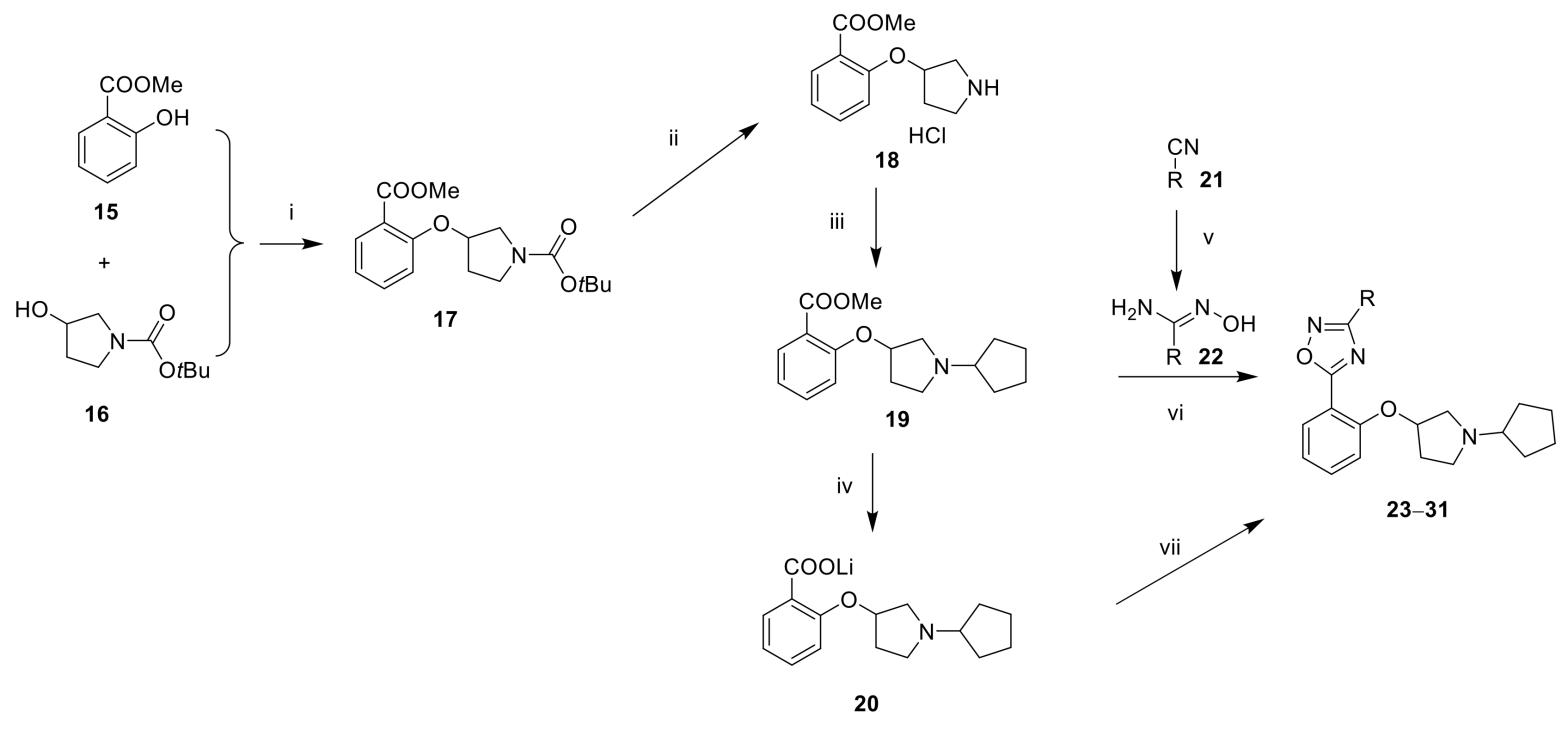
Synthetic route to compounds with various substituents on the oxadiazole. Reagents and conditions: (i) PPh_3_, DIAD, THF, rt, overnight, 33%; (ii) 4N HCl in dioxane, dioxane, rt, overnight, 100%; (iii) cyclopentanone, NaBH(OAc)_3_, CH_2_Cl_2_, rt, overnight, 77%; (iv) LiOH, THF, MeOH, H_2_O, 60 °C, 6 h, 82%; (v) NH_2_OH.HCl, K_2_CO_3_, EtOH, 80 °C, overnight; (vi) **22**, NaOH, DMSO, rt, overnight, 4%; (vii) (a) 22, HATU, DIPEA, DMF, rt, 6 h; (b) 100 °C, overnight, 15%.

In order to access a small library of compounds bearing various groups on the oxadiazole, a convergent route was designed to generate several compounds in one step from a common intermediate (Scheme 2). Briefly, the key building block **19** was prepared in three steps from phenol **15** and *N*-Boc-hydroxy-pyrrolidine **16** by Mitsunobu reaction, followed by Boc deprotection and *N*-alkylation by reductive amination with cyclopentanone. The ester **19** or the lithium salt of the corresponding carboxylic acid **20** were then converted into the desired oxadiazoles **23**–**31** by condensation with various amidoximes **22**, which were either commercially available or prepared by nucleophilic attack of hydroxylamine on appropriate nitriles **21.**

**Scheme 3:**
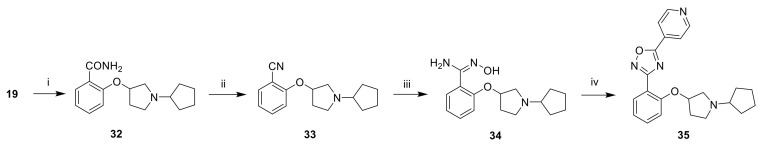
Synthetic route to compound **35** with an isomeric 1,2,4-oxadiazole ring. Reagents and conditions: (i) NH_4_OH, dioxane, 100 °C, overnight; (ii) T3P, EtOAc, 60 °C, overnight, 54% over 2 steps; (iii) NH_2_OH.HCl, DIPEA, EtOH, 70 °C, overnight, crude; (iv) (a) isonicotinic acid, EDC.HCl, HOBt, DIPEA, DMF, rt, 6 h; (b) 80 °C, overnight, 9%.

**Scheme 4:**
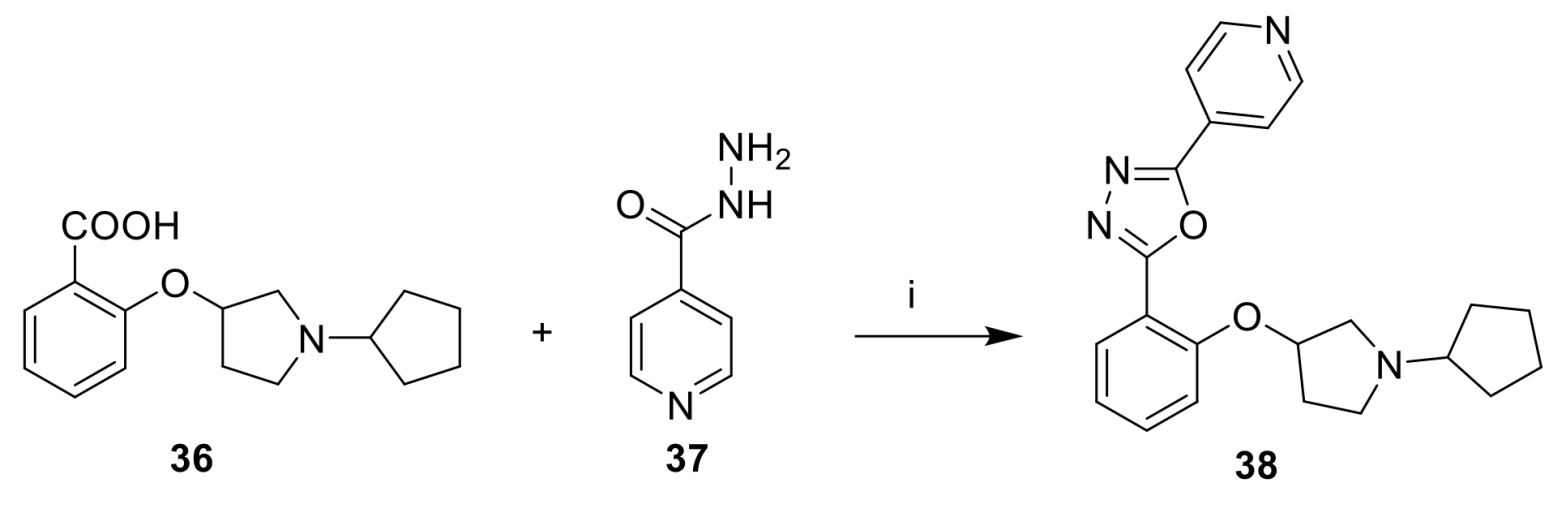
Synthetic route to compound **38** with a 1,3,4-oxadiazole ring. Reagents and conditions: (i) POCl_3_, 90 °C, overnight, 19%.

**Scheme 5:**
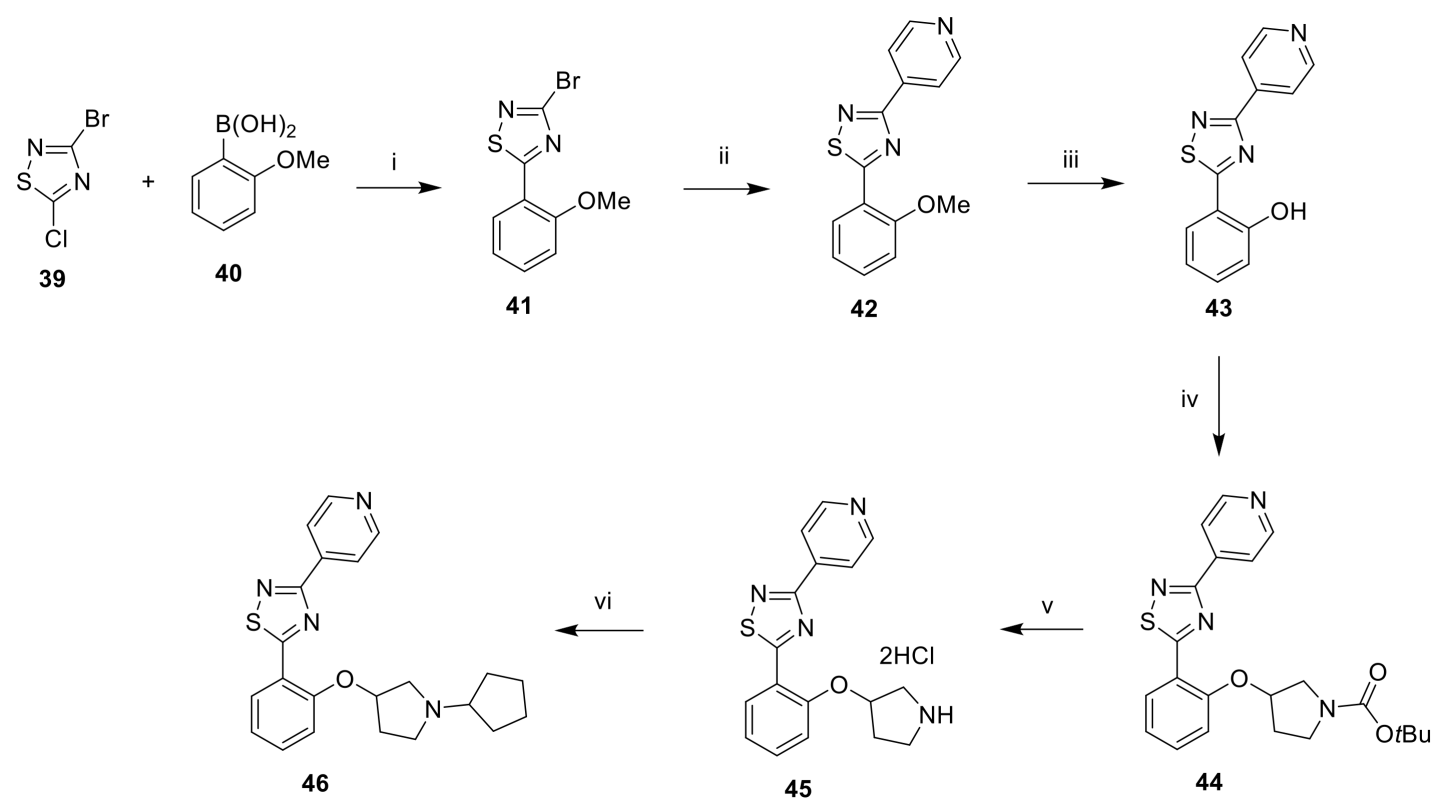
Synthetic route to compound **46** with a 1,2,4-thiadiazole ring. Reagents and conditions: (i) PdCl_2_(dppf), CsF, dioxane, H_2_O, 85 °C, overnight, 46%; (ii) pyridin-4-ylboronic acid, PdCl_2_(dppf), K_2_CO_3_, dioxane, H_2_O, 85 °C, overnight, 83%; (iii) LiBr, pTsOH, NMP, 150 °C, 1.5 h, 54%; (iv) PPh_3_, DIAD, *tert*-butyl 3-hydroxypyrrolidine-1-carboxylate, THF, rt, overnight, 35%; (v) 4N HCl in dioxane, dioxane, rt, 2 h, 100%; (vi) cyclopentanone, NaBH(OAc)_3_, MeOH, CH_2_Cl_2_, rt, overnight, 9%.

**Scheme 6:**
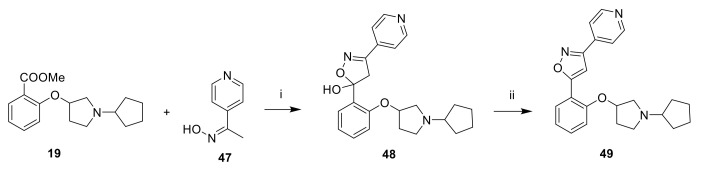
Synthetic route to compound **49** with an isoxazole ring. Reagents and conditions: (i) 2M LDA in THF, −78 °C, 2.5 h, crude; (ii) 12N HCl, MeOH, 70 °C, 3 h, 12%.

In order to study the importance of the 1,2,4-oxadiazole ring for antiviral activity, several synthetic routes were developed to obtain analogs bearing other 5-membered heteroaryls (Scheme 3, Scheme 4, Scheme 5 and Scheme 6). The synthesis of an analog having an isomeric 1,2,4-oxadiazole ring is shown in Scheme 3. The required amidoxime **34** was synthesized in three steps from intermediate **19**. Treatment of the ester with aqueous ammonia yielded the primary amide **32**. Dehydration with propylphosphonic anhydride (T3P) [24], followed by treatment with hydroxylamine yielded amidoxime **34**. Oxadiazole ring formation via an O-acylamidoxime intermediate using EDC/HOBt as coupling agents provided the desired compound **35**.

The reaction of carboxylic acid **36** with isonicotinohydrazide **37** using phosphorus oxychloride as a dehydrating agent provided compound **38** with a 1,3,4-oxadiazole ring (Scheme 4).

To access compound **46**, the 1,2,4-thiadiazole ring was not synthesized since thiadiazole **39** is a commercially available building block (Scheme 5) [25]. A first selective Suzuki–Miyaura coupling using ortho-methoxyphenylboronic acid occurred on the chlorine and was followed by a second coupling with pyridin-4-ylboronic acid furnishing intermediate **42**. Demethylation with LiBr led to the formation of the phenol **43**, which underwent a Mitsunobu reaction with *N*-Boc-3-hydroxypyrrolidine to provide intermediate **44**. Removal of the Boc protecting group under acidic conditions, followed by alkylation of the pyrrolidine moiety by reductive amination, furnished the desired compound **46**.

The isoxazole analog **49** was obtained via a cyclization of oxime **47** with ester **19** in the presence of LDA, followed by treatment with 12N HCl at 70 °C.

### 2.3. Biological Evaluation

#### 2.3.1. Early SAR Investigation

All compounds were evaluated for anti-SARS-CoV-2 activity in VeroE6 cells, which allowed to establish first SAR trends. As these cells have a high expression of the efflux transporter P-glycoprotein (P-gp), the assays were performed in the presence of CP-100356, a well-known P-gp inhibitor [12]. In all experiments, GS-441524 (the parent nucleoside of remdesivir) [26] was included as a positive control, with an average EC_50_ and CC_50_ value of 0.82 µM and 72.9 µM, respectively.

As compound **1** presents a chiral center, the two enantiomers **1a** and **1b** were synthesized using optically pure pyrrolidine building blocks and tested against SARS-CoV-2. The three compounds **1**, **1a**, and **1b** showed similar biological profiles, indicating a limited influence of the stereochemistry on the antiviral activity (Table 1). All the other compounds were then synthesized and evaluated as racemic mixtures.

The SAR was explored around three distinct structural subunits of hit compound **1**: The alkoxy group on the phenyl moiety, the substituent on the oxadiazole ring, and the oxadiazole ring itself. We first investigated the alkoxy group modifications (Table 2), taking into account that an amino group was required for antiviral activity (data not shown). Several structural modifications around this alkoxy group were well tolerated: Replacement of the phenethyl substituent on the pyrrolidine of hit compound **1** by a cyclopentyl (compound **8**) or a methylcyclopentyl (compound **9**) moiety, the insertion of an additional methylene linker between the oxygen and the pyrrolidine (compound **10**) or replacement of the pyrrolidine by a piperidine (compound **12**). A cyclic amine was not essential for the antiviral potency as shown by compounds **13** and **14** with a linear secondary or tertiary amine. On the other hand, the introduction of a methylene group between the pyrrolidine and the phenoxy ring of compound **10** yielded analog **11**, which was 20-fold less active than compound **10**.

We next investigated modifications around the 1,2,4-oxadiazole subunit (Table 3). To probe the effect of substituents on the 1,2,4-oxadiazole moiety on the antiviral activity, a number of derivatives were synthesized (compounds **23**–**31**). Albeit the 3-pyridinyl analog **23** was equipotent to **8**, the introduction of a second nitrogen into the ring led to potency loss (3-fold for the pyrimidine **24** and more than 10-fold for the pyridazine **25**). The phenyl analog **26** showed only cytotoxicity. Since the 3- or 4-pyridyl derivatives seemed to be the optimal 6-membered ring, the addition of substituents was explored. Several groups with various sizes and electronic properties were well tolerated at different positions of the 3-pyridyl moiety, such as a methoxy (compound **27**), a trifluoromethyl (compounds **28**–**29**), or a methyl group (compound **30**). The positive effect of the methyl group in ortho position to the oxadiazole was confirmed also with the 4-pyridyl analog **31**.

The importance of the 1,2,4-oxadiazole moiety for antiviral activity was investigated by the synthesis of a number of alternative 5-membered heteroaryl derivatives (Table 4). Except for compound **38** with a 1,3,4-oxadiazole moiety, which was 3-fold less active, other derivatives containing a 1,2,4-oxadiazole (**35**), a 1,2,4-thiadiazole (**46**) or an isoxazole (**49**) showed a similar biological profile as compound **8**, indicating that the 1,2,4-oxadiazole ring is not involved in crucial interactions for antiviral activity, but can be considered as a linker.

#### 2.3.2. Broad-Spectrum Antiviral Activity

The primary SARS-CoV-2 screening focused on the B.1 (Wuhan) lineage. However, several variants of SARS-CoV-2 have emerged and are circulating worldwide, and more will emerge in the future. Therefore, it is important to determine the activity of antiviral agents against the variants of concerns (VoCs). Interestingly, compound **1** showed a similar activity against the B.1 lineage and the B.1.1.7 (UK or Alpha variant) and B.1.617.2 (Indian or Delta variant) lineages in Vero E6 cells (Table 5). In addition, compound **1** is equally active against other β-coronaviruses, such as SARS-CoV-1 and the Middle East respiratory syndrome coronavirus (MERS-CoV).

#### 2.3.3. Mode of Action Investigation

In order to exclude a non-specific antiviral effect, we tested compound **1** against the SARS-CoV-2 unrelated Chikungunya virus in Vero A cells [27]. The complete lack of antiviral activity of compound **1** (EC_50_ > 100 µM) in this assay demonstrates a specific antiviral effect for coronavirus. However, since compounds were evaluated in a cell-based assay, the exact molecular target responsible for the antiviral activity remains elusive. In an effort to pinpoint the exact target, the inhibitory effect of compound **1** against various coronavirus enzymes such as SARS-CoV-2 Mpro, SARS-CoV-2 nsp14 (N7-methyltransferase activity), and the SARS-CoV-2 replication–transcription complex was studied. However, even at the highest tested concentration of 100 µM, compound **1** did not show any inhibitory activity on these enzymes.

To evaluate if compound **1** acted at the level of the virus entry, a SARS-CoV-2 and MERS pseudovirus neutralization assay was performed. In both assays, compound **1** was able to block the virus entry with an EC_50_ value of 1.25 µM and 3.4 µM, respectively. Compound **1** was found to be inactive against human proteases involved in SARS-CoV-2 entry such as cathepsin L, cathepsin B, TMPRSS2, and furin. Altogether, this first set of data demonstrated that hit **1** exerts its antiviral activity via interference with the viral entry mechanism. More research is required to determine if this virus entry inhibition is mediated by a viral or a host target protein.

## 3. Materials and Methods

### 3.1. Chemistry

#### 3.1.1. General Methods

All reagents and solvents were purchased from Sigma-Aldrich (Saint Louis, MO, USA), TCI (TCI Europe N.V., Zwijndrecht, Belgium), Combi-Blocks (San Diego, CA, USA) and used without further purification. All the reactions were monitored by thin-layer chromatography (TLC) or liquid chromatography–mass spectrometry (LCMS). TLC was carried out with Sigma-Aldrich silica gel (254 nm) plates (cat ref 99571-25EA) and TLC plates were revealed with UV light, KMnO_4_, *p*-anisaldehyde, or ninhydrin solutions. LCMS analysis was performed on an Agilent 1260 Infinity II UPLC machine (Agilent Technologies, Santa Clara, CA, USA) with a YMC-Triart C18 column (YMC CO., Kyoto, Japan). Flash chromatography purifications were performed on Biotage prepacked silica gel columns using Biotage Isolera instruments (Biotage, Upsala, Sweden). Reversed-phase preparative high-pressure liquid chromatography (HPLC) purification of final analogs was performed on a Waters Autopurification instrument (Waters Corporation, Milford, MA, USA) with MS- and UV-triggered collection operating at ambient temperature and at a flow rate of 16 mL/min. The identity of all compounds with reported biological activity was confirmed by NMR spectroscopy and low-resolution mass spectrometry (MS). The purity of all compounds with reported biological activity was ≥95% as determined by NMR and ultraperformance liquid chromatography (UPLC). Proton NMR spectra were recorded on a 300 or 400 MHz Bruker spectrometer (Bruker Corporation, Billerica, MA, USA) using TMS as internal standard, whereas carbon NMR spectra were recorded on a Bruker Avance 600 MHz instrument (Bruker Corporation, Billerica, MA, USA) (^13^C NMR, 150 MHz) using DMSO-*d*_6_ (39.5 ppm) as internal standard. Proton and carbon chemical shifts (δ) are reported in parts per million (ppm). Abbreviations used are: s = singlet, d = doublet, t = triplet, q = quartet, m = multiplet, br = broad. Coupling constants are expressed in Hz. Low-resolution mass spectral data were obtained using a Waters H-Class UPLC (Waters Corporation, Milford, MA, USA) with a Waters Acquity UPLC BEH C18 1.7 μm, 2.1 mm × 30 mm column (Waters Corporation, Milford, MA, USA), equipped with an Acquity UPLC PDA Detector (Waters Corporation, Milford, MA, USA), an Acquity UPLC ELS Detector (Waters Corporation, Milford, MA, USA)and an Acquity TQ Detector (ESI/ESCi) (Waters Corporation, Milford, MA, USA).

#### 3.1.2. 2-(3-(Pyridin-4-yl)-1,2,4-oxadiazol-5-yl)phenol (**4**)

A solution of *N*′-hydroxypyridine-4-carboxamidine (0.500 g, 3.65 mmol, 1 eq) and salicylaldehyde (0.461 mL, 4.38 mmol, 1.2 eq) in EtOH (1.5 mL) was irradiated in a microwave oven at 160 °C for 1 h. After cooling to room temperature, the beige solid was filtered, washed with cold ethanol and dried under high vacuum to afford 0.228 g (26%) of the title compound. LC-MS (ESI, *m*/*z*): 240 [M + H]^+^. ^1^H NMR (400 MHz, DMSO-*d*_6_) δ ppm 10.69 (s, 1H), 8.84 (d, *J* = 6.1 Hz, 2H), 8.02–8.05 (m, 3H), 7.52–7.60 (m, 1H), 7.15 (d, *J* = 8.5 Hz, 1H), 7.06 (t, *J* = 7.6 Hz, 1H).

#### 3.1.3. 5-(2-((1-Phenethylpyrrolidin-3-yl)oxy)phenyl)-3-(pyridin-4-yl)-1,2,4-oxadiazole (**1**)

A mixture of pyrrolidin-3-ol (0.100 g, 1.11 mmol, 1 eq), (2-bromoethyl) benzene (0.307 mL, 2.23 mmol, 2 eq) and K_2_CO_3_ (0.769 g, 5.57 mmol, 5 eq) in toluene (5 mL) was heated at 110 °C overnight. The reaction mixture was concentrated under reduced pressure. The residue was taken up with 1N HCl and extracted with CH_2_Cl_2_ twice. The aqueous phase was basified by the addition of K_2_CO_3_ and extracted with CH_2_Cl_2_. The organic phase was dried over MgSO_4_, filtered, and concentrated under reduced pressure to afford 0.130 g (61%) of 1-(2-phenylethyl)pyrrolidin-3-ol as a white solid. LC-MS (ESI, *m*/*z*): 192 [M + H]^+^. ^1^H NMR (400 MHz, DMSO-*d*_6_) δ ppm 7.12–7.33 (m, 5H), 4.67 (d, *J* = 4.6 Hz, 1H), 4.12–4.23 (m, 1H), 2.65–2.80 (m, 3H), 2.55–2.64 (m, 3H), 2.41–2.48 (m, 1H), 2.34 (m, 1H), 1.90–2.02 (m, 1H), 1.45–1.58 (m, 1H).

To a solution of **4** (0.050 g, 0.209 mmol, 1 eq), 1-(2-phenylethyl)pyrrolidin-3-ol (0.056 g, 0.293 mmol, 1.4 eq) and 1,1′-(azodicarbonyl)dipiperidine (0.075 g, 0.293 mmol, 1.4 eq) in THF (2.2 mL) was added *n*Bu_3_P (0.079 mL, 0.293 mmol, 1.4 eq). The reaction mixture was stirred at room temperature for 2 d and was concentrated under reduced pressure. The residue was purified by flash chromatography on silica gel using a gradient of MeOH (0–15%) in CH_2_Cl_2_. A second purification by flash chromatography on silica gel using a gradient of MeOH (0–7%) in CH_2_Cl_2_ followed by trituration with Et_2_O furnished 0.045 g (51%) of the title compound as a white solid. LC-MS (ESI, *m*/*z*): 413 [M + H]^+^. ^1^H NMR (400 MHz, DMSO-*d*_6_) δ 8.84 (d, *J* = 4.8 Hz, 2H), 8.12 (d, *J* = 7.3 Hz, 1H), 8.02 (d, *J* = 5.7 Hz, 2H), 7.68 (t, *J* = 7.8 Hz, 1H), 7.11–7.32 (m, 7H), 5.11 (m, 1H), 3.06 (m, 1H), 2.74–2.86 (m, 4H), 2.67 (m, 2H), 2.59 (m, 1H), 2.34 (m, 1H), 1.94 (m, 1H) (Figure S1). ^13^C NMR (150 MHz, DMSO-*d*_6_) δ 175.89, 166.29, 156.64, 150.99, 140.38, 135.10, 133.74, 131.63, 128.65, 128.27, 125.92, 121.08, 120.96, 114.81, 112.68, 77.72, 59.63, 57.32, 52.50, 34.53, 31.87 (Figure S2).

#### 3.1.4. 5-[2-[(3*R*)-1-(2-Phenylethyl)pyrrolidin-3-yl]oxyphenyl]-3-(4-pyridyl)-1,2,4-oxadiazole (**1a**)

The title compound was prepared similarly to **1** using (3S)-1-(2-phenylethyl)pyrrolidin-3-ol instead of pyrrolidin-3-ol (Figures S3 and S4).

#### 3.1.5. 5-[2-[(3S)-1-(2-Phenylethyl)pyrrolidin-3-yl]oxyphenyl]-3-(4-pyridyl)-1,2,4-oxadiazole (1b)

The title compound was prepared similarly to **1** using (3*R*)-1-(2-phenylethyl)pyrrolidin-3-ol instead of pyrrolidin-3-ol (Figure S5).

#### 3.1.6. 5-[2-(1-Cyclopentylpyrrolidin-3-yl)oxyphenyl]-3-(4-pyridyl)-1,2,4-oxadiazole (**8**)

A mixture of pyrrolidin-3-ol (0.100 g, 1.11 mmol, 1 eq), bromocyclopentane (0.244 mL, 2.23 mmol, 2 eq) and K_2_CO_3_ (0.769 g, 5.57 mmol, 5 eq) in toluene (5 mL) was heated at 110 °C overnight. The reaction mixture was concentrated under reduced pressure. The residue was taken up with 1N HCl and extracted with CH_2_Cl_2_ twice. The aqueous phase was basified by the addition of K_2_CO_3_ and extracted with CH_2_Cl_2_. The organic phase was dried over MgSO_4_, filtered, and concentrated under reduced pressure to afford 0.081 g (47%) of 1-cyclopentylpyrrolidin-3-ol as a white solid. LC-MS (ESI, *m*/*z*): 156 [M + H]^+^.

The title compound was prepared similarly to **1** using 1-cyclopentylpyrrolidin-3-ol instead of 1-(2-phenylethyl)pyrrolidin-3-ol. LC-MS (ESI, *m*/*z*): 377 [M + H]^+^. ^1^H NMR (400 MHz, DMSO-*d*_6_) δ 8.84 (m, 2H), 8.12 (dd, *J* = 1.5, 7.6 Hz, 1H), 8.02 (m, 2H), 7.68 (m, 1H), 7.26 (d, *J* = 8.5 Hz, 1H), 7.19 (t, *J* = 7.6 Hz, 1H), 5.07 (t, *J* = 6.7 Hz, 1H), 3.02 (m, 1H), 2.65–2.77 (m, 2H), 2.43–2.48 (m, 2H), 2.33 (m, 1H), 1.92 (m, 1H), 1.73 (m, 2H), 1.33–1.67 (m, 6H) (Figure S6).

#### 3.1.7. 5-[2-[1-(Cyclopentylmethyl)pyrrolidin-3-yl]oxyphenyl]-3-(4-pyridyl)-1,2,4-oxadiazole (**9**)

To a solution of **4** (0.165 g, 0.690 mmol, 1 eq) in THF (2.2 mL) were added DIAD (0.217 mL, 1.03 mmol, 1.5 eq), 1-Boc-3-hydroxypyrrolidine (0.200 g, 1.03 mmol, 1.5 eq) and PPh_3_ (0.271 g, 1.03 mmol, 1.5 eq). The reaction mixture was stirred at room temperature overnight. The reaction mixture was diluted with EtOAc and washed with water. The phases were separated. The organic phase was washed with 1N NaOH, dried over MgSO_4_, filtered, and concentrated under reduced pressure. The residue was purified by flash chromatography on silica gel using a gradient of EtOAc (20–60%) in heptane to afford 0.276 g (98%) of *tert*-butyl 3-[2-[3-(4-pyridyl)-1,2,4-oxadiazol-5-yl]phenoxy]pyrrolidine-1-carboxylate as a white solid. LC-MS (ESI, *m*/*z*): 409 [M + H]^+^.

To a mixture of *tert*-butyl 3-[2-[3-(4-pyridyl)-1,2,4-oxadiazol-5-yl]phenoxy]pyrrolidine-1-carboxylate (0.276 g, 0.676 mmol, 1 eq) in dioxane (2 mL) was added 4N HCl in dioxane (1.01 mL; 4.04 mmol, 6 eq). The reaction mixture was stirred at room temperature for 4 h and was concentrated under reduced pressure to afford quantitatively 3-(4-pyridyl)-5-(2-pyrrolidin-3-yloxyphenyl)-1,2,4-oxadiazole dihydrochloride as a white solid.

To a solution of 3-(4-pyridyl)-5-(2-pyrrolidin-3-yloxyphenyl)-1,2,4-oxadiazole dihydrochloride (0.100 g, 0.262 mmol, 1 eq) in DMF (1 mL) were added K_2_CO_3_ (0.073 g, 0.525 mmol, 2 eq) and iodomethylcyclopentane (0.053 mL, 0.393 mmol, 1.5 eq). The reaction mixture was heated at 80 °C for 2 h. After cooling to room temperature, the reaction mixture was diluted with water and extracted with CH_2_Cl_2_. The organic phase was dried over MgSO_4_, filtered, and concentrated under reduced pressure. The residue was purified by flash chromatography on silica gel using a gradient of MeOH (0–25%) in CH_2_Cl_2_. A second purification by flash chromatography on C18 using a gradient of CH_3_CN (5–100%) in water furnished 0.043 g (40%) of the title compound as a beige solid. LC-MS (ESI, *m*/*z*): 391 [M + H]^+^. ^1^H NMR (400 MHz, CD_3_OD) δ 8.78 (m, 2H), 8.14 (m, 3H), 7.66 (m, 1H), 7.20 (m, 2H), 5.12 (m, 1H), 3.14 (m, 1H), 2.93 (m, 1H), 2.85 (m, 1H), 2.70 (m, 1H), 2.45–2.60 (m, 2H), 2.40 (m, 1H), 2.01–2.18 (m, 2H), 1.83 (m, 2H), 1.50–1.72 (m, 4H), 1.24 (m, 2H) (Figure S7). ^13^C NMR (150 MHz, DMSO-*d*_6_) δ 175.55, 166.30, 156.68, 150.97, 135.07, 133.76, 131.57, 121.07, 120.91, 114.83, 112.70, 77.80, 61.46, 59.96, 52.84, 38.47, 31.86, 30.80, 24.81 (Figure S8).

#### 3.1.8. 5-[2-[(1-Cyclopentylpyrrolidin-3-yl)methoxy]phenyl]-3-(4-pyridyl)-1,2,4-oxadiazole (**10**)

To a solution of **4** (0.300 g, 1.25 mmol, 1 eq) in THF (4 mL) were added DIAD (0.394 mL, 1.88 mmol, 1.5 eq), *tert*-butyl 3-(hydroxymethyl)pyrrolidine-1-carboxylate (0.379 g, 1.88 mmol, 1.5 eq) and PPh_3_ (0.493 g, 1.88 mmol, 1.5 eq). The reaction mixture was stirred at room temperature overnight. The reaction mixture was diluted with EtOAc and washed with water. The phases were separated. The organic phase was washed with 1N NaOH, dried over MgSO_4_, filtered, and concentrated under reduced pressure. The residue was purified by flash chromatography on silica gel using a gradient of EtOAc (20–60%) in heptane to afford 0.529 g (99%) of *tert*-butyl 3-[[2-[3-(4-pyridyl)-1,2,4-oxadiazol-5-yl]phenoxy]methyl]pyrrolidine-1-carboxylate as a white solid. LC-MS (ESI, *m*/*z*): 423 [M + H]^+^.

To a mixture of *tert*-butyl 3-[[2-[3-(4-pyridyl)-1,2,4-oxadiazol-5-yl]phenoxy]methyl]pyrrolidine-1-carboxylate (0.529 g, 1.25 mmol, 1 eq) in dioxane (4 mL) was added 4N HCl in dioxane (1.01 mL; 4.04 mmol, 3.2 eq). The reaction mixture was stirred at room temperature overnight and was concentrated under reduced pressure to afford quantitatively 3-(4-pyridyl)-5-[2-(pyrrolidin-3-ylmethoxy)phenyl]-1,2,4-oxadiazole dihydrochloride as a white solid.

To a solution of 3-(4-pyridyl)-5-[2-(pyrrolidin-3-ylmethoxy)phenyl]-1,2,4-oxadiazole dihydrochloride (0.100 g, 0.253 mmol, 1 eq) in DMF (2 mL) were added K_2_CO_3_ (0.175 g, 1.26 mmol, 5 eq), NaI (0.019 g, 0.127 mmol, 0.5 eq) and bromocyclopentane (0.083 mL, 0.759 mmol, 3 eq). The reaction mixture was heated at 80 °C for 2 h. After cooling to room temperature, the reaction mixture was diluted with water and extracted with CH_2_Cl_2_. The organic phase was dried over MgSO_4_, filtered, and concentrated under reduced pressure. The residue was purified by flash chromatography on silica gel using a gradient of MeOH (0–25%) in CH_2_Cl_2_ to afford 0.026 g (26%) of the title compound as a beige solid. LC-MS (ESI, *m*/*z*): 391 [M + H]^+^. ^1^H NMR (400 MHz, CD_3_OD) δ 8.81 (d, *J* = 5.8 Hz, 2H), 8.24 (m, 1H), 8.14 (m, 2H), 7.71 (m, 1H), 7.33 (d, *J* = 8.5 Hz, 1H), 7.24 (t, *J* = 7.6 Hz, 1H), 4.27–4.39 (m, 2H), 3.75 (m, 1H), 3.36–3.62 (m, 4H), 3.07 (m, 1H), 2.38 (m, 1H), 2.18 (m, 3H), 1.86 (m, 2H), 1.71 (m, 4H) (Figure S9). ^13^C NMR (150 MHz, DMSO-*d*_6_) δ 175.57, 166.49, 157.41, 151.05, 135.29, 133.70, 131.31, 121.41, 121.12, 114.07, 112.23, 69.70, 65.77, 54.61, 52.10, 36.15, 29.03, 26.02, 23.58 (Figure S10).

#### 3.1.9. 5-[2-[[1-(Cyclopentylmethyl)pyrrolidin-3-yl]methoxy]phenyl]-3-(4-pyridyl)-1,2,4-oxadiazole (**11**)

The title compound was prepared similarly to **10** using iodomethylcyclopentane instead of bromocyclopentane. LC-MS (ESI, *m*/*z*): 405 [M + H]^+^. ^1^H NMR (400 MHz, CD_3_OD) δ 8.80 (m, 2H), 8.20 (dd, *J* = 1.5, 7.5 Hz, 1H), 8.15 (m, 2H), 7.68 (m, 1H), 7.30 (d, *J* = 8.5 Hz, 1H), 7.21 (t, *J* = 7.5 Hz, 1H), 4.15–do4.32 (m, 2H), 3.12 (m, 1H), 2.85–3.04 (m, 3H), 2.80 (m, 2H), 2.08–2.30 (m, 2H), 1.81–2.00 (m, 3H), 1.52–1.73 (m, 4H), 1.17–1.39 (m, 3H) (Figure S11). ^13^C NMR (150 MHz, DMSO-*d*_6_) δ 175.71, 166.40, 157.68, 151.00, 135.22, 133.74, 131.30, 121.17, 121.08, 114.06, 112.24, 71.06, 60.66, 56.60, 53.53, 37.78, 36.39, 30.77, 26.45, 24.75 (Figure S12).

#### 3.1.10. 5-[2-[(1-Cyclopentyl-4-piperidyl)oxy]phenyl]-3-(4-pyridyl)-1,2,4-oxadiazole (**12**)

To a solution of **4** (0.165 g, 0.690 mmol, 1 eq) in THF (2.2 mL) were added DIAD (0.217 mL, 1.03 mmol, 1.5 eq), 1-Boc-4-hydroxypiperidine (0.213 g, 1.03 mmol, 1.5 eq) and PPh_3_ (0.271 g, 1.03 mmol, 1.5 eq). The reaction mixture was stirred at room temperature overnight. The reaction mixture was diluted with EtOAc and washed with water. The phases were separated. The organic phase was washed with 1N NaOH, dried over MgSO_4_, filtered, and concentrated under reduced pressure. The residue was purified by flash chromatography on silica gel using a gradient of EtOAc (20–60%) in heptane to afford 0.276 g (95%) of *tert*-butyl 4-[2-[3-(4-pyridyl)-1,2,4-oxadiazol-5-yl]phenoxy]piperidine-1-carboxylate as a white solid. LC-MS (ESI, *m*/*z*): 423 [M + H]^+^.

To a mixture of *tert*-butyl 4-[[2-[3-(4-pyridyl)-1,2,4-oxadiazol-5-yl]phenoxy]piperidine-1-carboxylate (0.271 g, 0.642 mmol, 1 eq) in dioxane (2 mL) was added 4N HCl in dioxane (0.962 mL; 3.85 mmol, 6 eq). The reaction mixture was stirred at room temperature overnight and was concentrated under reduced pressure to afford quantitatively 5-[2-(4-piperidyloxy)phenyl]-3-(4-pyridyl)-1,2,4-oxadiazole dihydrochloride as a white solid.

To a solution of 5-[2-(4-piperidyloxy)phenyl]-3-(4-pyridyl)-1,2,4-oxadiazole dihydrochloride (0.100 g, 0.253 mmol, 1 eq) in DMF (2 mL) were added K_2_CO_3_ (0.175 g, 1.26 mmol, 5 eq), NaI (0.019 g, 0.127 mmol, 0.5 eq) and bromocyclopentane (0.083 mL, 0.759 mmol, 3 eq). The reaction mixture was heated at 80 °C for 2 h. After cooling to room temperature, the reaction mixture was diluted with water and extracted with CH_2_Cl_2_. The organic phase was dried over MgSO_4_, filtered, and concentrated under reduced pressure. The residue was purified by flash chromatography on silica gel using a gradient of MeOH (1–25%) in CH_2_Cl_2_ to afford 0.056 g (57%) of the title compound as a beige solid. LC-MS (ESI, *m*/*z*): 391 [M + H]^+^. ^1^H NMR (400 MHz, CD_3_OD) δ 8.80 (m, 2H), 8.21 (dd, *J* = 1.5, 7.9 Hz, 1H), 8.16 (m, 2H), 7.68 (m, 1H), 7.36 (d, *J* = 8.2 Hz, 1H), 7.22 (t, *J* = 7.9 Hz, 1H), 4.97 (m, 1H), 3.23 (m, 2H), 3.03–3.18 (m, 3H), 2.17 (m, 4H), 2.07 (m, 2H), 1.79 (m, 2H), 1.63 (m, 4H) (Figure S13). ^13^C NMR (150 MHz, DMSO-*d*_6_) δ 175.70, 166.48, 156.00, 151.00, 135.11, 133.78, 131.56, 121.29, 121.14, 115.45, 113.20, 71.21, 66.65, 47.47, 28.87, 23.66 (Figure S14).

#### 3.1.11. 5-(2-(2-Bromoethoxy)phenyl)-3-(pyridin-4-yl)-1,2,4-oxadiazole (**7**)

To a solution of **4** (3.0 g, 12.5 mmol, 1 eq) in DMF (30 mL) were added K_2_CO_3_ (3.5 g, 25.1 mmol, 2 eq) and 1,2-dibromoethane (3.24 g, 37.6 mmol, 3 eq). The reaction mixture was heated at 80 °C for 2 h. After cooling to room temperature, the reaction mixture was poured into ice/water. The resulting precipitate was filtered and dried under high vacuum to afford 2.0 g (46%) of the title compound as a beige solid. ^1^H NMR (300 MHz, DMSO-*d*_6_) δ 8.84 (d, *J* = 5.9 Hz, 2H), 8.12 (m, 1H), 8.03 (d, *J* = 5.8 Hz, 2H), 7.70 (m, 1H), 7.35 (d, *J* = 8.4 Hz, 1H), 7.23 (t, *J* = 7.5 Hz, 1H), 4.55 (t, *J* = 5.2 Hz, 2H), 3.88 (t, *J* = 5.2 Hz, 2H).

#### 3.1.12. N-[2-[2-[3-(4-Pyridyl)-1,2,4-oxadiazol-5-yl]phenoxy]ethyl]cyclopentanamine (13)

To a solution of **7** (0.200 g, 0.578 mmol, 1 eq) in DMF (2 mL) were added K_2_CO_3_ (0.160 g, 1.16 mmol, 2 eq) and cyclopentanamine (0.074 g, 0.867 mmol, 1.5 eq). The reaction mixture was heated at 80 °C for 2 h. After cooling to room temperature, the reaction mixture was diluted with water and extracted with 10% MeOH in CH_2_Cl_2_. The organic phase was washed with brine, dried over Na_2_SO_4_, filtered and concentrated under reduced pressure. The residue was purified by reversed-phase HPLC (Xbridge C18 column,250 × 19 mm, 10µ; 20 to 65% ACN in 20 mM ammonium bicarbonate in 35 min) to afford 0.040 g (20%) of the title compound as an off-white solid. LC-MS (ESI, *m*/*z*): 351 [M + H]^+^. ^1^H NMR (400 MHz, DMSO-*d*_6_) δ 8.84 (d, *J* = 4.9 Hz, 2H), 8.10 (d, *J* = 7.3 Hz, 1H), 8.00 (d, *J* = 4.9 Hz, 2H), 7.69 (t, *J* = 7.8 Hz, 1H), 7.33 (d, *J* = 7.8 Hz, 1H), 7.19 (t, *J* = 7.3 Hz, 1H), 4.23 (m, 2H), 3.09 (m, 1H), 2.94 (m, 2H), 1.70 (m, 2H), 1.58 (m, 2H), 1.44 (m, 2H), 1.28 (m, 2H) (NH not visible) (Figure S15). ^13^C NMR (150 MHz, DMSO-*d*_6_) δ 175.63, 166.45, 157.72, 150.95, 135.23, 133.73, 131.22, 121.09, 114.05, 112.21, 68.89, 58.94, 46.70, 32.80, 23.58 (Figure S16).

#### 3.1.13. N-Methyl-N-[2-[2-[3-(4-pyridyl)-1,2,4-oxadiazol-5-yl]phenoxy]ethyl]cyclopentanamine (**14**)

The title compound was prepared similarly to **13** using *N*-methylcyclopentanamine instead of cyclopentanamine. LC-MS (ESI, *m*/*z*): 365 [M + H]^+^. ^1^H NMR (400 MHz, DMSO-*d*_6_) δ 8.84 (d, *J* = 5.9 Hz, 2H), 8.08 (d, *J* = 7.3 Hz, 1H), 8.01 (d, *J* = 5.9 Hz, 2H), 7.69 (t, *J* = 7.8 Hz, 1H), 7.35 (d, *J* = 7.8 Hz, 1H), 7.18 (t, *J* = 7.3 Hz, 1H), 4.24 (m, 2H), 2.83 (m, 2H), 2.75 (m, 1H), 2.26 (s, 3H), 1.71 (m, 2H), 1.54 (m, 2H), 1.43 (m, 2H), 1.33 (m, 2H) (Figure S17). ^13^C NMR (150 MHz, DMSO-*d*_6_) δ 175.83, 166.36, 157.75, 150.98, 135.12, 133.73, 131.36, 121.08, 120.93, 113.94, 112.30, 67.65, 66.21, 53.98, 40.80, 29.92, 23.84 (Figure S18).

#### 3.1.14. *tert*-Butyl 3-(2-(methoxycarbonyl)phenoxy)pyrrolidine-1-carboxylate (**17**)

To a solution of DIAD (8.8 mL, 44.9 mmol, 1.2 eq) in THF (20 mL) cooled at 0 °C was added PPh_3_ (10.5 g, 41.1 mmol, 1.1 eq). The reaction mixture was stirred at 0 °C for 30 min. *tert*-Butyl 3-hydroxypyrrolidine-1-carboxylate (7.0 g, 37.4 mmol, 1 eq) was added and the reaction mixture was stirred at 0 °C for 15 min. Methyl 2-hydroxybenzoate (5.7 g, 37.4 mmol, 1 eq) was added and the reaction mixture was stirred at room temperature overnight. The reaction mixture was concentrated under reduced pressure. The residue was partitioned between water and EtOAc. The phases were separated. The aqueous phase was extracted twice with EtOAc. The organic phases were combined, dried over Na_2_SO_4_, filtered, and concentrated under reduced pressure. The residue was purified by flash chromatography on silica gel using a gradient of EtOAc (0–50%) in hexane to afford 4.0 g (33%) of the title compound as a colorless liquid. ^1^H NMR (400 MHz, CDCl_3_) δ 7.77 (dd, *J* = 1.6, 7.6 Hz, 1H), 7.42 (m, 1H), 7.00 (t, *J* = 7.6 Hz, 1H), 6.91 (d, *J* = 8.4 Hz, 1H), 4.94 (m, 1H), 3.85 (s, 3H), 3.52–3.60 (m, 4H), 2.22 (m, 1H), 2.07 (m, 1H), 1.45 (s, 9H).

#### 3.1.15. Methyl 2-(pyrrolidin-3-yloxy)benzoate hydrochloride (**18**)

To a solution of **17** (12.8 g, 39.7 mmol, 1 eq) in CH_2_Cl_2_ (130 mL) cooled at 0 °C was added 4N HCl in dioxane (30 mL, 120 mmol, 3 eq). The reaction mixture was stirred at room temperature overnight and was concentrated under reduced pressure to afford quantitatively the title compound as a brown oil. ^1^H NMR (400 MHz, DMSO-*d*_6_) δ 9.56 (br. s., 1H), 9.30 (br. s., 1H), 7.70 (dd, *J* = 1.6, 7.6 Hz, 1H), 7.56 (t, *J* = 8.4 Hz, 1H), 7.23 (d, *J* = 8.4 Hz, 1H), 7.09 (t, *J* = 7.6 Hz, 1H), 5.22 (m, 1H), 3.81 (s, 3H), 3.49 (m, 1H), 3.15–3.40 (m, 3H), 2.14 (m, 2H).

#### 3.1.16. Methyl 2-((1-cyclopentylpyrrolidin-3-yl)oxy)benzoate (**19**)

To a solution of **18** (11.4 g, 44.2 mmol, 1 eq) in CH_2_Cl_2_ (120 mL) was added cyclopentanone (5.1 mL, 57.5 mmol, 1.3 eq) and the reaction mixture was stirred at room temperature for 3 h. NaBH(OAc)_3_ (14.1 g, 66.4 mmol, 1.5 eq) was added portion-wise and the reaction mixture was stirred at room temperature overnight. The reaction mixture was washed with water and the phases were separated. The aqueous phase was extracted with 10% MeOH in CH_2_Cl_2_ (3×). The organic phases were combined, dried over Na_2_SO_4_, filtered, and concentrated under reduced pressure. The residue was purified by flash chromatography on silica gel using a gradient of MeOH (0–10%) in CH_2_Cl_2_ to afford 9.9 g (77%) of the title compound as a brown oil. ^1^H NMR (400 MHz, CDCl_3_) δ 7.77 (d, *J* = 6.8 Hz, 1H), 7.43 (t, *J* = 7.2 Hz, 1H), 6.99 (t, *J* = 7.6 Hz, 1H), 6.89 (d, *J* = 8.4 Hz, 1H), 5.00 (m, 1H), 3.85 (s, 3H), 3.74 (m, 1H), 3.29 (m, 1H), 3.10 (m, 1H), 2.92–3.02 (m, 2H), 2.34 (m, 1H), 2.15 (m, 1H), 1.93 (m, 3H), 1.76 (m, 3H), 1.55 (m, 2H).

#### 3.1.17. Lithium 2-((1-cyclopentylpyrrolidin-3-yl)oxy)benzoate (**20**)

To a solution of **19** (1.0 g, 3.46 mmol, 1 eq) in THF (30 mL), MeOH (10 mL) and water (20 mL) was added LiOH (0.138 g, 3.28 mmol, 0.9 eq). The reaction mixture was heated at 60 °C for 6 h and was concentrated under reduced pressure. The residue was dissolved in water (30 mL) and was washed twice with CH_2_Cl_2_. The aqueous phase was lyophilized to afford 0.800 g (82%) of lithium 2-((1-cyclopentylpyrrolidin-3-yl)oxy)benzoate as an off-white solid. ^1^H NMR (400 MHz, DMSO-*d*_6_) δ 7.15 (d, *J* = 7.2 Hz, 1H), 7.04 (t, *J* = 7.6 Hz, 1H), 6.75 (m, 2H), 4.75 (m, 1H), 2.80 (m, 1H), 2.57–2.66 (m, 2H), 2.41 (m, 2H), 2.13 (m, 1H), 1.70–1.85 (m, 3H), 1.58–1.64 (m, 2H), 1.30–1.55 (m, 4H).

#### 3.1.18. 5-[2-(1-Cyclopentylpyrrolidin-3-yl)oxyphenyl]-3-(3-pyridyl)-1,2,4-oxadiazole (**23**)

To a solution of *N*-hydroxynicotinimidamide (0.150 g, 1.09 mmol, 1 eq) in DMSO (2 mL) were added **19** (0.474 g, 1.64 mmol, 1.5 eq) and NaOH (0.066 g, 1.64 mmol, 1.5 eq). The reaction mixture was stirred at room temperature overnight. The reaction mixture was diluted with water and extracted with EtOAc. The organic phase was dried over Na_2_SO_4_, filtered, and concentrated under reduced pressure. The residue was purified by flash chromatography on silica gel using a gradient of MeOH (10–20%) in EtOAc. A second purification by reversed-phase HPLC (Xterra RP18 column,250 × 19 mm, 10µ; 20 to 85% MeOH in 20 mM ammonium bicarbonate in 24 min) furnished 0.015 g (4%) of the title compound as an off-white solid. LC-MS (ESI, *m*/*z*): 377 [M + H]^+^. ^1^H NMR (400 MHz, DMSO-*d*_6_) δ 9.26 (s, 1H), 8.81 (d, *J* = 3.9 Hz, 1H), 8.44 (d, *J* = 7.3 Hz, 1H), 8.12 (d, *J* = 7.3 Hz, 1H), 7.67 (m, 2H), 7.09–7.38 (m, 2H), 5.07 (m, 1H), 3.01 (m, 1H), 2.70 (m, 2H), 2.33 (m, 2H), 1.89 (m, 1H), 1.73 (m, 2H), 1.60 (m, 2H), 1.31–1.54 (m, 5H) (Figure S19).

#### 3.1.19. 5-[2-(1-Cyclopentylpyrrolidin-3-yl)oxyphenyl]-3-pyrimidin-5-yl-1,2,4-oxadiazole (**24**)

A mixture of 20 (0.250 g, 0.889 mmol, 1 eq), HATU (0.406 g, 1.07 mmol, 1.2 eq) and DIPEA (0.372 mL, 2.14 mmol, 2.4 eq) in DMF (3 mL) was stirred at room temperature for 15 min. After the addition of *N*-hydroxypyrimidine-5-carboximidamide (0.123 g, 0.890 mmol, 1 eq) [28], the reaction mixture was stirred at room temperature for 6 h and was heated at 100 °C overnight. After cooling to room temperature, the reaction mixture was diluted with CH_2_Cl_2_ and was washed with sat. NaHCO_3_. The phases were separated. The organic phase was washed with sat. NaHCO_3_, dried over Na_2_SO_4_, filtered, and concentrated under reduced pressure. The residue was purified by reversed-phase HPLC (YMC-Actus C18 column,250 × 20 mm, 5µ; 40 to 90% ACN in 20 mM ammonium bicarbonate in 20 min) to afford 0.050 g (15%) of the title compound as an off-white solid. LC-MS (ESI, *m*/*z*): 378 [M + H]^+^. ^1^H NMR (400 MHz, CD_3_OD) δ 9.46 (s, 2H), 9.33 (s, 1H), 8.16 (dd, *J* = 1.5, 7.8 Hz, 1H), 7.64 (m, 1H), 7.10–7.24 (m, 2H), 5.11 (m, 1H), 3.16 (m, 1H), 2.84–3.04 (m, 2H), 2.69 (m, 2H), 2.39 (m, 1H), 2.13 (m, 1H), 1.89 (m, 2H), 1.71 (m, 2H), 1.39–1.64 (m, 4H) (Figure S20). ^13^C NMR (150 MHz, DMSO-*d*_6_) δ 175.78, 163.96, 160.47, 156.72, 155.31, 135.21, 131.64, 121.45, 120.93, 114.82, 112.49, 77.78, 66.17, 59.11, 51.69, 31.87, 31.58, 31.41, 23.71 (Figure S21).

#### 3.1.20. 5-[2-(1-Cyclopentylpyrrolidin-3-yl)oxyphenyl]-3-pyridazin-4-yl-1,2,4-oxadiazole (**25**)

A mixture of pyridazine-4-carbonitrile (1.0 g, 9.52 mmol, 1 eq), hydroxylamine hydrochloride (0.992 g, 14.3 mmol, 1.5 eq) and DIPEA (3.3 mL, 19.0 mmol, 2 eq) in EtOH (40 mL) was heated at 90 °C overnight. The reaction mixture was concentrated under reduced pressure. The residue was partitioned between EtOAc and water. The phases were separated. The organic phase was dried over Na_2_SO_4_, filtered, and concentrated under reduced pressure. The residue was purified by flash chromatography on silica gel using a gradient of THF (0–70%) in hexane to afford 0.300 g (23%) of *N*-hydroxypyridazine-4-carboximidamide as an off-white solid. LC-MS (ESI, *m*/*z*): 139 [M + H]^+^. ^1^H NMR (400 MHz, DMSO-*d*_6_) δ 10.36 (s, 1H), 9.45 (s, 1H), 9.25 (d, *J* = 6.1 Hz, 1H), 7.86 (m, 1H), 6.19 (br. s., 2H).

The title compound was prepared similarly to 24 using *N*-hydroxypyridazine-4-carboximidamide instead of *N*-hydroxypyrimidine-5-carboximidamide. LC-MS (ESI, *m*/*z*): 378 [M + H]^+^. ^1^H NMR (400 MHz, DMSO-*d*_6_) δ 9.81 (s, 1H), 9.53 (d, *J* = 4.9 Hz, 1H), 8.27 (d, *J* = 4.9 Hz, 1H), 8.14 (d, *J* = 7.8 Hz, 1H), 7.69 (t, *J* = 7.8 Hz, 1H), 7.26 (d, *J* = 7.8 Hz, 1H), 7.19 (m, 1H), 5.08 (m, 1H), 3.02 (m, 1H), 2.70 (m, 2H), 2.41–2.62 (m, 2H), 2.33 (m, 1H), 1.90 (m, 1H), 1.73 (m, 2H), 1.61 (m, 2H), 1.32–1.55 (m, 4H) (Figure S22). ^13^C NMR (150 MHz, DMSO-*d*_6_) δ 176.17, 164.64, 156.77, 152.60, 148.23, 135.34, 131.67, 125.08, 123.92, 120.96, 114.83, 112.37, 77.81, 66.15, 59.10, 51.69, 31.87, 31.59, 31.41, 23.71 (Figure S23).

#### 3.1.21. 5-[2-(1-Cyclopentylpyrrolidin-3-yl)oxyphenyl]-3-phenyl-1,2,4-oxadiazole (**26**)

The title compound was prepared similarly to 23 using *N*-hydroxybenzimidamide instead of *N*-hydroxynicotinimidamide. LC-MS (ESI, *m*/*z*): 376 [M + H]^+^. ^1^H NMR (400 MHz, DMSO-*d*_6_) δ 8.10 (m, 3H), 7.51–7.80 (m, 4H), 7.24 (d, *J* = 8.8 Hz, 1H), 7.17 (t, *J* = 7.6 Hz, 1H), 5.06 (m, 1H), 3.01 (m, 1H), 2.64–2.84 (m, 2H), 2.43–2.55 (m, 2H), 2.31 (m, 1H), 1.89 (m, 1H), 1.73 (m, 2H), 1.60 (m, 2H), 1.32–1.54 (m, 4H) (Figure S24). ^13^C NMR (150 MHz, DMSO-*d*_6_) δ 175.14, 167.59, 156.60, 134.78, 131.60, 131.50, 129.32, 127.12, 126.48, 120.86, 114.78, 113.03, 77.73, 66.14, 59.10, 51.67, 31.88, 31.57, 31.40, 23.70 (Figure S25).

#### 3.1.22. 5-[2-(1-Cyclopentylpyrrolidin-3-yl)oxyphenyl]-3-(6-methoxy-3-pyridyl)-1,2,4-oxadiazole (**27**)

A mixture of 6-methoxynicotinonitrile (1.0 g, 7.45 mmol, 1 eq), hydroxylamine hydrochloride (0.777 g, 11.2 mmol, 1.5 eq) and K_2_CO_3_ (3.09 g, 22.4 mmol, 3 eq) in EtOH (50 mL) was heated at 80 °C overnight. The reaction mixture was concentrated under reduced pressure. The residue was partitioned between EtOAc and water. The phases were separated. The organic phase was dried over Na_2_SO_4_, filtered, and concentrated under reduced pressure. The residue was purified by flash chromatography on silica gel using a gradient of THF (0–40%) in hexane to afford 0.200 g (16%) of *N*-hydroxy-6-methoxynicotinimidamide as an off-white solid. LC-MS (ESI, *m*/*z*): 168 [M + H]^+^. ^1^H NMR (400 MHz, DMSO-*d*_6_) δ 9.62 (s, 1H), 8.43 (s, 1H), 7.93 (m, 1H), 6.83 (d, *J* = 8.4 Hz, 1H), 5.86 (br. s., 2H), 3.86 (s, 3H).

The title compound was prepared similarly to **24** using *N*-hydroxy-6-methoxynicotinimidamide instead of *N*-hydroxypyrimidine-5-carboximidamide. LC-MS (ESI, *m*/*z*): 407 [M + H]^+^. ^1^H NMR (400 MHz, DMSO-*d*_6_) δ 8.88 (d, *J* = 2.0 Hz, 1H), 8.32 (dd, *J* = 2.0, 8.5 Hz, 1H), 8.09 (d, *J* = 7.5 Hz, 1H), 7.66 (t, *J* = 7.8 Hz, 1H), 7.24 (d, *J* = 7.8 Hz, 1H), 7.17 (t, *J* = 7.5 Hz, 1H), 7.04 (d, *J* = 8.5 Hz, 1H), 5.06 (m, 1H), 3.96 (s, 3H), 3.02 (m, 1H), 2.70 (m, 2H), 2.41–2.62 (m, 2H), 2.26–2.37 (m, 1H), 1.88 (m, 1H), 1.73 (m, 2H), 1.61 (m, 2H), 1.33–1.55 (m, 4H) (Figure S26).

#### 3.1.23. 5-[2-(1-Cyclopentylpyrrolidin-3-yl)oxyphenyl]-3-[5-(trifluoromethyl)-3-pyridyl]-1,2,4-oxadiazole (**28**)

A mixture of 5-(trifluoromethyl)nicotinonitrile (0.250 g, 1.45 mmol, 1 eq), hydroxylamine hydrochloride (0.151 g, 2.18 mmol, 1.5 eq) and DIPEA (0.600 mL, 3.6 mmol) in EtOH (5 mL) was heated at 90 °C overnight. The reaction mixture was concentrated under reduced pressure. The residue was partitioned between EtOAc and water. The phases were separated. The organic phase was dried over Na_2_SO_4_, filtered, and concentrated under reduced pressure. The residue was triturated with EtOAc. The white solid was filtered and dried under high vacuum to afford 0.250 g (83%) of *N*-hydroxy-5-(trifluoromethyl)nicotinimidamide. LC-MS (ESI, *m*/*z*): 206 [M + H]^+^. ^1^H NMR (400 MHz, DMSO-*d*_6_) δ 10.09 (s, 1H), 9.15 (s, 1H), 8.96 (s, 1H), 8.36 (s, 1H), 6.18 (br. s., 2H).

The title compound was prepared similarly to **24** using *N*-hydroxy-5-(trifluoromethyl)nicotinimidamide instead of *N*-hydroxypyrimidine-5-carboximidamide. LC-MS (ESI, *m*/*z*): 445 [M + H]^+^. ^1^H NMR (400 MHz, DMSO-*d*_6_) δ 9.53 (s, 1H), 9.25 (s, 1H), 8.70 (s, 1H), 8.15 (d, *J* = 7.3 Hz, 1H), 7.68 (t, *J* = 7.8 Hz, 1H), 7.26 (d, *J* = 7.8 Hz, 1H), 7.19 (t, *J* = 7.3 Hz, 1H), 5.08 (m, 1H), 3.03 (m, 1H), 2.65–2.77 (m, 2H), 2.41–2.62 (m, 2H), 2.33 (m, 1H), 1.89 (m, 1H), 1.73 (m, 2H), 1.60 (m, 2H), 1.31–1.55 (m, 4H) (Figure S27). ^13^C NMR (150 MHz, DMSO-*d*_6_) δ 175.76, 164.91, 156.73, 151.54, 148.85, 135.23, 131.75, 131.65, 123.17, 120.93, 114.83, 112.47, 77.79, 66.19, 59.14, 51.70, 31.89, 31.58, 31.41, 23.69 (Figure S28).

#### 3.1.24. 5-[2-(1-Cyclopentylpyrrolidin-3-yl)oxyphenyl]-3-[4-(trifluoromethyl)-3-pyridyl]-1,2,4-oxadiazole (**29**)

A mixture of 4-(trifluoromethyl)nicotinonitrile (0.250 g, 1.45 mmol, 1 eq), hydroxylamine hydrochloride (0.151 g, 2.18 mmol, 1.5 eq) and DIPEA (0.506 mL, 2.91 mmol, 2 eq) in EtOH (10 mL) was heated at 90 °C overnight. The reaction mixture was concentrated under reduced pressure. The residue was partitioned between EtOAc and water. The phases were separated. The organic phase was dried over Na_2_SO_4_, filtered, and concentrated under reduced pressure. The residue was purified by flash chromatography on silica gel using a gradient of THF (0–30%) in hexane to afford 0.140 g (47%) of *N*-hydroxy-4-(trifluoromethyl)nicotinimidamide as an off-white solid. LC-MS (ESI, *m*/*z*): 206 [M + H]^+^. ^1^H NMR (400 MHz, DMSO-*d*_6_) δ 9.76 (s, 1H), 8.85 (d, *J* = 4.9 Hz, 1H), 8.75 (s, 1H), 7.78 (d, *J* = 4.9 Hz, 1H), 6.04 (br. s., 2H).

The title compound was prepared similarly to **24** using *N*-hydroxy-4-(trifluoromethyl)nicotinimidamide instead of *N*-hydroxypyrimidine-5-carboximidamide. LC-MS (ESI, *m*/*z*): 445 [M + H]^+^. ^1^H NMR (400 MHz, DMSO-*d*_6_) δ 9.22 (s, 1H), 9.11 (d, *J* = 4.5 Hz, 1H), 8.06 (m, 2H), 7.68 (t, *J* = 7.3 Hz, 1H), 7.26 (d, *J* = 8.3 Hz, 1H), 7.18 (t, *J* = 7.3 Hz, 1H), 5.08 (m, 1H), 3.03 (m, 1H), 2.70 (m, 2H), 2.41–2.62 (m, 2H), 2.33 (m, 1H), 1.89 (m, 1H), 1.72 (m, 2H), 1.60 (m, 2H), 1.30–1.54 (m, 4H) (Figure S29). ^13^C NMR (150 MHz, DMSO-*d*_6_) δ 175.38, 164.73, 156.73, 153.66, 151.87, 135.23, 131.57, 125.08, 120.94, 120.73, 120.70, 119.84, 114.79, 112.34, 77.75, 66.12, 59.02, 51.65, 31.82, 31.52, 31.37, 23.67 (Figure S30).

#### 3.1.25. 5-[2-(1-Cyclopentylpyrrolidin-3-yl)oxyphenyl]-3-(4-methyl-3-pyridyl)-1,2,4-oxadiazole (**30**)

A mixture of 4-methylnicotinonitrile (0.500 g, 4.23 mmol, 1 eq), hydroxylamine hydrochloride (0.441 g, 6.35 mmol, 1.5 eq) and NEt_3_ (1.2 mL, 8.47 mmol, 2 eq) in EtOH (10 mL) was stirred at room temperature overnight. The reaction mixture was concentrated under reduced pressure. The residue was purified by flash chromatography on silica gel using a gradient of THF (0–40%) in hexane to afford 0.375 g (58%) of *N*-hydroxy-4-methylnicotinimidamide as an off-white solid. LC-MS (ESI, *m*/*z*): 152 [M + H]^+^. ^1^H NMR (400 MHz, DMSO-*d*_6_) δ 9.52 (s, 1H), 8.41 (m, 2H), 7.26 (d, *J* = 5.0 Hz, 1H), 5.89 (br. s., 2H), 2.36 (s, 3H).

The title compound was prepared similarly to **24** using *N*-hydroxy-4-methylnicotinimidamide instead of *N*-hydroxypyrimidine-5-carboximidamide. LC-MS (ESI, *m*/*z*): 391 [M + H]^+^. ^1^H NMR (400 MHz, DMSO-*d*_6_) δ 9.11 (s, 1H), 8.62 (d, *J* = 5.4 Hz, 1H), 8.11 (d, *J* = 7.8 Hz, 1H), 7.66 (t, *J* = 8.3 Hz, 1H), 7.49 (d, *J* = 5.4 Hz, 1H), 7.25 (d, *J* = 8.3 Hz, 1H), 7.17 (t, *J* = 7.8 Hz, 1H), 5.07 (m, 1H), 3.02 (m, 1H), 2.71 (m, 2H), 2.65 (s, 3H), 2.41–2.62 (m, 2H), 2.33 (m, 1H), 1.89 (m, 1H), 1.72 (m, 2H), 1.60 (m, 2H), 1.31–1.54 (m, 4H) (Figure S31). ^13^C NMR (150 MHz, DMSO-*d*_6_) δ 174.44, 166.42, 156.62, 151.34, 149.79, 146.79, 134.91, 131.59, 126.30, 122.42, 120.87, 120.70, 114.74, 112.73, 77.68, 66.16, 59.10, 51.70, 31.87, 31.56, 31.41, 23.69, 20.99 (Figure S32).

#### 3.1.26. 5-[2-(1-Cyclopentylpyrrolidin-3-yl)oxyphenyl]-3-(3-methyl-4-pyridyl)-1,2,4-oxadiazole (**31**)

A mixture of 3-methylisonicotinonitrile (0.250 g, 2.11 mmol, 1 eq), hydroxylamine hydrochloride (0.294 g, 4.24 mmol, 2 eq) and NaHCO_3_ (0.712 g, 8.48 mmol, 4 eq) in MeOH (5 mL) was heated at 70 °C for 6 h. The reaction mixture was concentrated under reduced pressure. The residue was purified by reversed-phase HPLC (YMC-Actus C18 column, 250 × 20 mm, 5 µ; 5% MeOH in 20 mM ammonium bicarbonate) to afford 0.180 g (56%) of *N*-hydroxy-3-methylisonicotinimidamide as an off-white solid. LC-MS (ESI, *m*/*z*): 152 [M + H]^+^. ^1^H NMR (400 MHz, DMSO-*d*_6_) δ 9.65 (s, 1H), 8.47 (s, 1H), 8.40 (d, *J* = 4.8 Hz, 1H), 7.29 (d, *J* = 4.8 Hz, 1H), 5.88 (br. s., 2H), 2.35 (s, 3H).

The title compound was prepared similarly to **24** using *N*-hydroxy-3-methylisonicotinimidamide instead of *N*-hydroxypyrimidine-5-carboximidamide. LC-MS (ESI, *m*/*z*): 391 [M + H]^+^. ^1^H NMR (400 MHz, DMSO-*d*_6_) δ 8.70 (s, 1H), 8.64 (d, *J* = 5.0 Hz, 1H), 8.11 (d, *J* = 7.5 Hz, 1H), 7.96 (d, *J* = 5.0 Hz, 1H), 7.67 (t, *J* = 7. 8 Hz, 1H), 7.26 (d, *J* = 7.5 Hz, 1H), 7.18 (t, *J* = 7.5 Hz, 1H), 5.08 (m, 1H), 3.03 (m, 1H), 2.71 (m, 2H), 2.63 (s, 3H), 2.41–2.62 (m, 2H), 2.33 (m, 1H), 1.90 (m, 1H), 1.74 (m, 2H), 1.61 (m, 2H), 1.31–1.55 (m, 4H) (Figure S33). ^13^C NMR (150 MHz, DMSO-*d*_6_) δ 174.72, 166.87, 156.62, 152.39, 147.93, 135.03, 132.86, 131.85, 131.60, 122.75, 120.93, 114.78, 112.63, 77.66, 66.15, 59.04, 51.69, 31.83, 31.50, 31.34, 23.69, 18.49 (Figure S34).

#### 3.1.27. 2-((1-Cyclopentylpyrrolidin-3-yl)oxy)benzonitrile (**33**)

To a solution of **19** (1.6 g, 5.53 mmol, 1 eq) in dioxane (5 mL) was added 25% NH_4_OH (10 mL; 79.3 mmol, 14 eq). The reaction mixture was heated at 100 °C overnight. The reaction mixture was concentrated under reduced pressure. The residue was partitioned between 10% LiOH and 10% MeOH in CH_2_Cl_2_. The phases were separated. The aqueous phase was extracted with 10% MeOH in CH_2_Cl_2_. The organic phases were combined, dried over Na_2_SO_4_, filtered, and concentrated under reduced pressure to afford 0.900 g of 2-((1-cyclopentylpyrrolidin-3-yl)oxy)benzamide **32**, which was used in the next step without further purification.

To a solution of **32** (0.900 g, 3.28 mmol) in EtOAc (20 mL) was added 50% T3P in EtOAc (4.8 mL, 16.4 mmol, 5 eq). The reaction mixture was heated at 60 °C overnight. The reaction mixture was concentrated under reduced pressure. The residue was partitioned between water and 10% MeOH in CH_2_Cl_2_. The phases were separated. The aqueous phase was extracted twice with 10% MeOH in CH_2_Cl_2_. The organic phases were combined, dried over Na_2_SO_4_, filtered, and concentrated under reduced pressure. The residue was purified by flash chromatography on silica gel using a gradient of MeOH (0–5%) in CH_2_Cl_2_ to afford 0.800 g (54% over two steps) of the title compound as a brown oil. LC-MS (ESI, *m*/*z*): 257 [M + H]^+^.

#### 3.1.28. 3-[2-(1-Cyclopentylpyrrolidin-3-yl)oxyphenyl]-5-(4-pyridyl)-1,2,4-oxadiazole (**35**)

A mixture of 2-((1-cyclopentylpyrrolidin-3-yl)oxy)benzonitrile (0.700 g, 2.73 mmol, 1 eq), hydroxylamine hydrochloride (0.569 g, 8.19 mmol, 3 eq) and DIPEA (1.9 mL, 10.9 mmol, 4 eq) in EtOH (10 mL) was heated at 70 °C overnight. After cooling to room temperature, the reaction mixture was diluted with water and extracted with 10% MeOH in CH_2_Cl_2_ (3×). The organic phases were combined, dried over Na_2_SO_4_, filtered, and concentrated under reduced pressure to afford 0.700 g (89%) of 2-((1-cyclopentylpyrrolidin-3-yl)oxy)-*N*-hydroxybenzimidamide **34** as an off-white solid, which was used in the next step without further purification. LC-MS (ESI, *m*/*z*): 290.

To a solution of **34** (0.700 g, 2.42 mmol, 1 eq) in DMF (10 mL) were added isonicotinic acid (0.596 g, 4.84 mmol, 2 eq), EDC.HCl (0.927 g, 4.84 mmol, 2 eq), HOBt (0.654 g, 4.84 mmol, 2 eq) and DIPEA (1.7 mL, 9.68 mmol, 4 eq). The reaction mixture was stirred at room temperature for 16 h and was heated at 80 °C for 16 h. After cooling to room temperature, the reaction mixture was partitioned between water and EtOAc. The phases were separated. The organic phase was washed with brine, dried over Na_2_SO_4_, filtered and concentrated under reduced pressure. The residue was purified by reversed-phase HPLC (Xbridge C18 column, 250 × 19 mm, 10µ; 30 to 75% ACN in 20 mM ammonium bicarbonate in 22 min) to afford 0.080 g (9%) of the title compound as a white solid. LC-MS (ESI, *m*/*z*): 377 [M + H]^+^. ^1^H NMR (400 MHz, DMSO-*d*_6_) δ 8.91 (d, *J* = 5.9 Hz, 2H), 8.10 (d, *J* = 5.9 Hz, 2H), 7.98 (d, *J* = 7.4 Hz, 1H), 7.57 (t, *J* = 7.1 Hz, 1H), 7.15 (m, 2H), 5.01 (m, 1H), 3.02 (m, 1H), 2.65 (m, 2H), 2.42–2.55 (m, 2H), 2.27 (m, 1H), 1.86 (m, 1H), 1.70 (m, 2H), 1.59 (m, 2H), 1.29–1.53 (m, 4H) (Figure S35). ^13^C NMR (150 MHz, DMSO-*d*_6_) δ 172.53, 167.26, 156.23, 151.25, 132.99, 131.10, 130.54, 121.35, 120.62, 115.40, 114.33, 77.46, 66.15, 59.07, 51.65, 31.85, 31.55, 31.40, 23.69 (Figure S36).

#### 3.1.29. 2-[2-(1-Cyclopentylpyrrolidin-3-yl)oxyphenyl]-5-(4-pyridyl)-1,3,4-oxadiazole (**38**)

To a solution of **36** (0.150 g, 0.545 mmol, 1 eq) in POCl_3_ (5 mL) was added isonicotinohydrazide (0.075 g, 0.545 mmol, 1 eq). The reaction mixture was heated at 90 °C overnight. The reaction mixture was concentrated under reduced pressure. The residue was taken up with sat. NaHCO_3_ and was extracted twice with 5% MeOH in CH_2_Cl_2_. The organic phases were combined, washed with brine, dried over Na_2_SO_4_, filtered, and concentrated under reduced pressure. The residue was purified by reversed-phase HPLC (YMC-Actus C18 column, 250 × 20 mm, 5µ; 30 to 75% ACN in 20 mM ammonium bicarbonate in 20 min) to afford 0.040 g (19%) of the title compound as yellow solid. LC-MS (ESI, *m*/*z*): 377 [M + H]^+^. ^1^H NMR (400 MHz, DMSO-*d*_6_) δ 8.85 (m, 2H), 8.01 (m, 3H), 7.62 (t, *J* = 7.3 Hz, 1H), 7.24 (d, *J* = 8.3 Hz, 1H), 7.16 (t, *J* = 7.3 Hz, 1H), 5.07 (m, 1H), 2.91 (m, 1H), 2.79 (m, 2H), 2.41–2.62 (m, 2H), 2.34 (m, 1H), 1.88 (m, 1H), 1.72 (m, 2H), 1.28–1.65 (m, 6H) (Figure S37). ^13^C NMR (150 MHz, DMSO-*d*_6_) δ 164.10, 162.43, 155.82, 151.02, 133.97, 130.70, 130.55, 120.87, 120.12, 114.65, 112.52, 77.58, 66.24, 59.31, 51.83, 31.91, 31.60, 31.40, 23.67 (Figure S38).

#### 3.1.30. 3-Bromo-5-(2-methoxyphenyl)-1,2,4-thiadiazole (**41**)

A mixture of 3-bromo-5-chloro-1,2,4-thiadiazole (1.0 g, 5.01 mmol, 1 eq), (2-methoxyphenyl)boronic acid (0.381 g, 2.51 mmol, 0.5 eq) and CsF (1.52 g, 10.0 mmol, 2 eq) in dioxane (20 mL) and water (5 mL) was degassed with Ar for 30 min. Pd(dppf)Cl_2_ (0.367 g, 0.501 mmol, 0.1 eq) was added and the reaction mixture was heated at 85 °C overnight. After cooling to room temperature, the reaction mixture was diluted with EtOAc and filtered. The phases of the filtrate were separated. The organic phase was concentrated under reduced pressure. The residue was purified by flash chromatography on silica gel using a gradient of EtOAc (0–20%) in hexane to afford 0.620 g (46%) of the title compound as a white solid. ^1^H NMR (400 MHz, DMSO-*d*_6_) δ 8.24 (d, *J* = 6.6 Hz, 1H), 7.69 (t, *J* = 7.2 Hz, 1H), 7.39 (d, *J* = 8.4 Hz, 1H), 7.22 (t, *J* = 7.6 Hz, 1H), 4.13 (s, 3H).

#### 3.1.31. 5-(2-Methoxyphenyl)-3-(pyridin-4-yl)-1,2,4-thiadiazole (**42**)

A mixture of **41** (0.550 g, 2.03 mmol, 1 eq), pyridin-4-ylboronic acid (0.374 g, 3.04 mmol, 1.5 eq) and K_2_CO_3_ (0.841 g, 6.09 mmol, 3 eq) in dioxane (20 mL) and water (5 mL) was degassed with Ar for 30 min. Pd(dppf)Cl_2_ (0.149 g, 0.203 mmol, 0.1 eq) was added and the reaction mixture was heated at 85 °C for 16 h. After cooling to room temperature, the reaction mixture was diluted with EtOAc and filtered. The phases of the filtrate were separated. The organic phase was concentrated under reduced pressure. The residue was purified by flash chromatography on silica gel using a gradient of EtOAc (0–40%) in hexane to afford 0.450 g (83%) of the title compound as a white solid. LC-MS (ESI, *m*/*z*): 270 [M + H]^+^. ^1^H NMR (400 MHz, DMSO-*d*_6_) δ 8.79 (d, *J* = 5.9 Hz, 2H), 8.47 (dd, *J* = 1.4, 7.8 Hz, 1H), 8.21 (d, *J* = 6.0 Hz, 2H), 7.68 (m, 1H), 7.40 (d, *J* = 8.2 Hz, 1H), 7.26 (t, *J* = 7.5 Hz, 1H), 4.16 (s, 3H).

#### 3.1.32. (3-(Pyridin-4-yl)-1,2,4-thiadiazol-5-yl)phenol (**43**)

To a solution of **42** (0.350 g, 1.30 mmol, 1 eq) in NMP (8 mL) were added LiBr (1.13 g, 13.0 mmol, 10 eq) and *p*TsOH (1.12 g, 6.50 mmol, 5 eq). The reaction mixture was heated at 150 °C for 1.5 h. After cooling to room temperature, the reaction mixture was diluted with water and extracted with EtOAc (3×). The organic phases were combined, washed with brine, dried over Na_2_SO_4_, filtered, and concentrated under reduced pressure. The residue was purified by flash chromatography on silica gel using a gradient of EtOAc (0–40%) in hexane to afford 0.180 g (54%) of the title compound as a white solid. LC-MS (ESI, *m*/*z*): 256 [M + H]^+^. ^1^H NMR (400 MHz, DMSO-*d*_6_) δ 11.99 (s, 1H), 8.79 (d, *J* = 4.6 Hz, 2H), 8.37 (d, *J* = 7.6 Hz, 1H), 8.22 (d, *J* = 4.4 Hz, 2H), 7.50 (t, *J* = 7.9 Hz, 1H), 7.15 (d, *J* = 8.4 Hz, 1H), 7.10 (m, *J* = 7.6 Hz, 1H).

#### 3.1.33. *tert*-Butyl 3-(2-(3-(pyridin-4-yl)-1,2,4-thiadiazol-5-yl)phenoxy)pyrrolidine-1-carboxylate (**44**)

To a solution of **43** (0.180 g, 0.705 mmol, 1 eq) in THF (5 mL) were added PPh_3_ (0.277 g, 1.06 mmol, 1.5 eq) and 40% DIAD in toluene (0.520 mL, 1.058 mmol, 1.5 eq). The reaction mixture was stirred at room temperature for 10 min. *tert*-Butyl 3-hydroxypyrrolidine-1-carboxylate (0.158 g, 0.846 mmol, 1.2 eq) was added and the reaction mixture was stirred at room temperature overnight. The reaction mixture was concentrated under reduced pressure. The residue was purified by flash chromatography on silica gel using a gradient of EtOAc (0–50%) in hexane to afford 0.105 g (35%) of the title compound as a white solid. LC-MS (ESI, *m*/*z*): 425 [M + H]^+^. ^1^H NMR (400 MHz, DMSO-*d*_6_) δ 8.79 (d, *J* = 5.7 Hz, 2H), 8.49 (d, *J* = 6.9 Hz, 1H), 8.20 (d, *J* = 5.9 Hz, 2H), 7.67 (t, *J* = 7.0 Hz, 1H), 7.43 (d, *J* = 8.4 Hz, 1H), 7.27 (t, *J* = 7.6 Hz, 1H), 5.49 (m, 1H), 3.76 (m, 1H), 3.66 (m, 1H), 3.48 (m, 2H), 2.26 (m, 2H), 1.41 (s, 9H).

#### 3.1.34. 3-(Pyridin-4-yl)-5-(2-(pyrrolidin-3-yloxy)phenyl)-1,2,4-thiadiazole dihydrochloride (**45**)

To a solution of **44** (0.105 g, 0.247 mmol, 1 eq) in dioxane (0.5 mL) cooled at 0 °C was added 4N HCl in dioxane (2.0 mL, 8.00 mmol, 32 eq). The reaction mixture was stirred at room temperature for 2 h. The reaction mixture was concentrated under reduced pressure. The residue was triturated with Et_2_O. The white solid was filtered and dried under high vacuum to afford quantitatively the title compound. LC-MS (ESI, *m*/*z*): 325 [M + H]^+^.

#### 3.1.35. 5-[2-(1-Cyclopentylpyrrolidin-3-yl)oxyphenyl]-3-(4-pyridyl)-1,2,4-thiadiazole (**46**)

To a solution of **45** (0.080 g, 0.223 mmol, 1 eq) in CH_2_Cl_2_ (1 mL) and MeOH (1 mL) was added cyclopentanone (0.049 mL, 0.557 mmol, 2.5 eq) and the reaction mixture was stirred at room temperature for 2 h. NaBH(OAc)_3_ (0.094 g, 0.446 mmol, 2 eq) was added portionwise and the reaction mixture was stirred at room temperature overnight. The reaction mixture was washed with water and the phases were separated. The aqueous phase was extracted with CH_2_Cl_2_ (3×). The organic phases were combined, dried over Na_2_SO_4_, filtered, and concentrated under reduced pressure. The residue was purified by reversed-phase HPLC (Xterra C18 column,250 × 19 mm, 10µ; 20 to 60% ACN in 20 mM ammonium bicarbonate in 20 min) to afford 0.008 g (9%) of the title compound as a white solid. LC-MS (ESI, *m*/*z*): 393 [M + H]^+^. ^1^H NMR (400 MHz, DMSO-*d*_6_) δ 8.80 (d, *J* = 5.9 Hz, 2H), 8.49 (d, *J* = 6.9 Hz, 1H), 8.22 (d, *J* = 5.9 Hz, 2H), 7.65 (t, *J* = 7.6 Hz, 1H), 7.33 (d, *J* = 8.3 Hz, 1H), 7.25 (t, *J* = 7.1 Hz, 1H), 5.33 (m, 1H), 2.76–3.12 (m, 4H), 2.30–2.61 (m, 2H), 2.01 (m., 1H), 1.78 (m, 2H), 1.65 (m, 2H), 1.37–1.58 (m, 4H) (Figure S39).

#### 3.1.36. 5-[2-(1-Cyclopentylpyrrolidin-3-yl)oxyphenyl]-3-(4-pyridyl)-1,2,4-thiadiazole (**49**)

To a solution 4-acetylpyridine oxime (0.200 g, 1.47 mmol, 1 eq) in THF (3 mL) cooled at −78 °C was added dropwise 2M LDA in hexane (2.2 mL, 4.40 mmol, 3 eq) over 30 min. A solution of **19** (0.458 g, 1.62 mmol, 1.1 eq) in THF (1 mL) was added dropwise and the reaction mixture was stirred at −78 °C for 2 h. The reaction was quenched by the addition of sat. NH_4_Cl. After warming to room temperature, the reaction mixture was extracted twice with EtOAc. The organic phases were combined, dried over Na_2_SO_4_, filtered and concentrated under reduced pressure to afford 5-(2-((1-cyclopentylpyrrolidin-3-yl)oxy)phenyl)-3-(pyridin-4-yl)-4,5-dihydroisoxazol-5-ol (48) which was dissolved in MeOH (3 mL). 12N HCl (3.0 mL, 36.0 mmol) was added and the reaction mixture was heated at 70 °C for 3 h. The reaction mixture was concentrated under reduced pressure. The residue was purified by reversed-phase HPLC (Xterra RP18 column,250 × 19 mm, 10µ; 30 to 90% MeOH in 20 mM ammonium bicarbonate in 24 min) to afford 0.035 g (12%) of the title compound as a beige solid. LC-MS (ESI, *m*/*z*): 393 [M + H]^+^. ^1^H NMR (400 MHz, DMSO-*d*_6_) δ 8.77 (d, *J* = 5.4 Hz, 2H), 7.93 (d, *J* = 7.8 Hz, 1H), 7.85 (d, *J* = 5.4 Hz, 2H), 7.47–7.57 (m, 2H), 7.22 (d, *J* = 8.3 Hz, 1H), 7.14 (t, *J* = 7.6 Hz, 1H), 5.13 (m, 1H), 2.75–2.99 (m, 3H), 2.47–2.57 (m, 1H), 2.41 (m, 1H), 2.31 (m, 1H), 1.91 (m, 1H), 1.77 (m, 2H), 1.61 (m, 2H), 1.34–1.55 (m, 4H) (Figure S40). ^13^C NMR (150 MHz, DMSO-*d*_6_) δ 167.09, 160.92, 154.25, 150.73, 136.04, 132.16, 127.33, 121.04, 120.92, 115.96, 114.42, 101.68, 77.59, 66.08, 59.28, 51.71, 31.80, 31.60, 31.51, 23.73 (Figure S41).

### 3.2. Antiviral Assays

#### 3.2.1. Cells and Viruses

The SARS-CoV-2 isolate used was derived from the BetaCov/Belgium/GHB-03021/2020 (EPI ISL407976|2020-02-03), which was isolated from a Belgian patient returning from Wuhan in February 2020. The isolate was passaged 7 times on VeroE6 cells which introduced two series of amino acid deletions in the spike protein [29]. The infectious content of the virus stock was determined by titration on Vero E6 cells.

The SARS-CoV-2 variants of concern (VoC) used in this study were Alpha B.1.1.7 (derived from hCoV-19/Belgium/rega-12211513/2020; EPI_ISL_791333, 2020-12-21) 17, and Delta B.1.617.2 (derived from hCoV-19/Belgium/rega-7214/2021; EPI_ISL_2425097; 2021-04-20). The variants were originally isolated in-house from nasopharyngeal swabs taken from travelers returning to Belgium (baseline surveillance) and were subjected to sequencing on a MinION platform (Oxford Nanopore, Oxford, UK) directly from the nasopharyngeal swabs [30]. Virus stocks were then grown on Vero E6 cells in (DMEM 2% FBS medium) and passaged two times. Median tissue culture infectious doses (TCID50) was defined by end-point titration as previously described [26].

The SARS-CoV-1 strain 200,300,592 (Vietnam) was obtained from the Centers for Disease Control and Prevention (CDC; Atlanta, GA, USA). MERS-CoV-Jordan-N3/2012 (Genbank accession nr KC776174) was obtained from the US Armed Forces Health Surveillance Center, Division of Global Emerging Infections Surveillance and Response System.

VeroE6 cells were maintained in Dulbecco’s Modified Eagle’s Medium (DMEM; Gibco cat no 41965-039) supplemented with heat-inactivated 10% *v*/*v* fetal calf serum (FCS; Biowest) and 500 µg/mL Geneticin (Gibco cat no 10131-0275) and kept under 5% CO_2_ at 37 °C.

#### 3.2.2. SARS-CoV-1 and SARS-CoV-2 Screening

The SARS-CoV-1 and SARS-CoV-2 antiviral assay in Vero E6 cells that was used in this study was derived from a previously established SARS-CoV-1 assay [21]. Stock solutions of the various compounds in DMSO (10 mM) were prepared. On day −1, the test compounds were serially diluted in assay medium and the plates were incubated overnight (37 °C, 5% CO_2_, and 95% relative humidity). After, VeroE6-eGFP cells were plated corresponding to a final density of 25,000 cells per well in black 96-well plates (Greiner Bio-One, Vilvoorde, Belgium; Catalog 655090). On day 0, the cells with compound were infected with SARS-CoV-1 (at 20 CCID_50_ per well) or SARS-CoV-2 (at 20 CCID_50_ per well). The plates were incubated in a humidified incubator at 37 °C and 5% CO_2_. At 4 days p.i., the wells were examined for eGFP expression using an argon laser-scanning microscope. The microscope settings were excitation at 488 nm and emission at 510 nm, and the fluorescence images of the wells were converted into signal values. The antiviral activity was expressed as EC_50_ defined as the concentration of compound achieving 50% inhibition of the virus-reduced eGFP signals as compared to the untreated virus-infected control cells.

The same methodology was applied for the VoC, in which cells were infected with the SARS-CoV-2 at a final MOI of approximately 0.05 TCID50/cell. The final dilution of the different strains was adapted in order to obtain a similar MOI between all variants of interest.

The cytotoxicity of the compounds for VeroE6 cells in the absence of virus was evaluated in a standard MTS assay as previously described [31].

MERS-CoV cytopathic effect reduction assays were performed in Huh-7 cells (1.5 × 104 cells/well) using 96-well plates. Cells, seeded on the day prior to infection, were incubated with 100 µL volumes of 2-fold serial dilutions of compounds in infection medium, followed by infection with 225 PFU of MERS-CoV in 50 µL, yielding a total assay volume of 150 µL. Non-infected cells were treated in parallel with the same dilution series of compounds to determine cytotoxicity. After incubation for 42 h at 37 °C, cell viability was quantified with the CellTiter-96 Aqueous Non-radioactive Cell Proliferation Kit (Promega, Madison, WI, USA) and absorption at 495 nm was measured with an EnVision multilabel plate reader (PerkinElmer) and after normalization to uninfected (and untreated cells), EC50 and CC50 values were determined by nonlinear regression using GraphPad Prism v8.0 (San Diego, CA, USA).

All SARS-CoV-1- and SARS-CoV-2-related work was conducted in the high-containment BSL3+ facilities of the KU Leuven Rega Institute (3CAPS) under licenses AMV 30112018 SBB 219 2018 0892 and AMV 23102017 SBB 219 2017 0589 according to institutional guidelines. Experiments with infectious MERS-CoV and SARS-CoV-1 were performed at the LUMC biosafety level 3 (BSL-3) facilities.

### 3.3. Pseudotyped Virus Neutralization Assay

Production of VSV pseudotyped with MERS-CoV spike [32] and SARS-CoV-2 spike [33] was previously described. Briefly, HEK-293T cells were transfected with pCAGGS expression vectors encoding MERS-CoV spike or SARS-CoV-2 spike carrying a 16 or 18 a.a. cytoplasmic tail truncation, respectively. On day one post transfection, cells were infected with the VSV-G pseudotyped VSVΔG bearing the firefly (*Photinus pyralis*) luciferase reporter gene. Twenty-four hours later, supernatants containing MERS-CoV spike, SARS-CoV spike, or SARS-CoV-2 spike pseudotyped VSV particles were harvested and titrated on African green monkey kidney Vero E6 (ATCC#CRL-1586) cells.

In the virus neutralization assay, compounds were threefold serially diluted at two times the desired final concentration in DMEM supplemented with 1% fetal bovine serum, 100 U/mL Penicillin, and 100 µg/mL Streptomycin (Lonza, Basel, Switzerland). Monoclonal antibodies against MERS-CoV spike (7.7G6) [32] or SARS2-CoV-2 spike (REGN10933) [34] were included as a positive control. Diluted compounds and mAbs were incubated with an equal volume of pseudotyped VSV particles for 1 h at room temperature, inoculated on confluent VeroE6 monolayers in a 96-well plate, and further incubated at 37 °C for 24 h. Cells were lysed with Luciferase Cell Culture Lysis 5× Reagent (Promega, Madison, Wisconsin, USA) at room temperature for 30 min. Luciferase activity was measured on a Promega GloMax^®^ Explorer luminometer using D-luciferin as a substrate (Promega, Madison, Wisconsin, USA). The half-maximal inhibitory concentrations (IC_50_) were determined using 4-parameter logistic regression (GraphPad Prism version 8, San Diego, CA, USA).

### 3.4. Nsp14 RapidFire MS Screening Assay

An endpoint 384-well plate assay was developed to assess nsp14 activity [35]. Briefly, enzyme and substrates, SAM (*S*-(5′-adenosyl)-l-methionine chloride hydrochloride [Cayman Chemical, Ann Arbor, MI, USA] and cap G(5′)ppp(5′)G sodium salt [New England Biolabs, Ipswich, MA, USA]) were incubated to allow the reaction to take place, and then the product was quantified using MS. Assays were performed in 384-well, clear, flat-bottom plates (Greiner 781101) to a final volume of 20 μL. Components were diluted in buffer (20 mM Tris, pH 8.0, 50 mM NaCl) containing 1 mM TCEP (Thermo Scientific, Waltham, MA, USA), 0.1 mg/mL bovine serum albumin, 0.005% Nonidet P40 (Roche), and 3 mM MgCl2. In the assay, 5 nM nsp14 was incubated with 1 μM SAM and 0.7 μM cap (FAC). Following a 60 min incubation, the reaction was quenched using 1% formic acid (VWR, Radnor, PA, USA) containing 0.03 μg/mL *S*-adenosylhomocysteine-d4 (d4SAH; Cambridge Bioscience, Cambridge, UK) and loaded on the RapidFire system by aspiration for 600 ms using the Agilent RapidFire 365 high-throughput system with integrated solid-phase extraction (SPE) interfaced with the Agilent 6740 triple quadrupole mass spectrometer. The sample was then automatically loaded onto a C18 Type C SPE cartridge (Agilent Technologies), and buffer salts and protein matrix were removed from the sample by washing the cartridge with the load solution (water containing 0.1% trifluoroacetic acid [TFA]) at a flow rate of 1.5 mL/min for 5000 ms. The retained and purified analytes were eluted from the cartridge with the elution solution (acetonitrile: water [9:1, *v*/*v*] containing 0.1% TFA) at 1.25 mL/min for 5000 ms and directed to the mass spectrometer. The cartridge was re-equilibrated with load solution at 1.5 mL/min for 500 ms. Both *S*-adenosylhomocysteine (SAH) and d4SAH were assessed using multiple selected reaction monitoring (MRM) transitions of 385.1/134 for SAH and 389.2/135.9 for d4SAH. The dwell time was 50 ms for each transition. The fragmentor voltage was set to 120 V for SAH and 100 for d4SAH, the collision energy to 12 V for SAH and 14 V for d4SAH, the cell accelerator voltage to 5 V, and the delta electron multiplier voltage to 200 V. The mass spectrometer was operated with a gas temperature of 350 °C, gas flow rate of 7 L/min, nebulizer pressure of 40 psi, and capillary voltage of 3000 V. The areas under the daughter ion peaks of SAH and d4SAH were integrated using RapidFire QQQ Quantitative Analysis software (Agilent Technologies), and the area ratios of the SAH to the internal standard d4SAH were used for quantitation.

### 3.5. SARS-CoV-2 Polymerase Assay

The compound concentrations leading to 50% inhibition of polymerase-mediated RNA synthesis was determined in IC_50_ buffer (50 mM HEPES pH 8.0, 10 mM KCl, 2 mM MnCl_2_, 2 mM MgCl_2_, 10 mM DTT) containing 350 nM of Poly(A) template, seven various concentrations of compound (from 1 to 100 µM) and 150 nM of nsp2 in complex with 450 nM of nsp7L8 and 450 nM of nsp8.

Reactions were conducted in 40 µL volume on a 96-well Nunc plate. All experiments were robotized by using a BioMek 4000 automate (Beckman). Two microliters of each compound diluted in 100% DMSO was added in wells to the chosen concentration (5% DMSO final concentration). For each assay, the enzyme mix was distributed in wells after a 5 min incubation at room temperature to form the active complex. Reactions were started by the addition of the UTP mix and were incubated at 30 °C for 20 min. Reaction assays were stopped by the addition of 20 µL EDTA 100 mM. Positive and negative controls consisted, respectively, of a reaction mix with 5% DMSO final concentration or EDTA 100 mM instead of compounds. Reaction mixes were then transferred to Greiner plate using a Biomek I5 automate (Beckman). Picogreen^®^ fluorescent reagent was diluted to 1/800° in TE buffer according to the manufacturer and 60 µL of reagent was distributed into each well of the Greiner plate. The plate was incubated for 5 min in the dark at room temperature and the fluorescence signal was then read at 480 nm (excitation) and 530 nm (emission) using a TecanSafire2.

IC_50_ was determined using the equation: % of active enzyme = 100/(1 + (I)2/IC_50_), where I is the concentration of inhibitor and 100% of activity is the fluorescence intensity without inhibitor. IC_50_ was determined from curve fitting using Prism software. For each value, results were obtained using triplicate in a single experiment. 3′dUTP was used as an inhibitor control.

### 3.6. Furin and TMPRSS2 Enzymatic Assays

Recombinant furin was purchased from BioLegend (#719406), human recombinant TRMPSS2 from Cliniscience (ref LS-G57269-100), and the fluorogenic DABSYL/Glu-TNSPRRAR↓SVAS-EDANS-labeled peptides encompassing the S1/S2 cleavage site were purchased from Genscript. Briefly, the reactions were performed at room temperature in black 384-well 553 polystyrene low-volume plates (CELLSTAR-Greiner Bio-One # 784476) at a final volume of 15 µL. The fluorescent peptides were used at 5 µM and the reactions were performed in 50 mM Tris buffer (pH 7.5), 0.2% Triton X-100, 1 mM CaCl_2_ in the presence of 2 nM of furin. The inhibitors solubilized in DMSO were tested at 50 µM with 5% DMSO final concentration in the enzymatic assay.

For the TMPRSS2 assay, the fluorescent peptides were used at 5 µM and the reactions were performed in 50 mM Tris buffer (pH 8), and 150 mM NaCl and TMPRSS2 were added at final concentrations of 50 nM.

The cleavage of the synthetic peptides was quantitated by determining the increase in EDANS (562 nM) fluorescence following the release of the DABCYL quencher. The EDANS was excited at 335 nM using a Safire 2 Tecan fluorimeter. Each reaction was performed in triplicate.

### 3.7. SARS-CoV-2 Main Protease (Mpro) Assay

SARS-CoV-2 main protease (Mpro) was recombinantly produced as described before [36]. Compounds were tested in a Förster resonance energy transfer (FRET) assay for inhibition of the SARS-CoV-2 Mpro. The peptide Dabcyl-KTSAVLQ↓SGFRKM-E(Edans)-NH2 (Biosyntan, Berlin, Germany) (↓ indicates the cleavage site), corresponding to the P7–P6′ residues of the nsp4–nsp5 processing site of the viral polyprotein pp1a/pp1ab, was used as the substrate. Quenching of the Edans fluorescence by the Dabcyl residue was eliminated after cleavage of the scissile bond between P1-Gln and P1′-Ser and the difference in fluorescence emission was measured with a Tecan Spark fluorescence plate reader, operated at an excitation wavelength of 360 nm and an emission wavelength of 460 nm.

Candidate compounds were kept in a DMSO stock solution; the Mpro was in a buffer containing 20 mM HEPES, 120 mM NaCl, 0.4 mM EDTA, 20% glycerol, pH 7.0. DTT (4 mM) was added to the buffer prior to running the assay. Following incubation of 50 nM SARS-CoV-2 Mpro with 100 µM of the candidate compound for 10 min at 37 °C, the reaction was initiated by the addition of 10 µM of the FRET substrate to each well. The final DMSO concentration was <2%.

## 4. Conclusions

A high-throughput screening of a drug-like compound library led to the discovery of a hit compound **1**, showing low micromolar activity against SARS-CoV-2 with a selectivity index of 5. Exploration of the SAR yielded compounds that were equally active, but showed a decreased cytotoxicity and hence displayed an improved SI. Hit compound 1 was profiled more extensively and was shown to be active against the VoCs of SARS-CoV-2, as well as against various betacoronaviruses. Furthermore, pseudovirus neutralization assays revealed that the compound exerts its antiviral effect via interference with viral entry. Overall, this study highlights the possibility to discover new hit compounds with promising activities against SARS-CoV-2 from a phenotypic approach. More investigations are, however, needed to improve the antiviral potency and to study its mechanism of action.

## Figures and Tables

**Table 1:** Antiviral activity and cytotoxicity of racemic **1** and its enantiomers **1a** and **1b**.

Compound		SARS-CoV-2 EC_50_ (µM)	MTS CC_50_ (µM)	SI (CC_50_/EC_50_)
**1**	Racemic	4.7 ± 3.0	21 ± 11	4.5
**1a**	(*R*)-enantiomer	3.6 ± 1.8	29 ± 24	8.1
**1b**	(*S*)-enantiomer	1.4 ± 0.1	18 ± 11	12.8

**Table 2:**
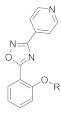
Antiviral activity and cytotoxicity of compounds **1**, **8**–**14**.

Compound	R	SARS-CoV-2 EC_50_ (µM)	MTS CC_50_ (µM)	SI (CC_50_/EC_50_)
**1**	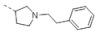	4.7 ± 3.0	21 ± 11	4.5
**8**	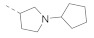	5.4 ± 3.5	41 ± 25	7.6
**9**	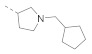	1.6 ± 0.8	24 ± 8	15
**10**	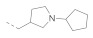	1.7 ± 0.3	27 ± 6	15.9
**11**	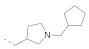	40 ± 14	>50	1.3
**12**	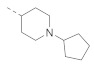	1.7 ± 0.5	46 ± 18	27.1
**13**	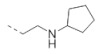	8.4 ± 2.5	46 ± 13	5.5
**14**	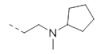	8.5 ± 4.0	37 ± 11	4.4

**Table 3:**
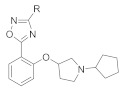
Antiviral activity and cytotoxicity of compounds **8** and **23**–**31**.

Compound	R	SARS-CoV-2 EC_50_ (µM)	MTS CC_50_ (µM)	SI (CC_50_/EC_50_)
**8**	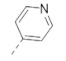	5.4 ± 3.5	41 ± 25	7.6
**23**	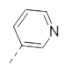	8.4 ± 5.7	>50	>6
**24**	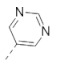	15 ± 2	>50	>3.3
**25**	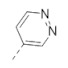	>50	>50	1
**26**	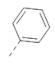	>50	9.0 ± 2.5	<1
**27**	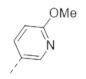	4.1 ± 2.2	20 ± 5.5	4.9
**28**	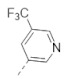	2.1 ± 0.1	10 ± 1	4.8
**29**	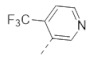	3.1 ± 1.3	26 ± 7	8.4
**30**	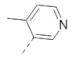	1.4 ± 1.0	47 ± 1	33.6
**31**	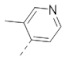	1.8 ± 0.3	18 ± 9	5.6

**Table 4:**
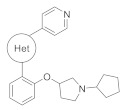
Antiviral activity and cytotoxicity of compounds **8**, **35**, **38**, **46**, and **49**.

Compound	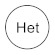	SARS-CoV-2 EC_50_ (µM)	MTS CC_50_ (µM)	SI (CC_50_/EC_50_)
**8**	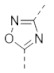	5.4 ± 3.5	41 ± 25	7.6
**35**	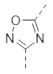	6.2 ± 2.7	30 ± 12	4.8
**38**	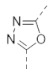	15 ± 3	47 ± 15	3.1
**46**	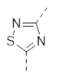	2.3 ± 0.4	15 ± 1	6.5
**49**	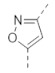	5.4 ± 2.1	14 ± 1	2.6

**Table 5:** Spectrum of antiviral activity of compound **1**.

SARS-CoV-2 ^a^ EC_50_ (µM)	B.1.1.7 ^a^ EC_50_ (µM)	B.1.617.2 ^a^ EC_50_ (µM)	SARS-CoV-1 ^a^ EC_50_ (µM)	VeroE6 CC_50_ (µM)	MERS ^b^ EC_50_ (µM)	Huh7 CC_50_ (µM)
4.7 ± 3.0	3.0 ± 1.0	4.0 ± 2.0	4.2 ± 0.2	21 ± 11	5.0	>20

^a^ Antiviral activity in VeroE6 cells in presence of P-gp inhibitor. ^b^ Antiviral activity in Huh-7 cells.

## Data Availability

Not applicable.

## Supplementary Materials

The following are available online, Figure S1: ^1^H NMR of compound **1** in DMSO-*d_6_*. Figure S2: ^13^C NMR of compound **1** in DMSO-*d*_6_. Figure S3: ^1^H NMR of compound **1a** in DMSO-*d*_6_. Figure S4: ^13^C NMR of compound **1a** in DMSO-*d*_6_. Figure S5: ^1^H NMR of compound **1b** in DMSO-*d*_6_. Figure S6: ^1^H NMR of compound **8** in DMSO-*d*_6_. Figure S7: ^1^H NMR of compound **9** in CD_3_OD. Figure S8: ^13^C NMR of compound **9** in DMSO-*d*_6_. Figure S9: ^1^H NMR of compound **10** in CD_3_OD. Figure S10: ^13^C NMR of compound **10** in DMSO-*d*_6_. Figure S11: ^1^H NMR of compound **11** in CD_3_OD. Figure S12: ^13^C NMR of compound **11** in DMSO-*d*_6_. Figure S13: ^1^H NMR of compound **12** in CD_3_OD. Figure S14: ^13^C NMR of compound **12** in DMSO-*d*_6_. Figure S15: ^1^H NMR of compound **13** in DMSO-*d*_6_. Figure S16: ^13^C NMR of compound **13** in DMSO-*d*_6_. Figure S17: ^1^H NMR of compound **14** in DMSO-*d*_6_. Figure S18: ^13^C NMR spectrum of compound **14** in DMSO-*d*_6_. Figure S19: ^1^H NMR spectrum of compound **23** in DMSO-*d*_6_. Figure S20: ^1^H NMR of compound **24** in CD_3_OD. Figure S21: ^13^C NMR of compound **24** in DMSO-*d*_6_. Figure S22: ^1^H NMR of compound **25** in DMSO-*d*_6_. Figure S23: ^13^C NMR spectrum of compound **25** in DMSO-*d*_6_. Figure S24: ^1^H NMR spectrum of compound **26** in DMSO-*d*_6_. Figure S25: ^13^C NMR of compound **26** in DMSO-*d*_6_. Figure S26: ^1^H NMR of compound **27** in DMSO-*d*_6_. Figure S27: ^1^H NMR of compound **28** in DMSO-*d*_6_. Figure S28: ^13^C NMR of compound **28** in DMSO-*d*_6_. Figure S29: ^1^H NMR of compound **29** in DMSO-*d*_6_. Figure S30: ^13^C NMR of compound **29** in DMSO-*d*_6_. Figure S31: ^1^H NMR of compound **30** in DMSO-*d*_6_. Figure S32: ^13^C NMR of compound **30** in DMSO-*d*_6_. Figure S33: ^1^H NMR of compound **31** in DMSO-*d*_6_. Figure S34: ^13^C NMR of compound **31** in DMSO-*d*_6_. Figure S35: ^1^H NMR of compound **35** in DMSO-*d*_6_. Figure S36: ^13^C NMR of compound **35** in DMSO-*d*_6_. Figure S37: ^1^H NMR of compound **38** in DMSO-*d*_6_. Figure S38: ^13^C NMR of compound **38** in DMSO-*d*_6_. Figure S39: ^1^H NMR of compound **46** in DMSO-*d*_6_. Figure S40: ^1^H NMR of compound **49** in DMSO-*d*_6_. Figure S41: ^13^C NMR of compound **49** in DMSO-*d*_6_.

## Sample Availability

Samples of the compounds are not available from the authors.
