# Supplementary material for: Synthesis, Structure–Activity Relationships, and Antiviral Profiling of 1-Heteroaryl-2-Alkoxyphenyl Analogs as Inhibitors of SARS-CoV-2 Replication
Source: Molecules. 2022 Feb 4;27(3):1052. doi: 10.3390/molecules27031052 (PMC8840742; doi:10.3390/molecules27031052)

Synthesis, Structure–Activity Relationships, and Antiviral Profiling of 1-Heteroaryl-2-Alkoxyphenyl Analogs As Inhibitors of SARS-CoV-2 Replication

Dorothée Bardiot [1,†], Laura Vangeel [2,†], Mohamed Koukni [1], Philippe Arzel [1], Marleen Zwaagstra [3], Heyrhyoung Lyoo [3], Patrick Wanningen [4], Shamshad Ahmad [5], Linlin Zhang [6], Xinyuanyuan Sun [6], Adrien Delpal [7], Cecilia Eydoux [7], Jean-Claude Guillemot [7], Eveline Lescrinier [8], Hugo Klaassen [1], Pieter Leyssen [2], Dirk Jochmans [2], Karolien Castermans [1], Rolf Hilgenfeld [6,9], Colin Robinson [5], Etienne Decroly [7], Bruno Canard [7], Eric J. Snijder [4], Martijn J. van Hemert [4], Frank van Kuppeveld [3], Patrick Chaltin [1,10], Johan Neyts [2], Steven De Jonghe [2,*] and Arnaud Marchand [1,*]

[1] Centre for Innovation and Stimulation of Drug Discovery (CISTIM), Gaston Geenslaan 2, B-3001 Leuven, Belgium; dorothee.bardiot@cistim.be (D.B.) ; mohamed.koukni@cistim.be (M.K.); philippe.arzel@cistim.be (P.A.); hugo.klaassen@cistim.be (H.K.); karolien.castermans@cistim.be (K.C.); patrick.chaltin@kuleuven.be (P.C.)

[2] Laboratory of Virology and Chemotherapy, KU Leuven, Department of Microbiology, Immunology and Transplantation, Rega Institute for Medical Research, Herestraat 49, P.O. Box 1043, 3000 Leuven, Belgium; laura.vangeel@kuleuven.be (L.V); Pieter.Leyssen@kuleuven.be (P.L.); dirk.jochmans@kuleuven.be (D.J.); johan.neyts@kuleuven.be (J.N.)

[3] Virology Section, Infectious Disease and Immunology Division, Department of Biomolecular Health Sciences, Faculty of Veterinary Medicine, Utrecht University, 3584 CL Utrecht, The Netherlands; m.zwaagstra@uu.nl (M.Z.); h.r.lyoo@uu.nl (H.L.); f.j.m.vankuppeveld@uu.nl (F.v.K.)

[4] Department of Medical Microbiology, Leiden University Medical Center, 2300 RC Leiden, The Netherlands; P.Wanningen@lumc.nl (P.W.); E.J.Snijder@lumc.nl (E.J.S.); M.J.van_Hemert@lumc.nl (M.J.v.H.)

[5] Drug Discovery Unit, School of Life Sciences, University of Dundee, Dundee DDI 5EH, UK; s.a.ahmad@dundee.ac.uk (S.A.); c.x.robinson@dundee.ac.uk (C.R.)

[6] Institute of Molecular Medicine, University of Lübeck, 23562 Lübeck, Germany; llzhang@biochem.uni-luebeck.de (L.Z.); xinyuanyuan.sun@uni-luebeck.de (X.S.); rolf.hilgenfeld@uni-luebeck.de (R.H.)

[7] Laboratory Architecture et Fonction des Macromolécules Biologiques (AFMB),UMR 7257, Aix Marseille University, Centre National de la Recherche Scientifique (CNRS) CEDEX 9, 13288 Marseille, France; adrien.delpal@univ-amu.fr (A.D.); cecilia.eydoux@univ-amu.fr (C.E.); jean-claude.guillemot@univ-amu.fr (J.-C.G.); etienne.decroly@univ-amu.fr (E.D.); bruno.canard@univ-amu.fr (B.C.)

[8] Medicinal Chemistry, Rega Institute for Medical Research, KU Leuven, Herestraat 49, box 1041, 3000 Leuven, Belgium; eveline.lescrinier@kuleuven.be (E.L.)

[9] German Center for Infection Research (DZIF), Hamburg-Lübeck-Borstel-Riems Site, University of Lübeck, 23562 Lübeck, Germany

[10] Center for Drug Design and Development (CD3), KU Leuven R&D, Waaistraat 6, B-3000 Leuven, Belgium

* Correspondence: steven.dejonghe@kuleuven.be (S.D.J.); arnaud.marchand@cistim.be (A.M.)

† These authors contributed equally to this work.

^{1}H and ^{13}C NMR spectra of final compounds

Figure S1. ^{1}H NMR spectrum of compound **1** in DMSO-d_6

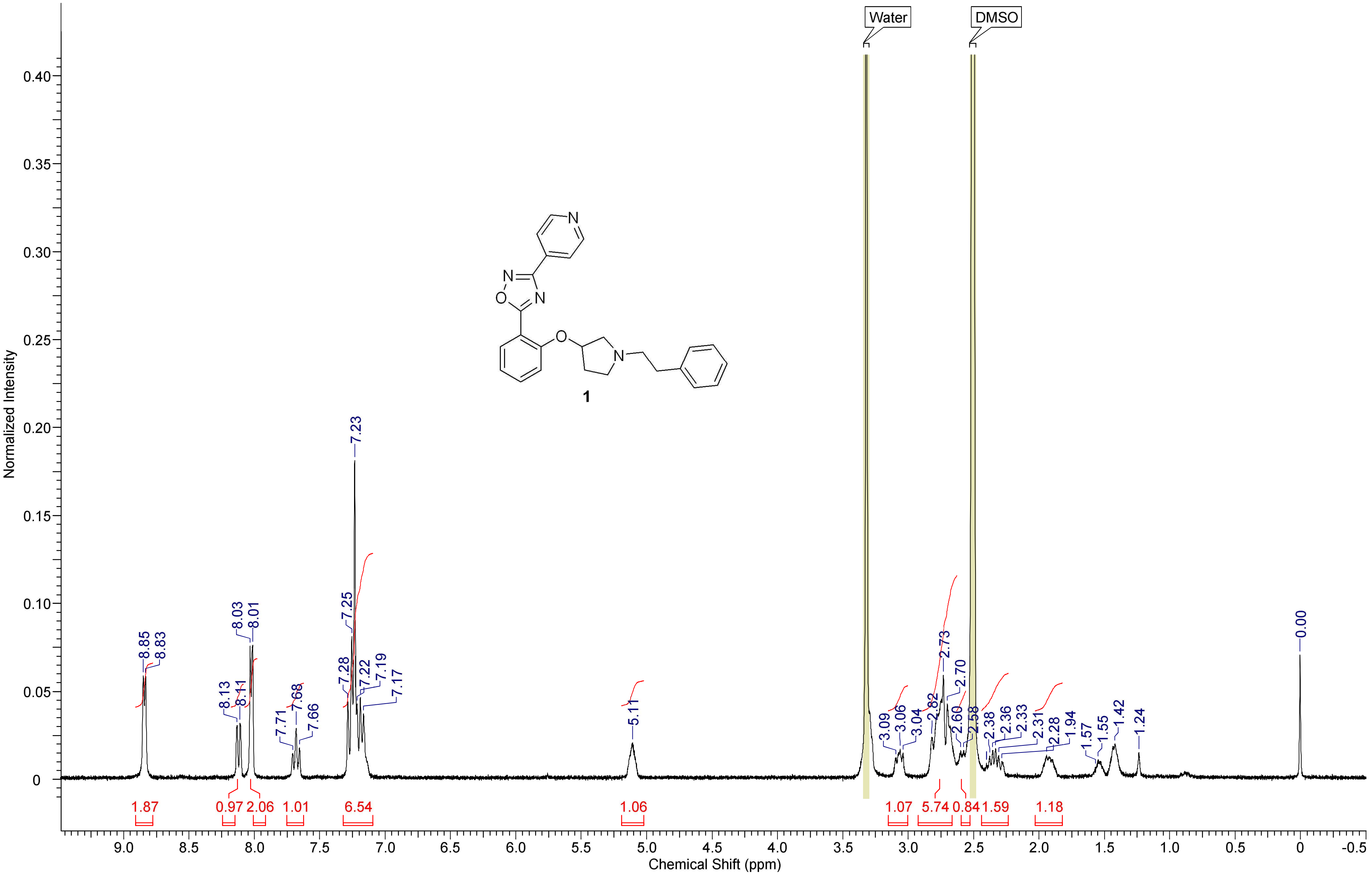

Figure S2. ^{13}C NMR of compound **1** in DMSO-d_6

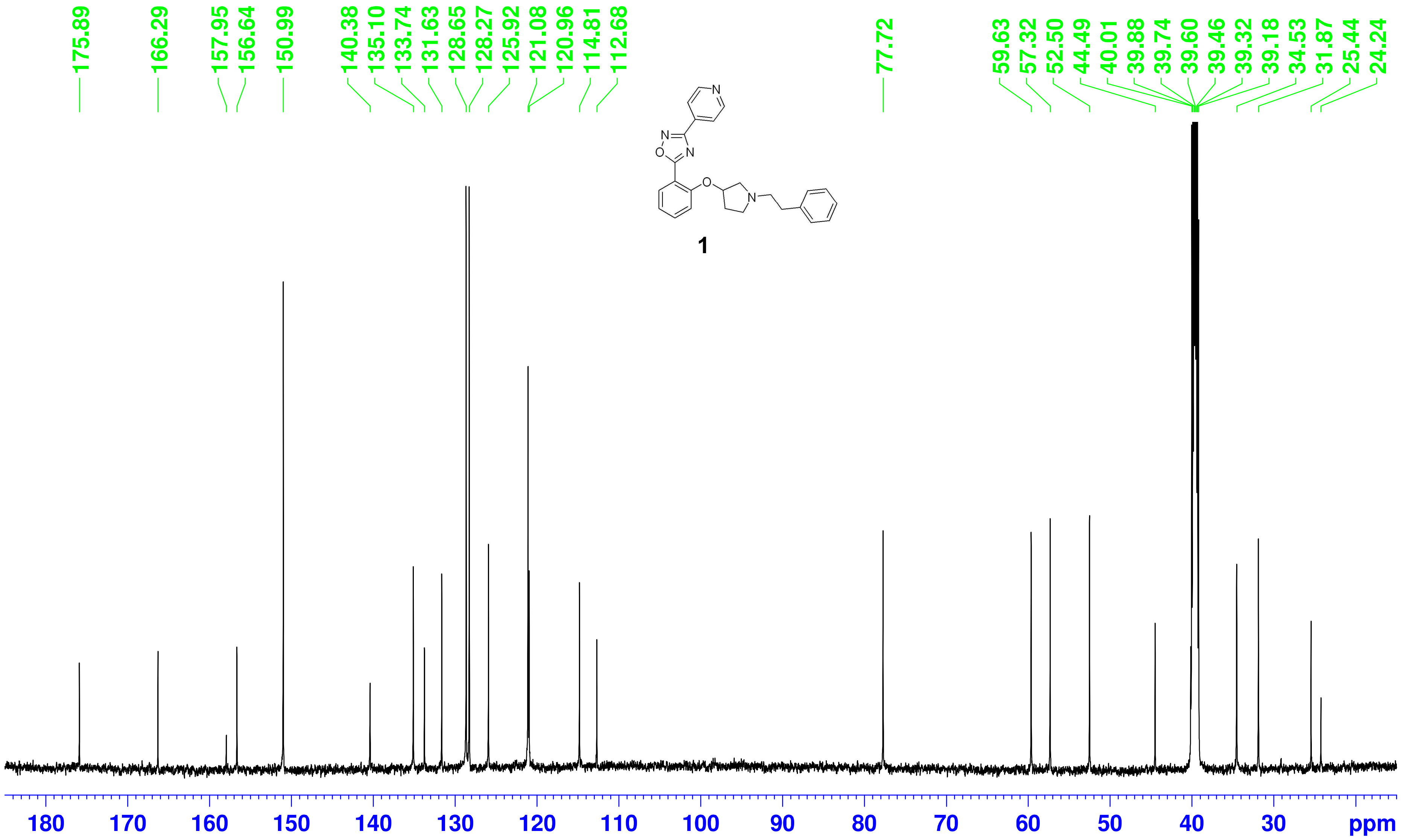

Figure S3. ^{1}H NMR of compound **1a** in DMSO-d_6

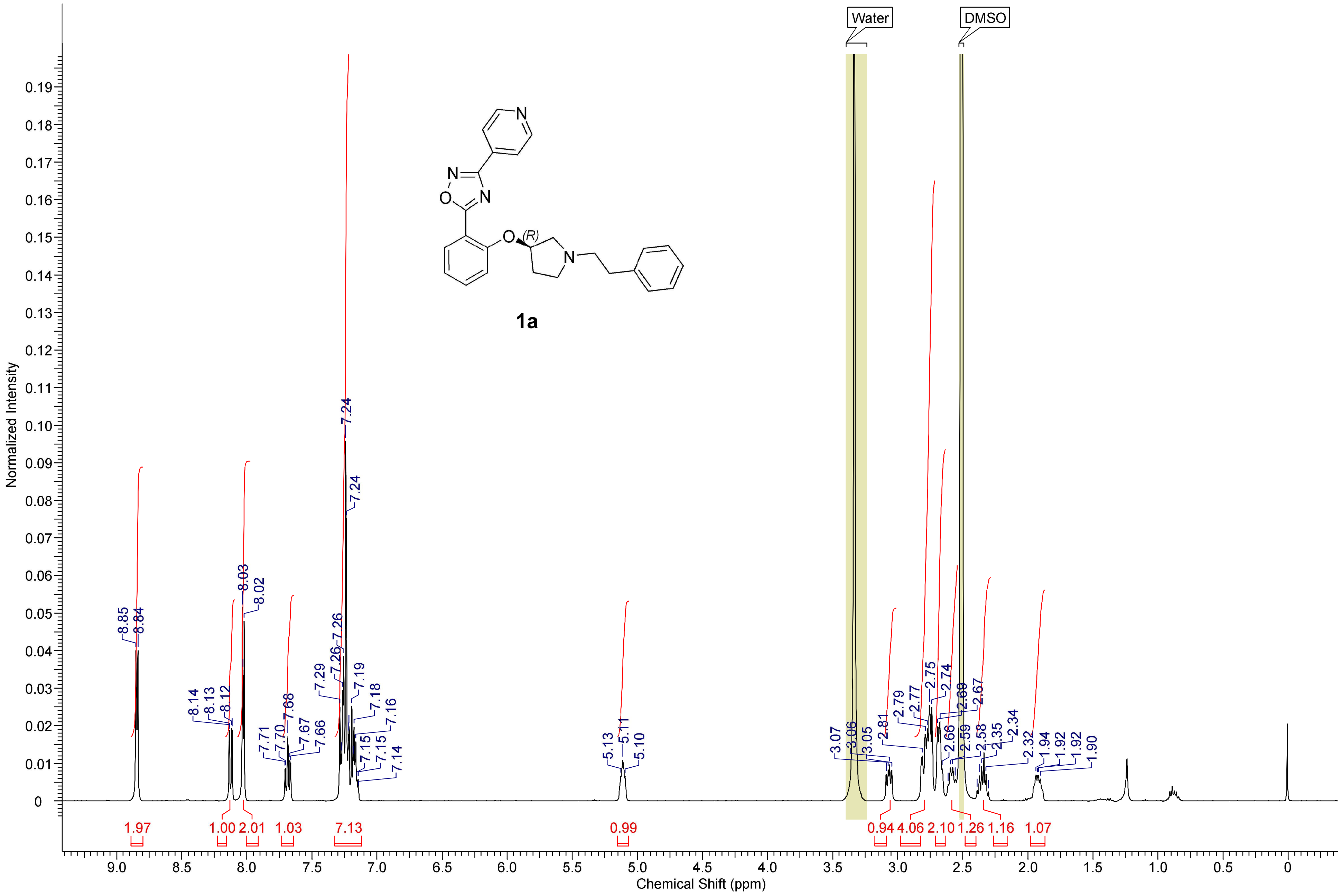

Figure S4. ^{13}C NMR of compound **1a** in DMSO-d_6

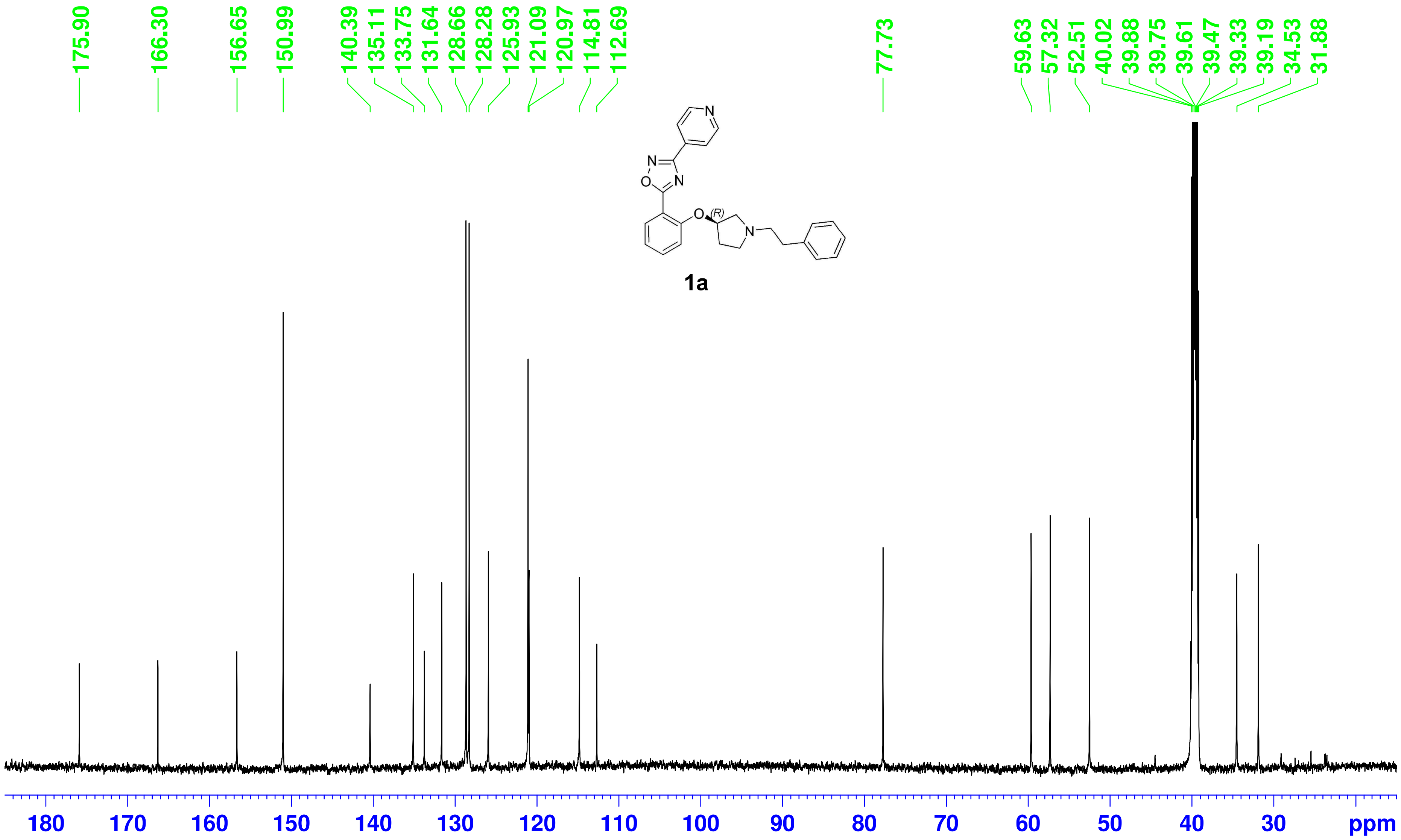

Figure S5. ¹H NMR of compound **1b** in DMSO-d_6

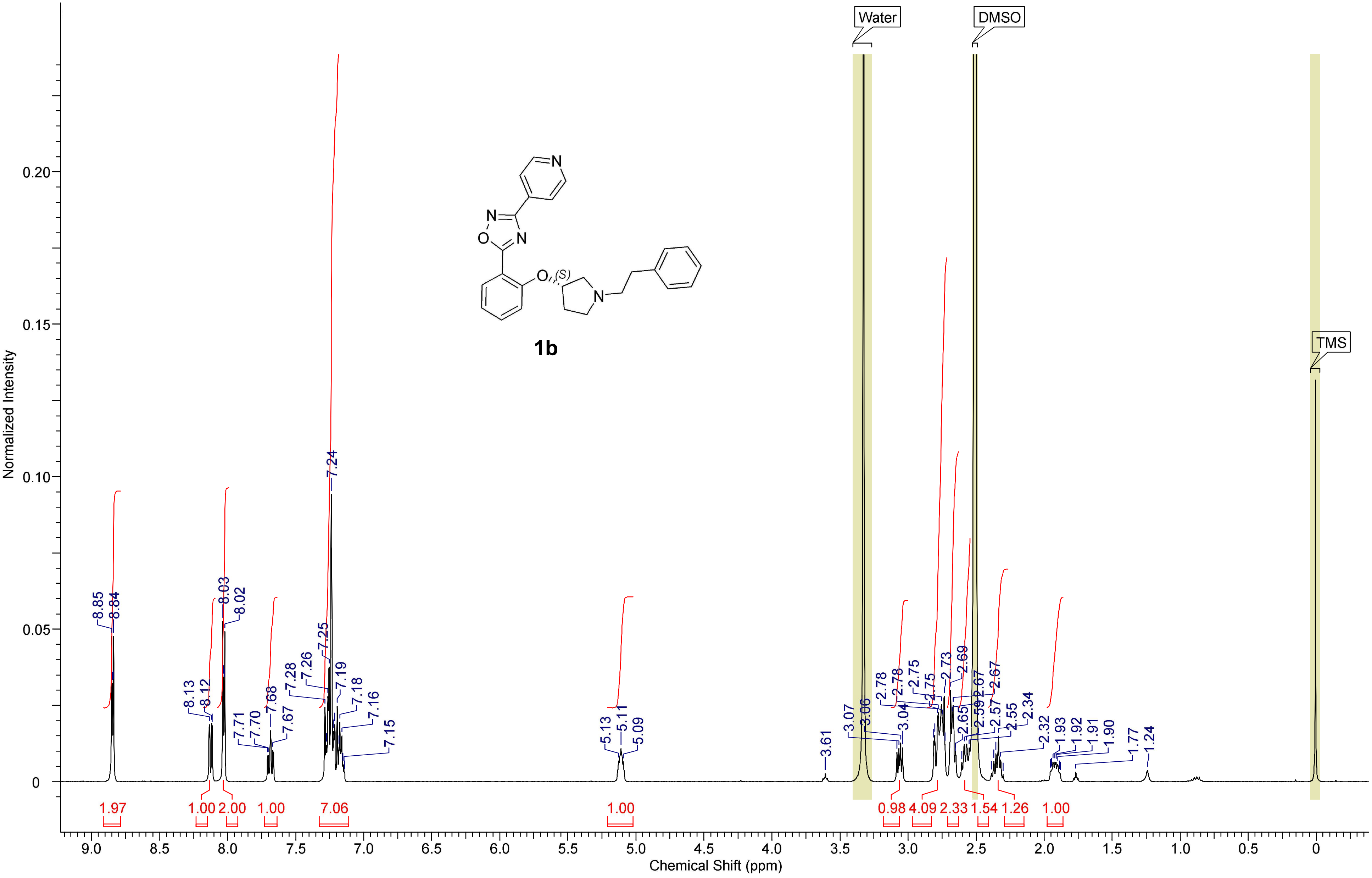

Figure S6. ^{1}H NMR of compound **8** in DMSO-d_6

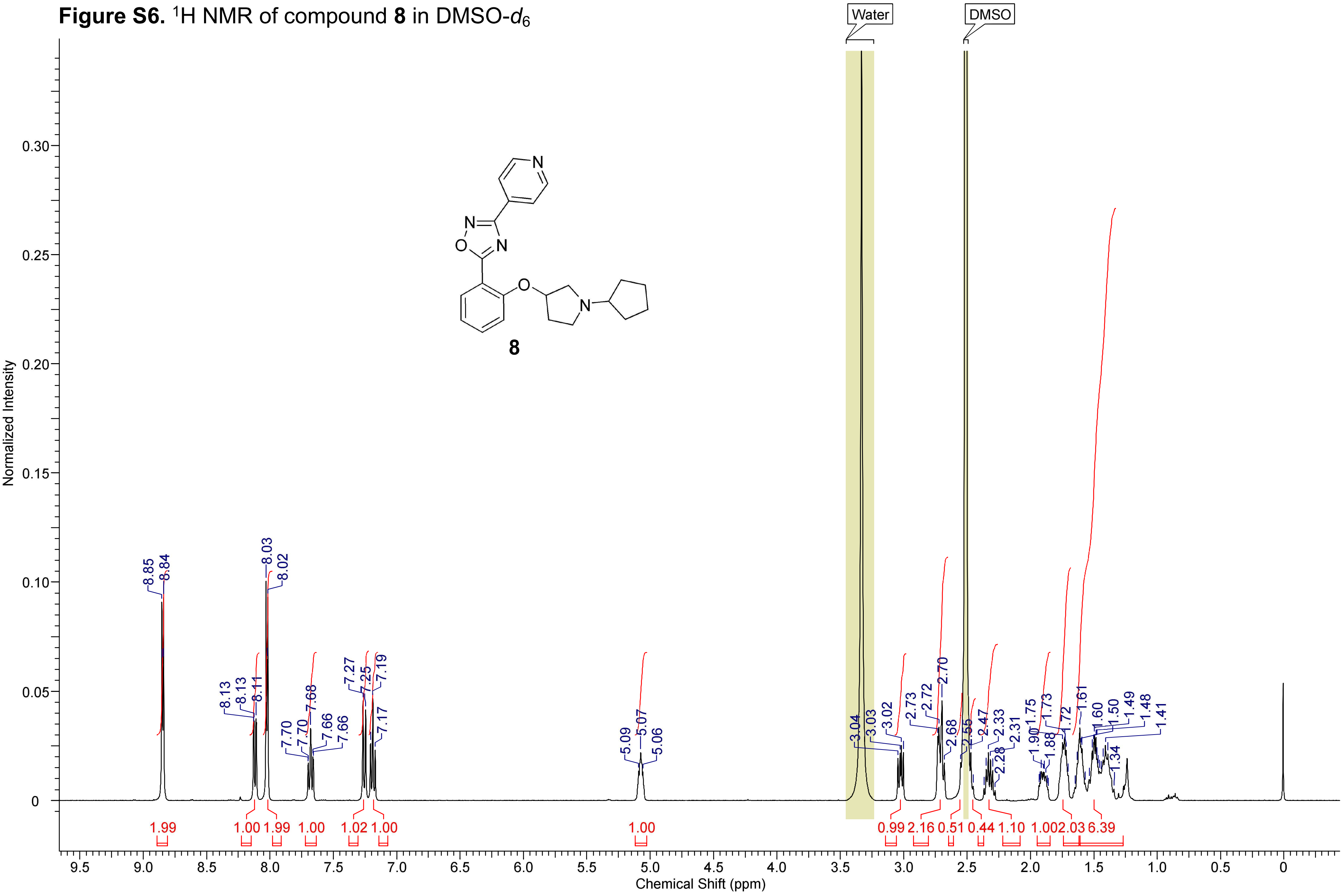

Figure S7. ^{1}H NMR of compound **9** in CD_3OD

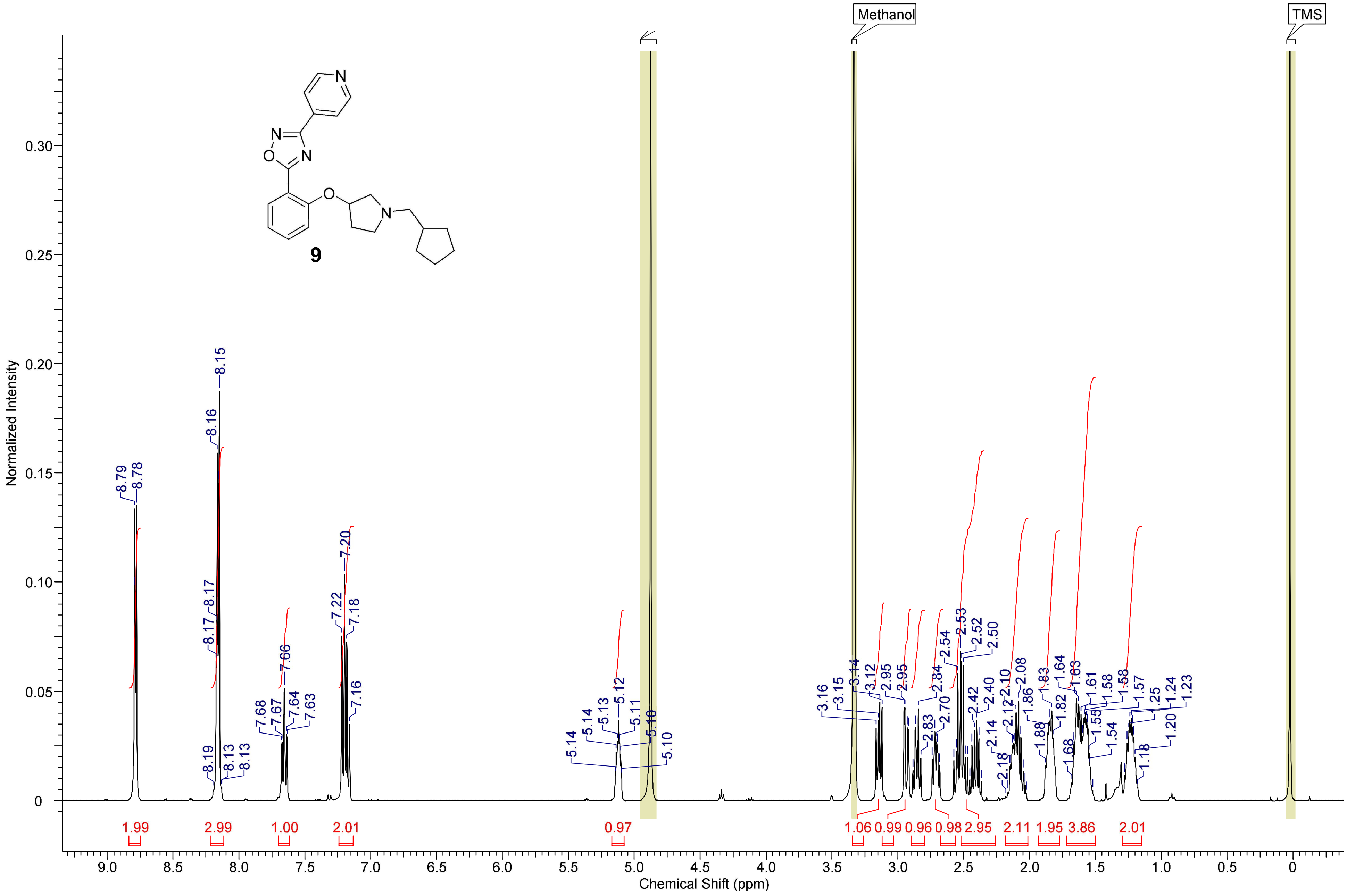

Figure S8. ^{13}C NMR of compound **9** in DMSO-d_6

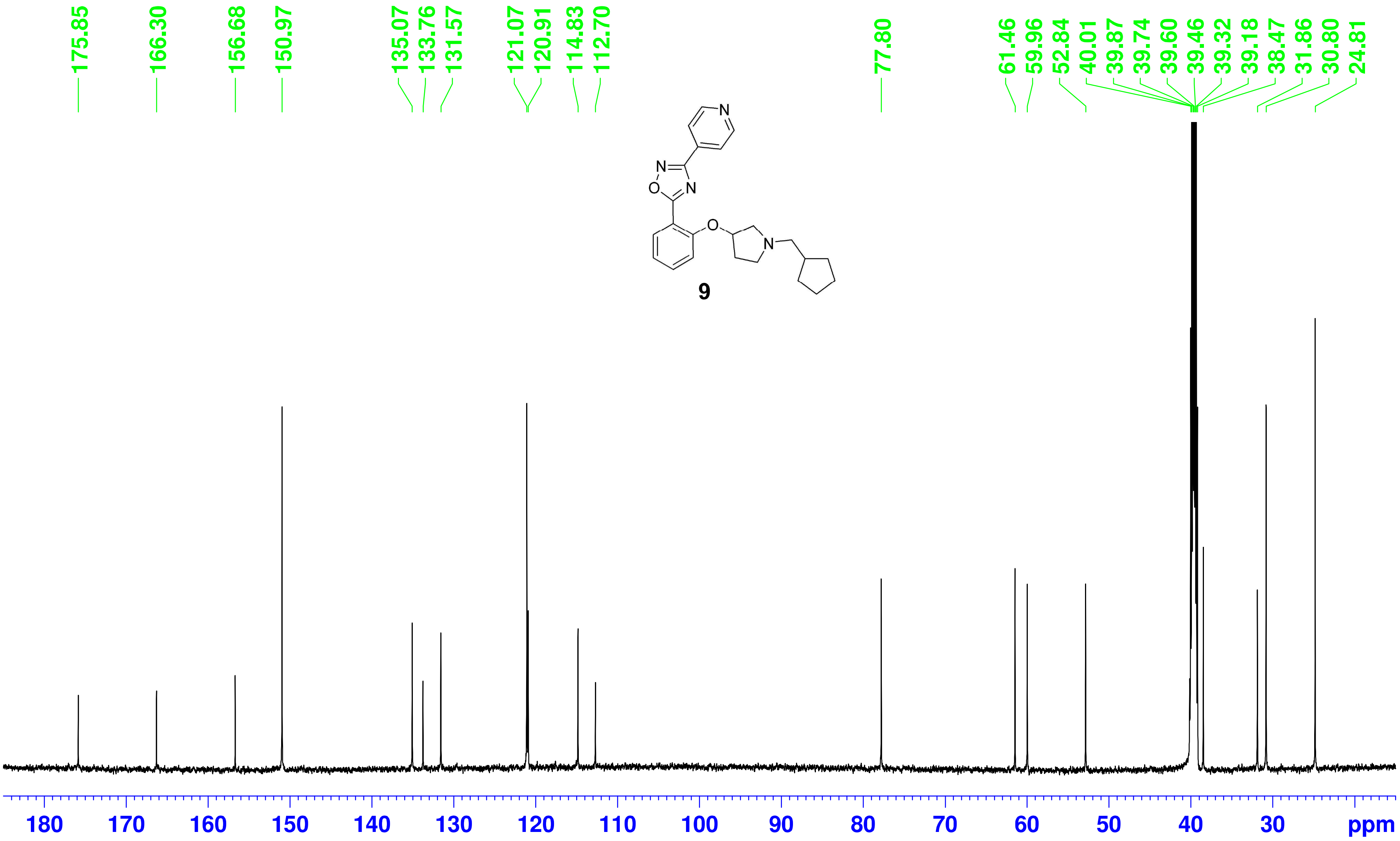

Figure S9. ^{1}H NMR of compound **10** in CD_3OD

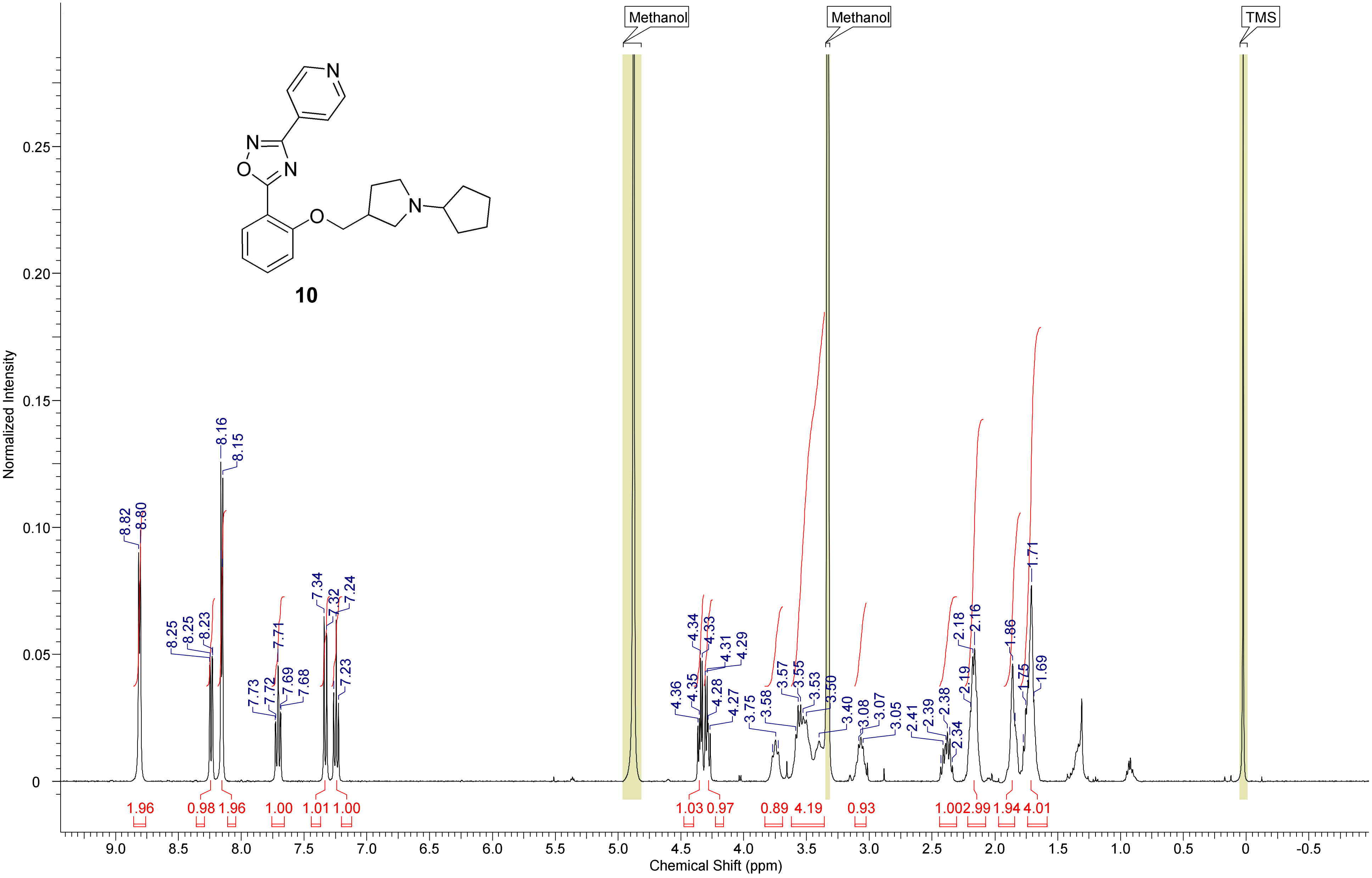

Figure S10. ^{13}C NMR of compound **10** in DMSO-d_6

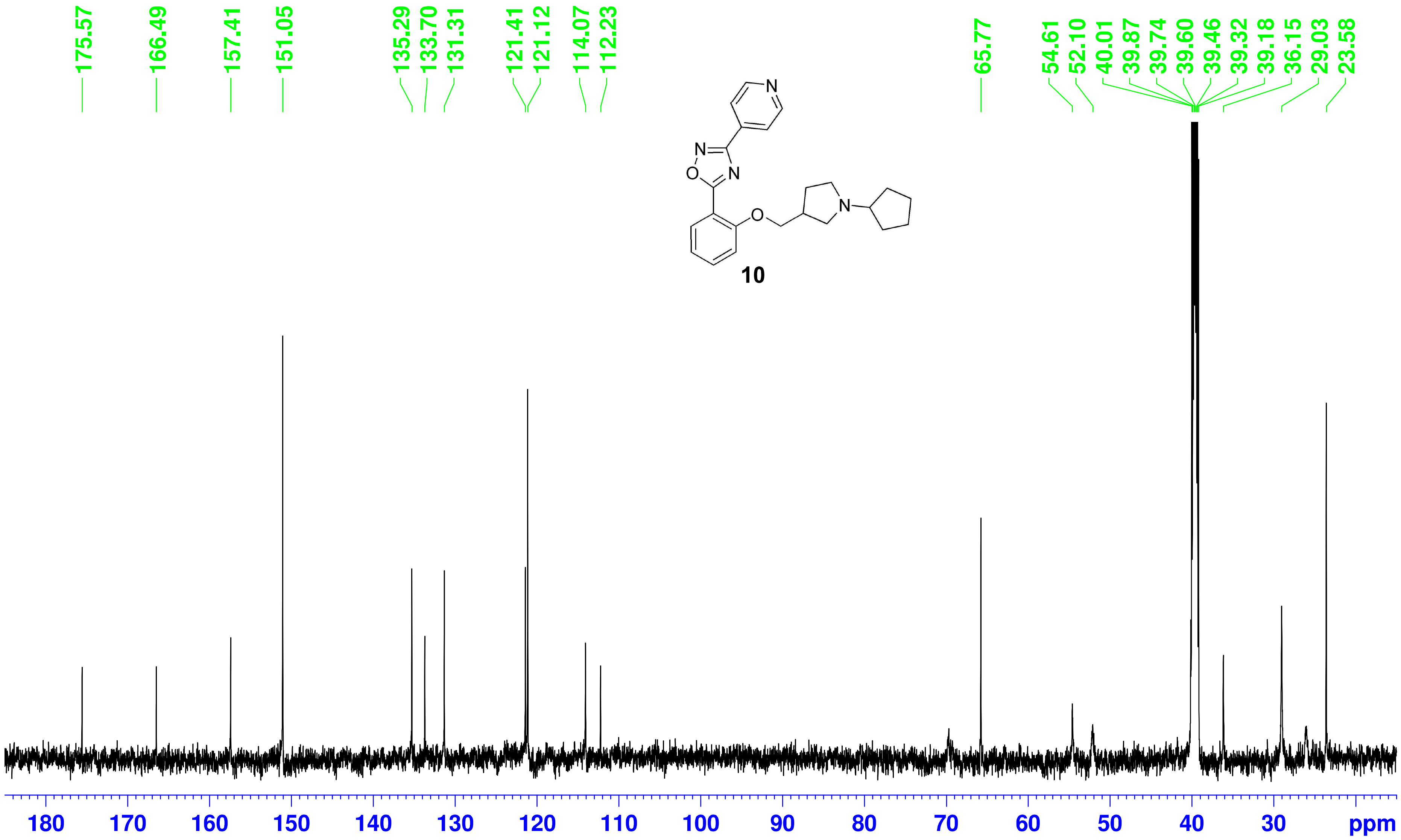

Figure S11. ^{1}H NMR of compound **11** in CD_3OD

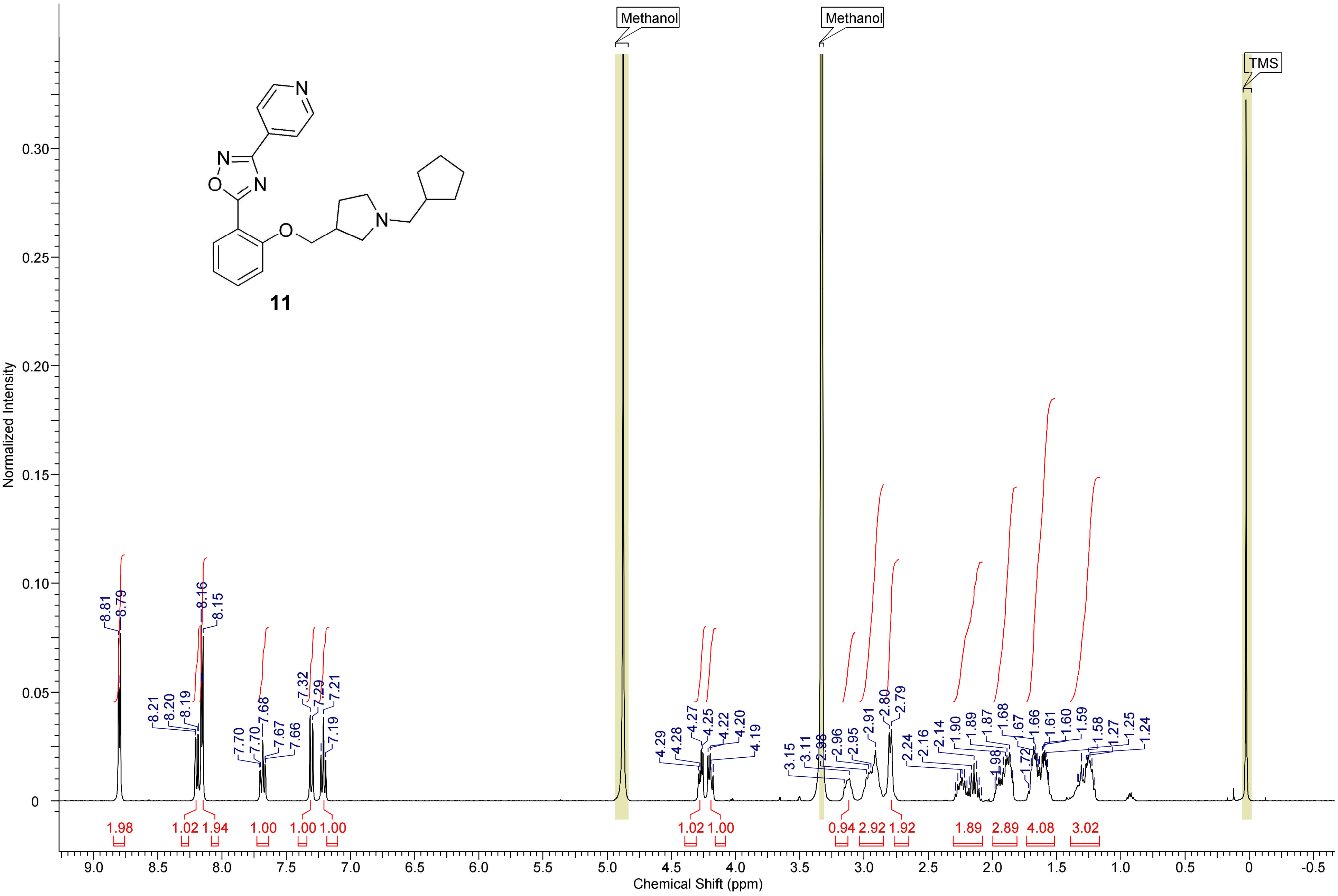

Figure S12. ^{13}C NMR of compound **11** in DMSO-d_6

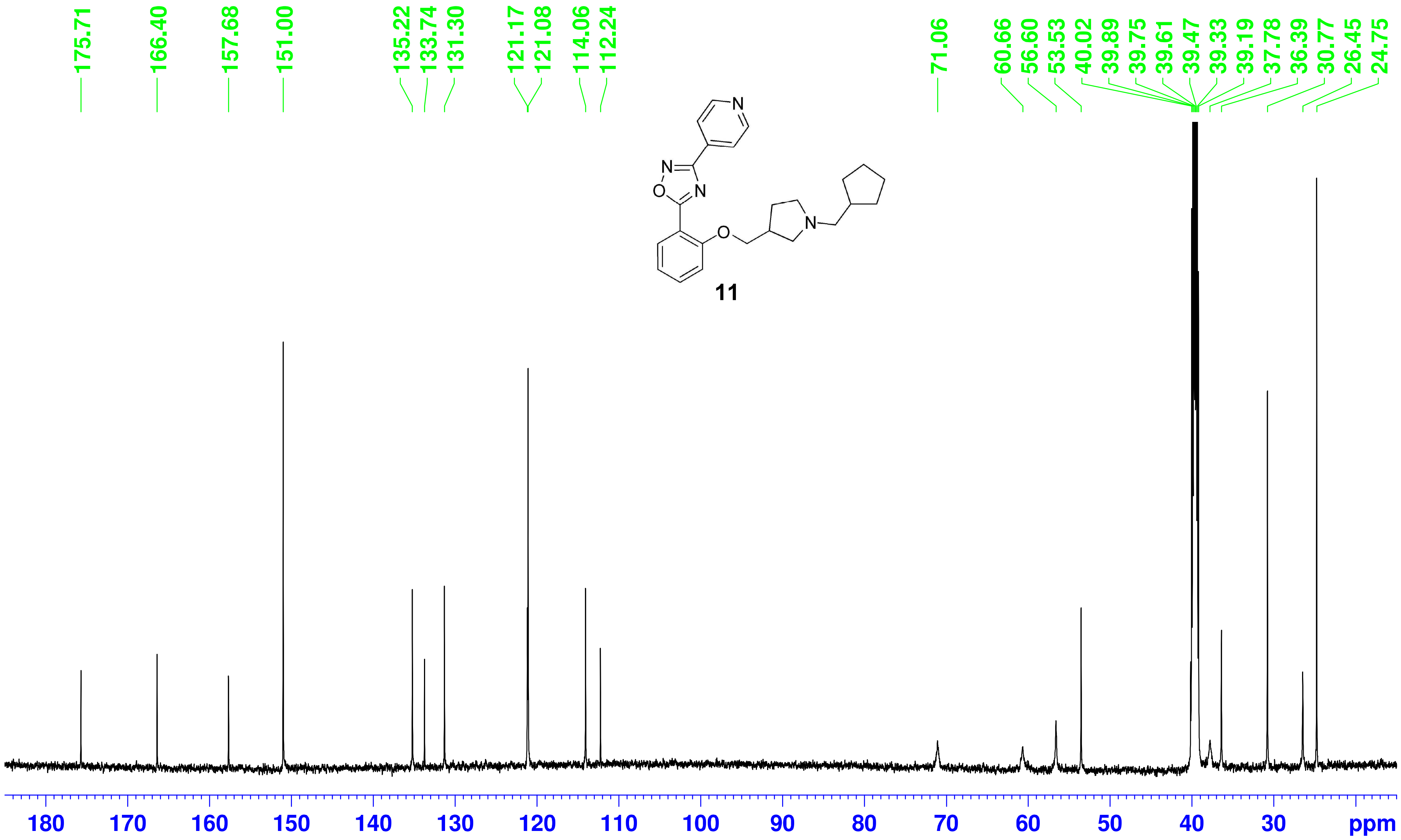

Figure S13. ^{1}H NMR of compound **12** in CD_3OD

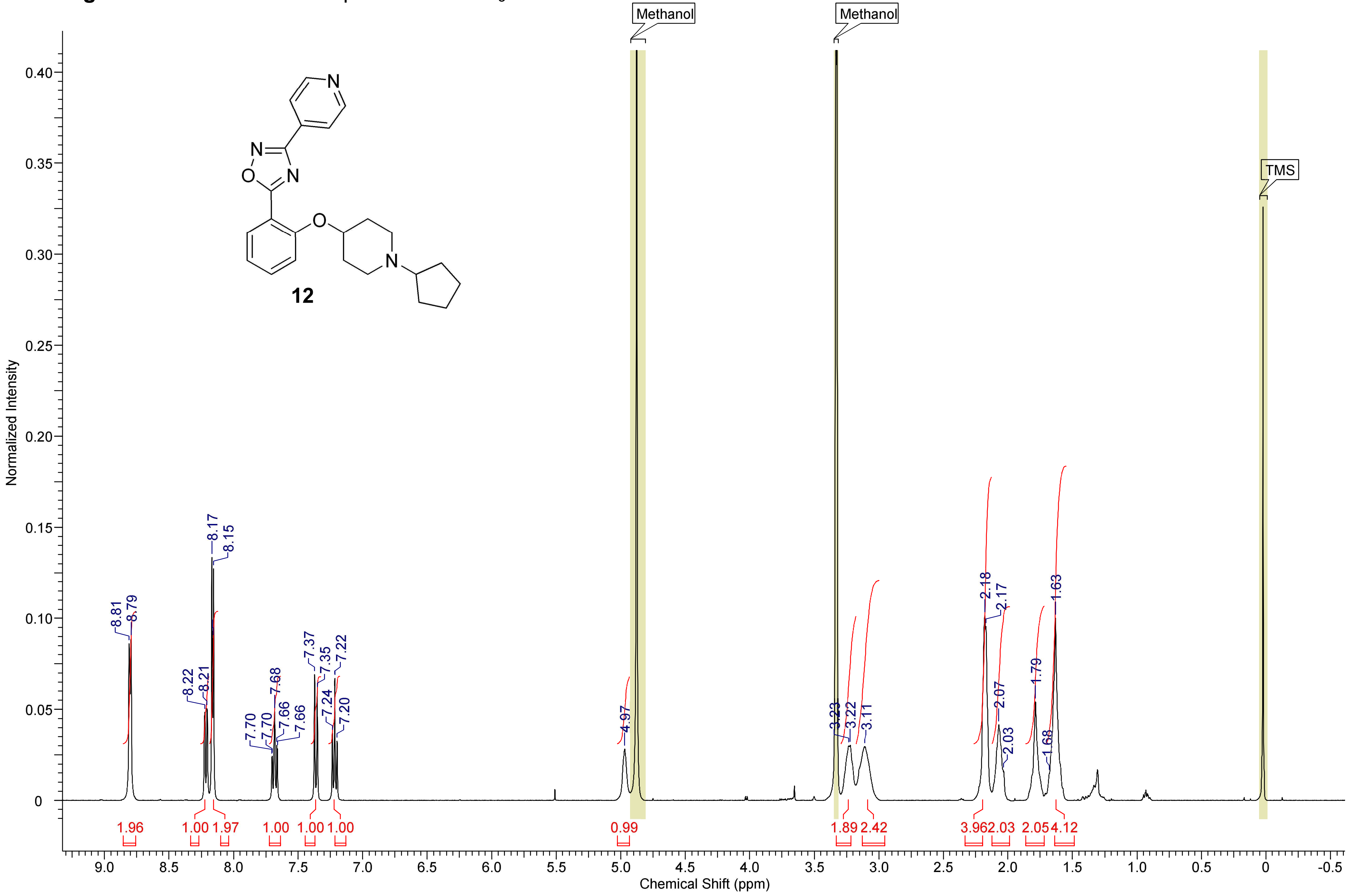

Figure S14. ^{13}C NMR of compound **12** in DMSO-d_6

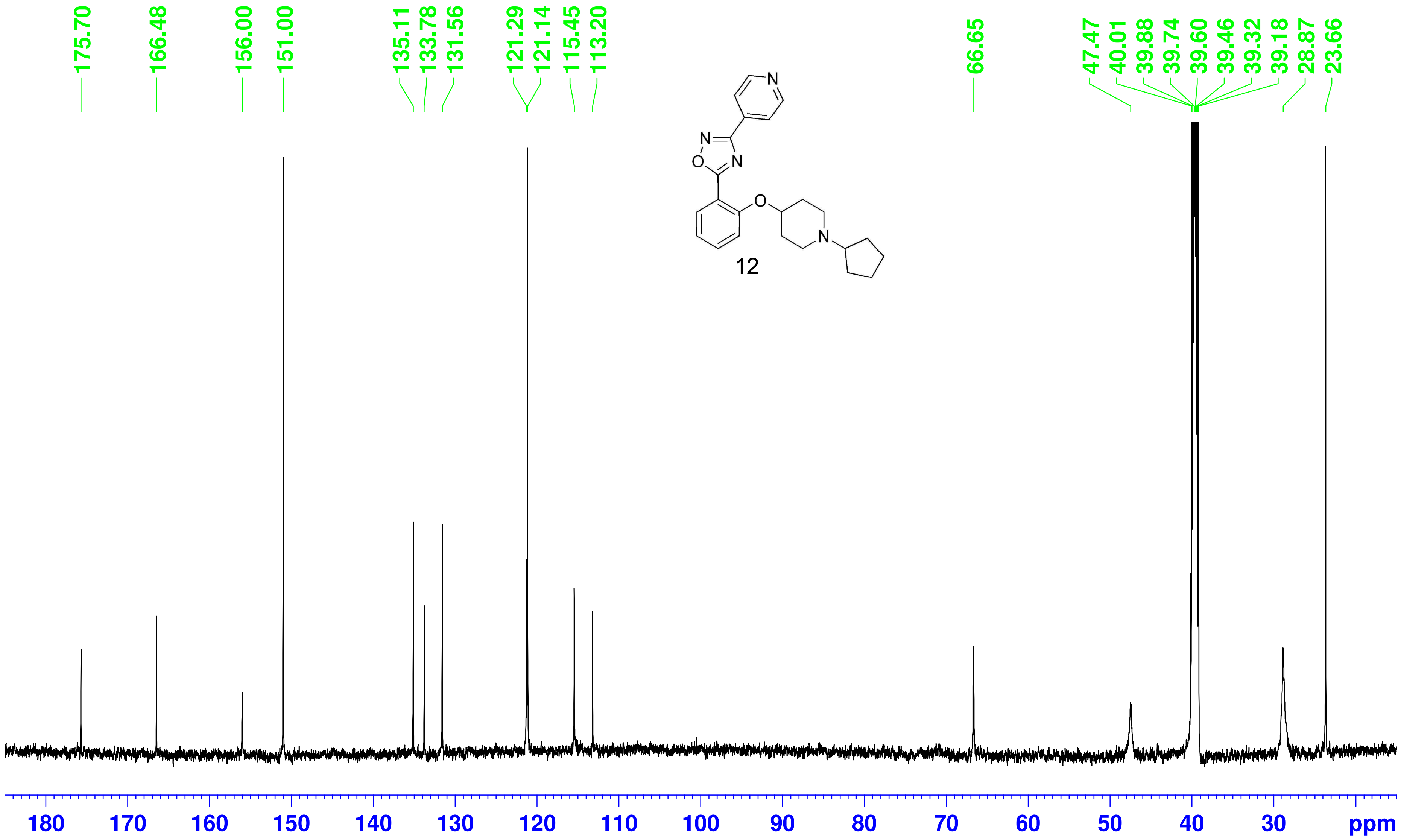

Figure S15. ^{1}H NMR of compound **13** in DMSO-d_6

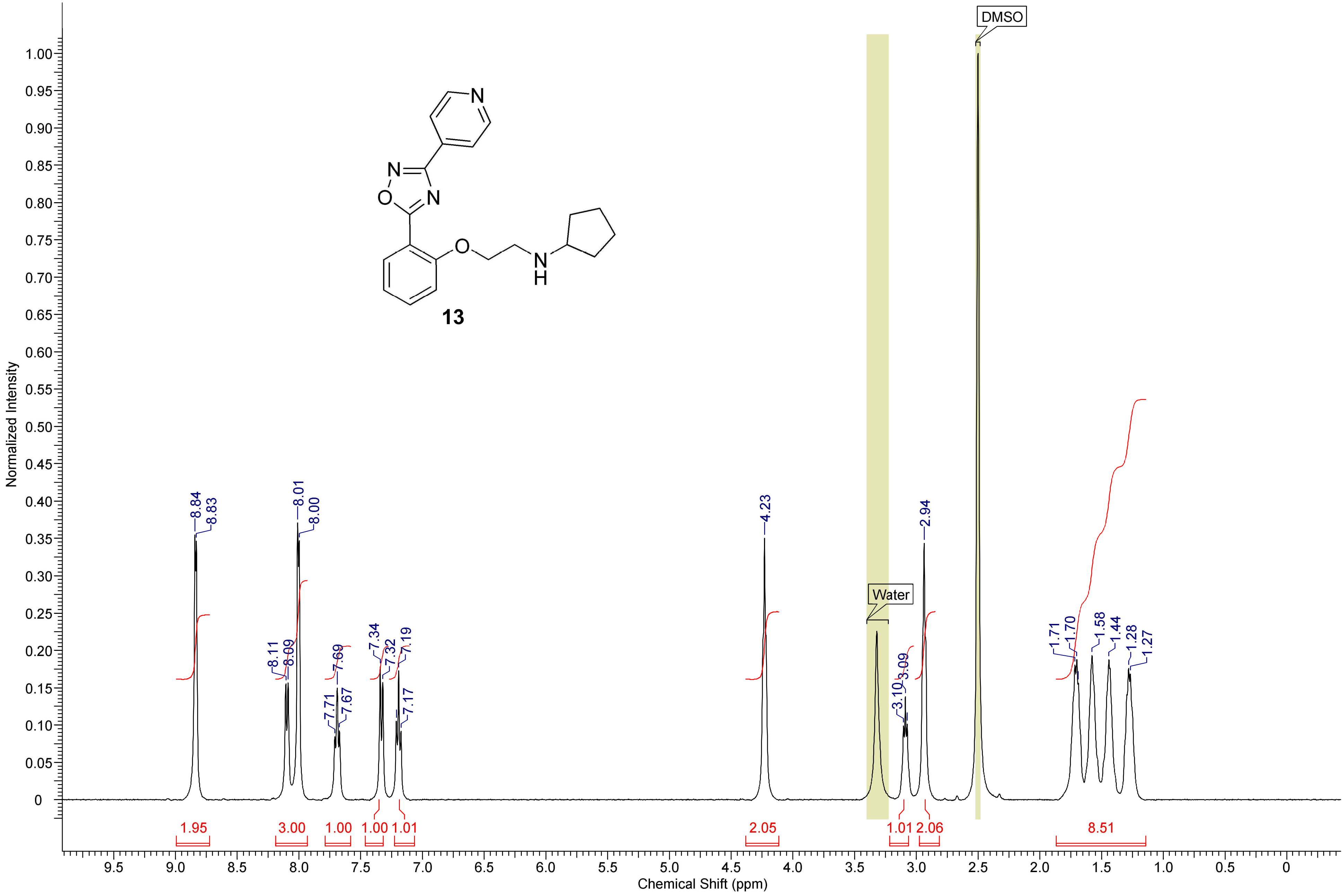

Figure S16. ^{13}C NMR of compound **13** in DMSO-d_6

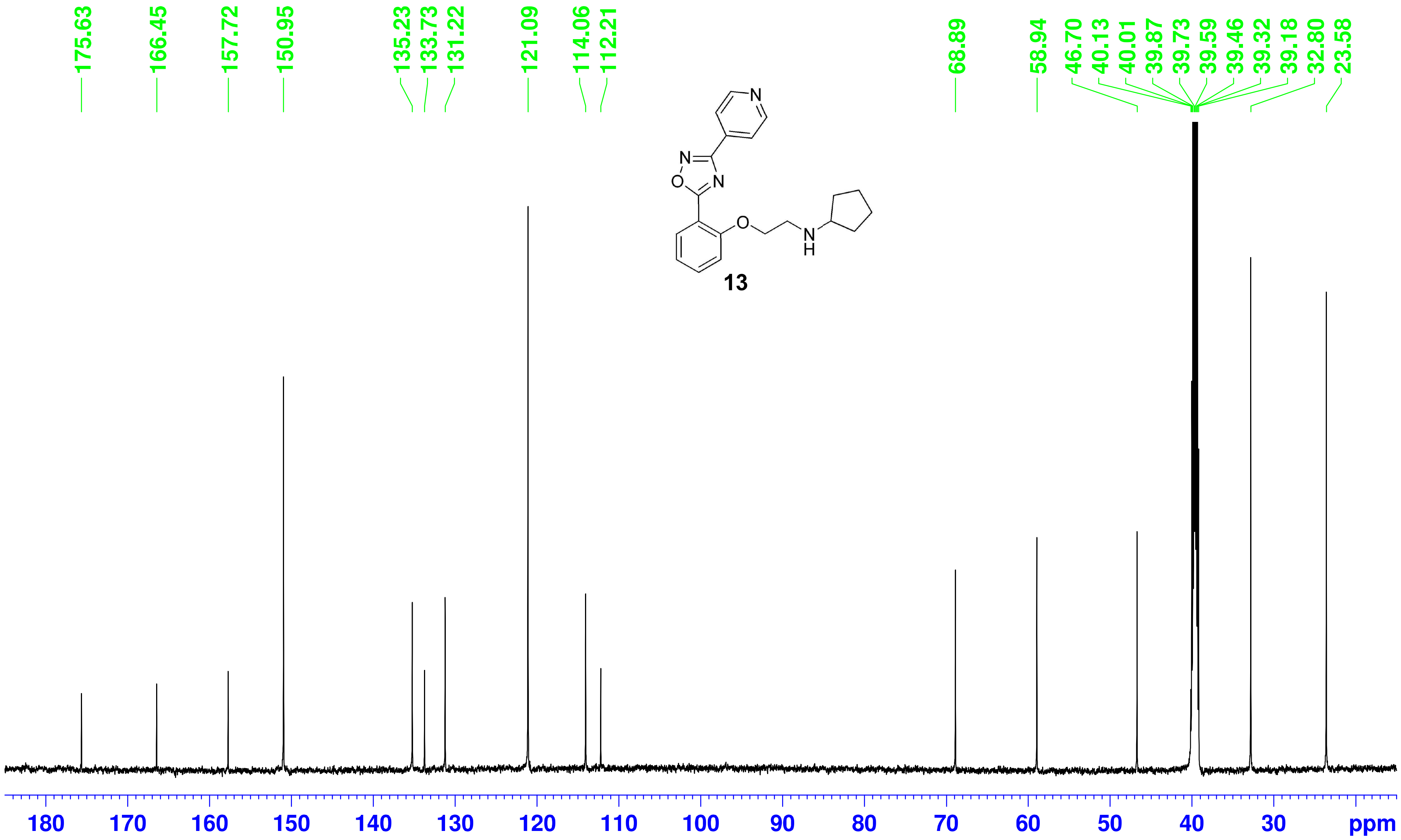

Figure S17. ^{1}H NMR of compound **14** in DMSO-d_6

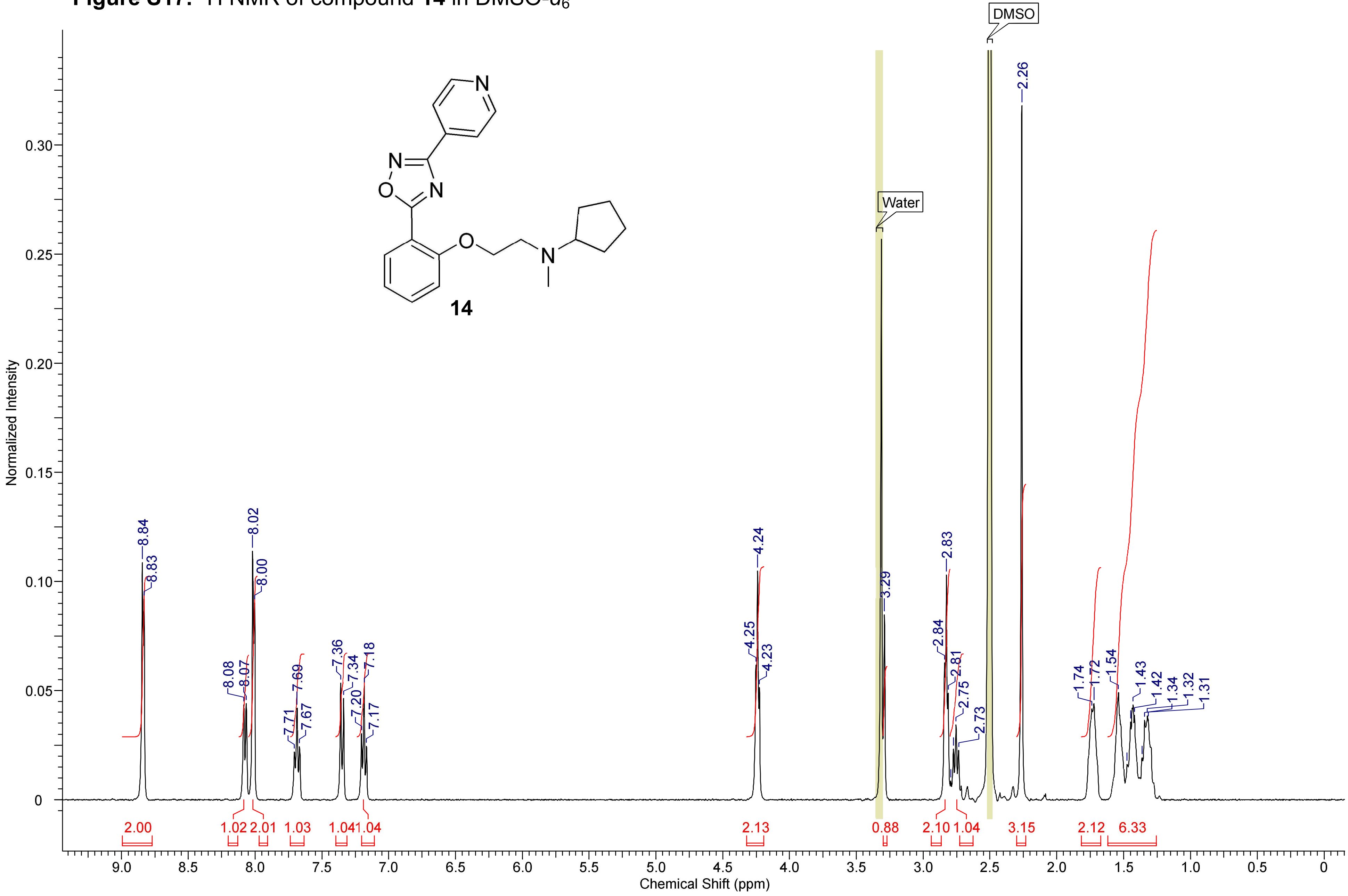

Figure S18. ^{13}C NMR of compound **14** in DMSO-d_6

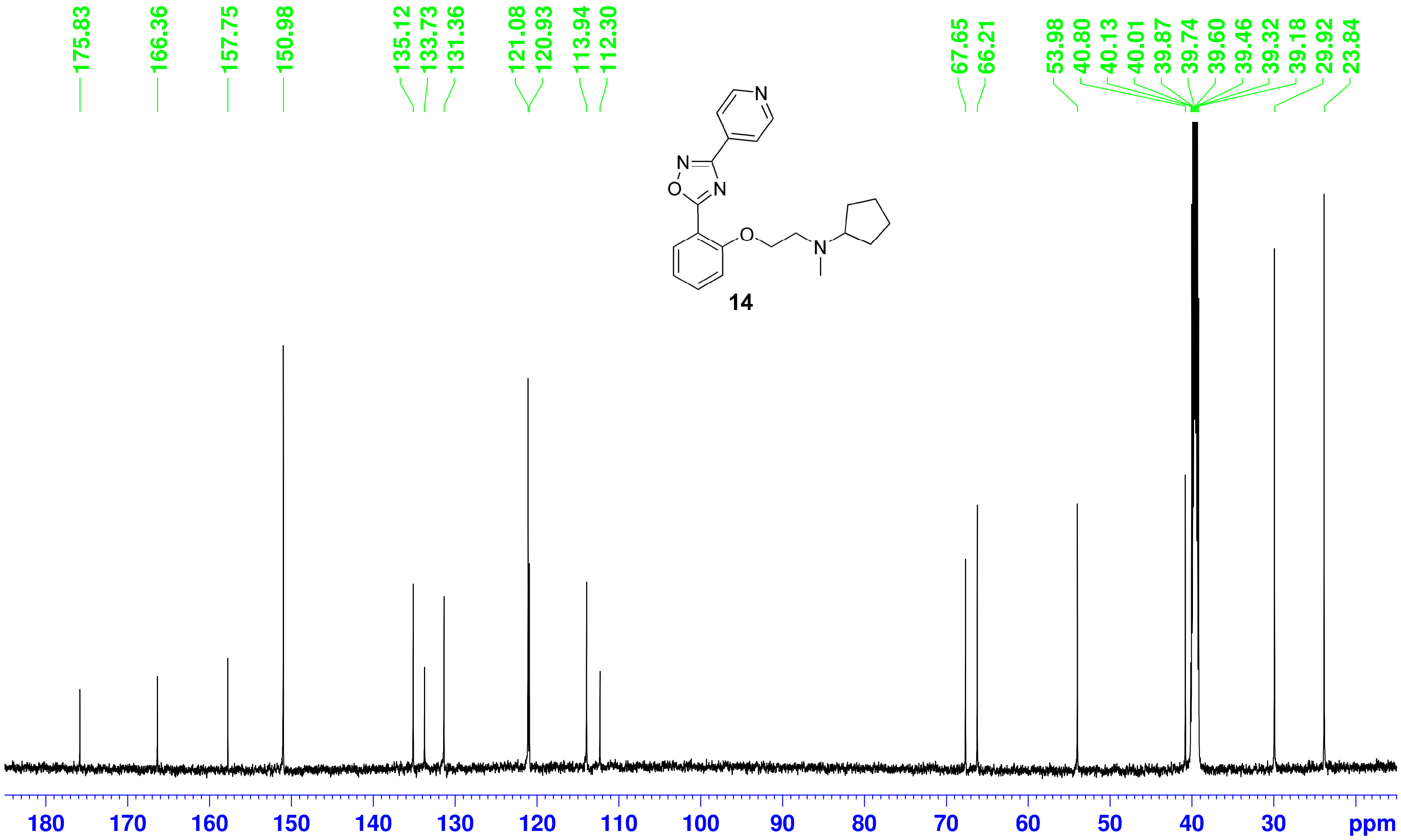

Figure S19. ^{1}H NMR of compound **23** in DMSO-d_6

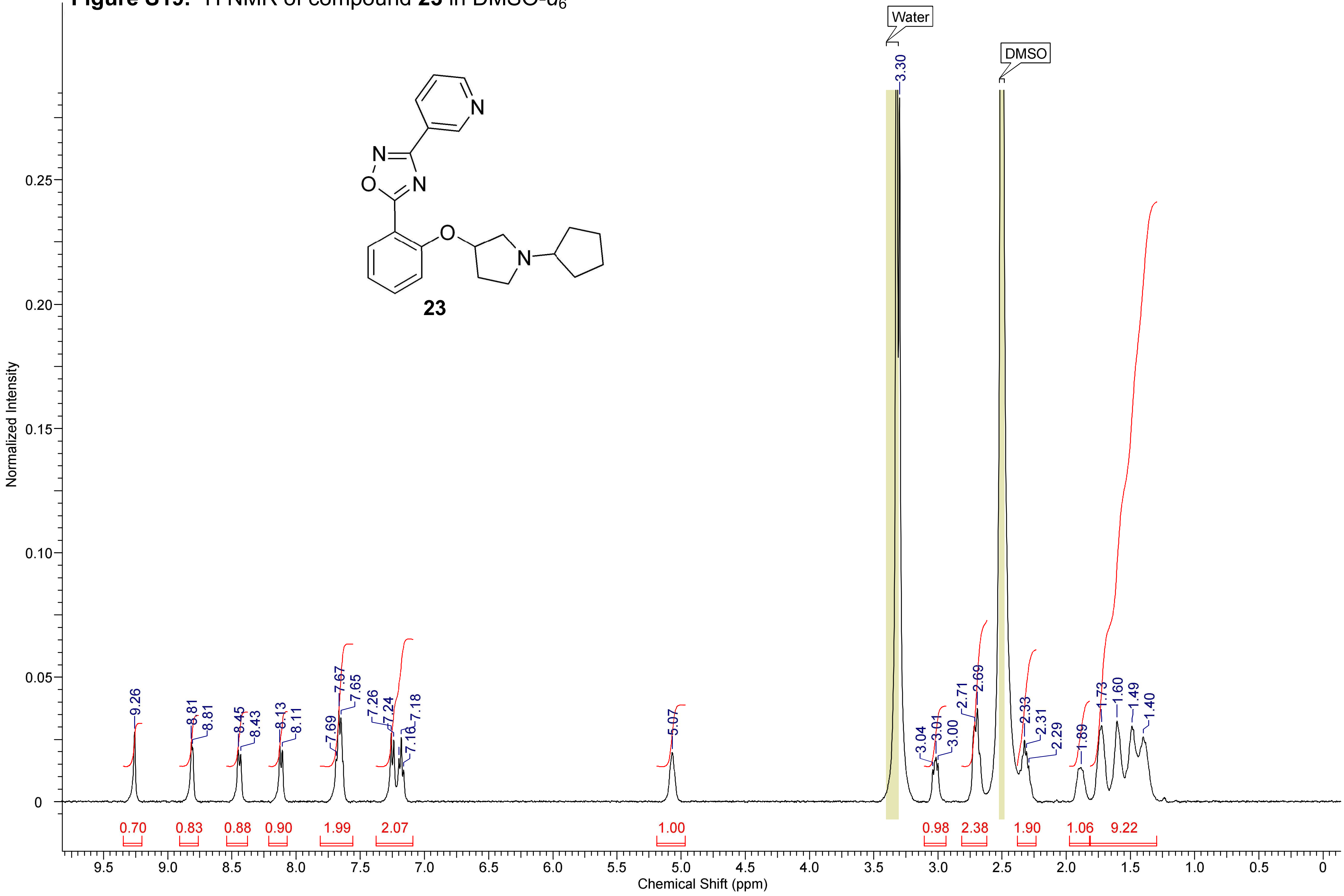

Figure S20. ^{1}H NMR of compound **24** in CD_3OD

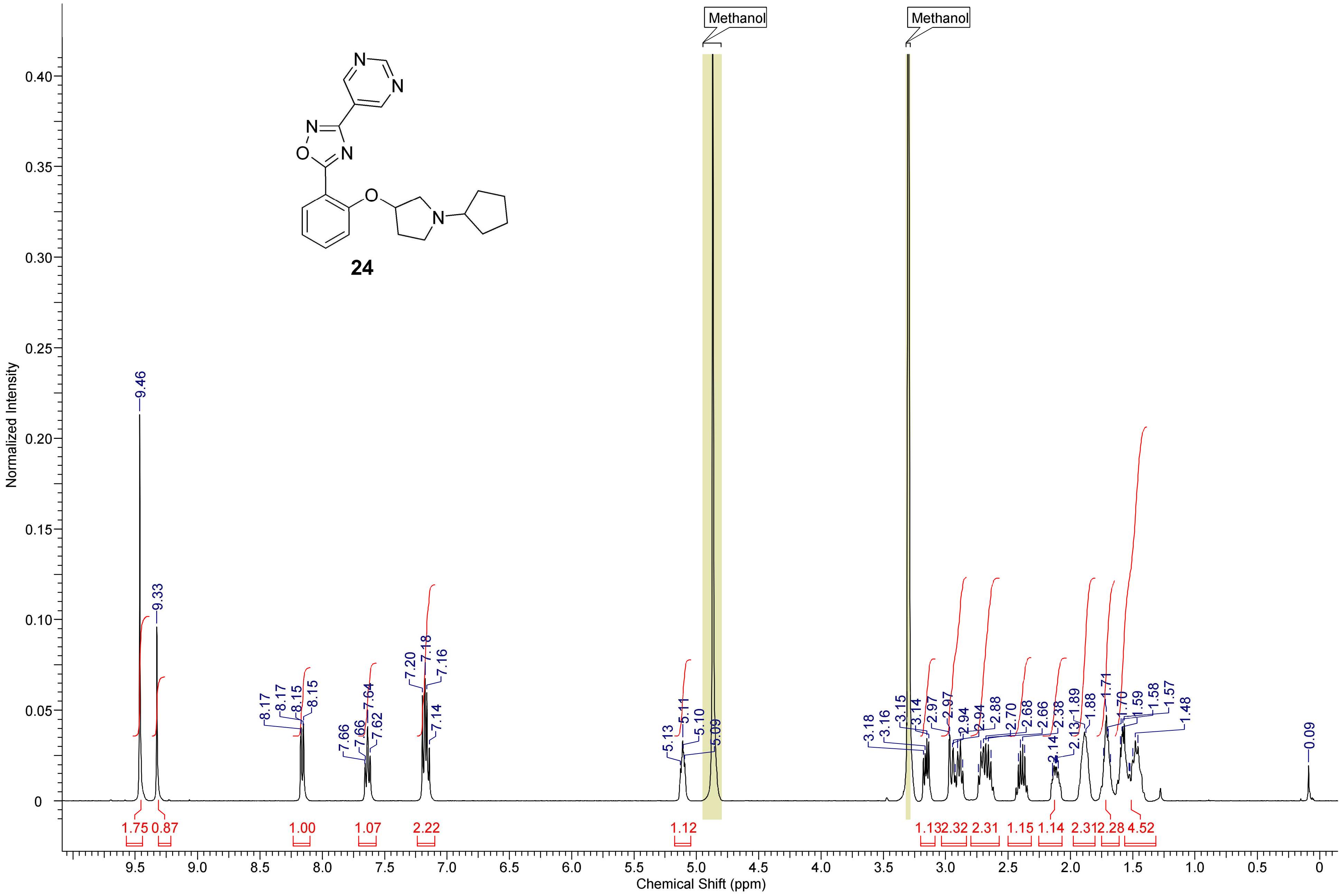

Figure S21. ^{13}C NMR of compound **24** in DMSO-d_6

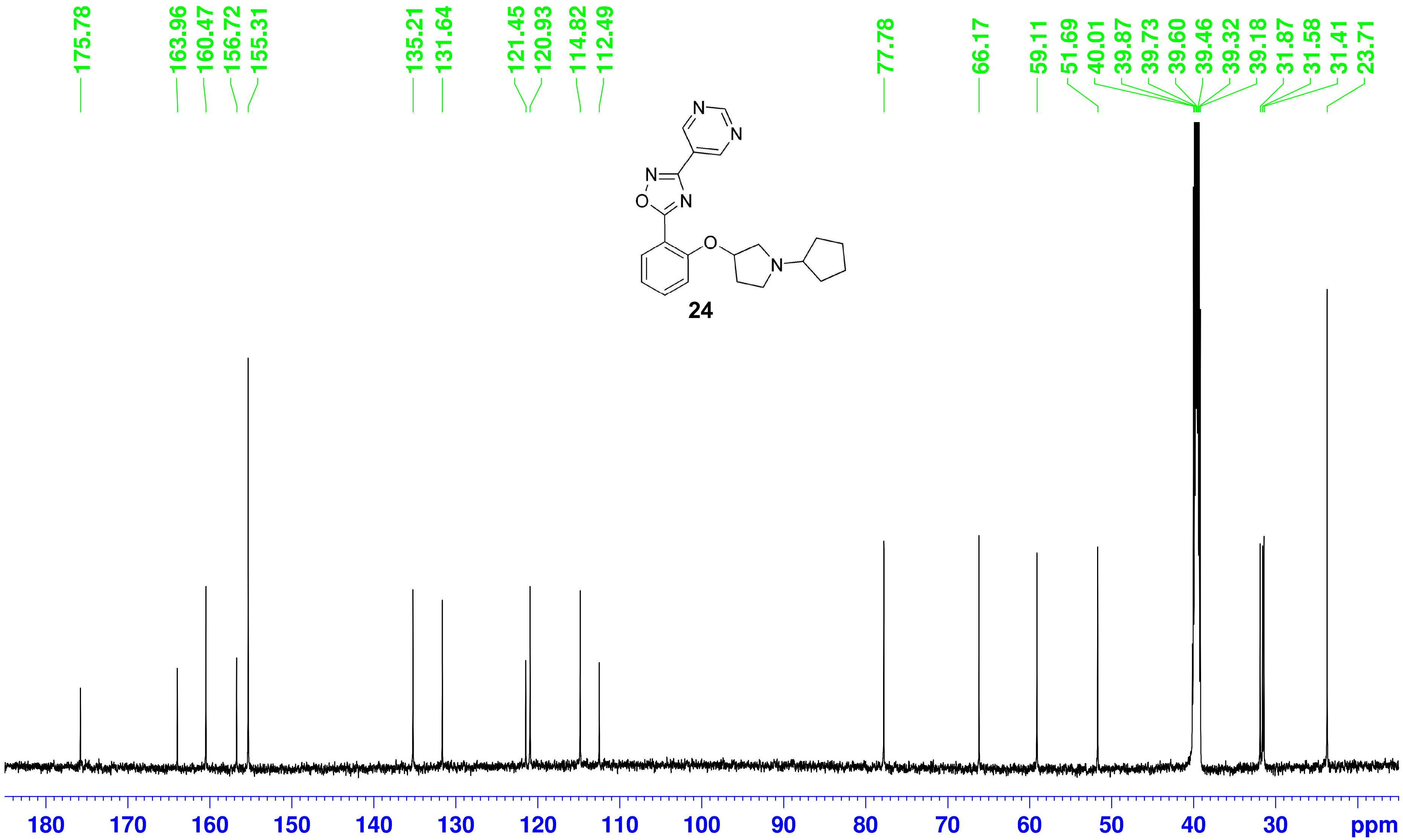

Figure S22. ^{1}H NMR of compound **25** in DMSO-d_6

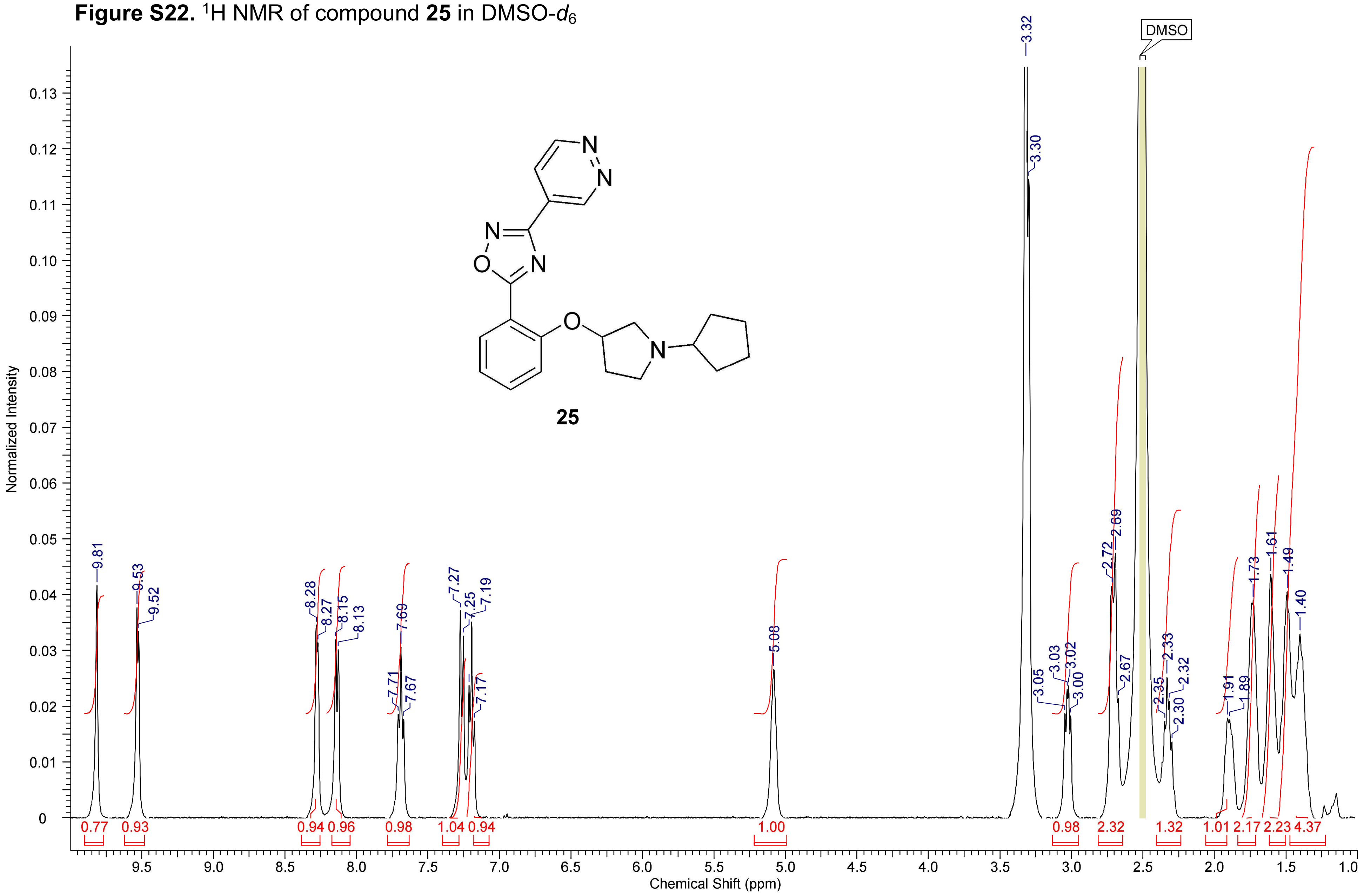

Figure S23. ^{13}C NMR of compound **25** in DMSO-d_6

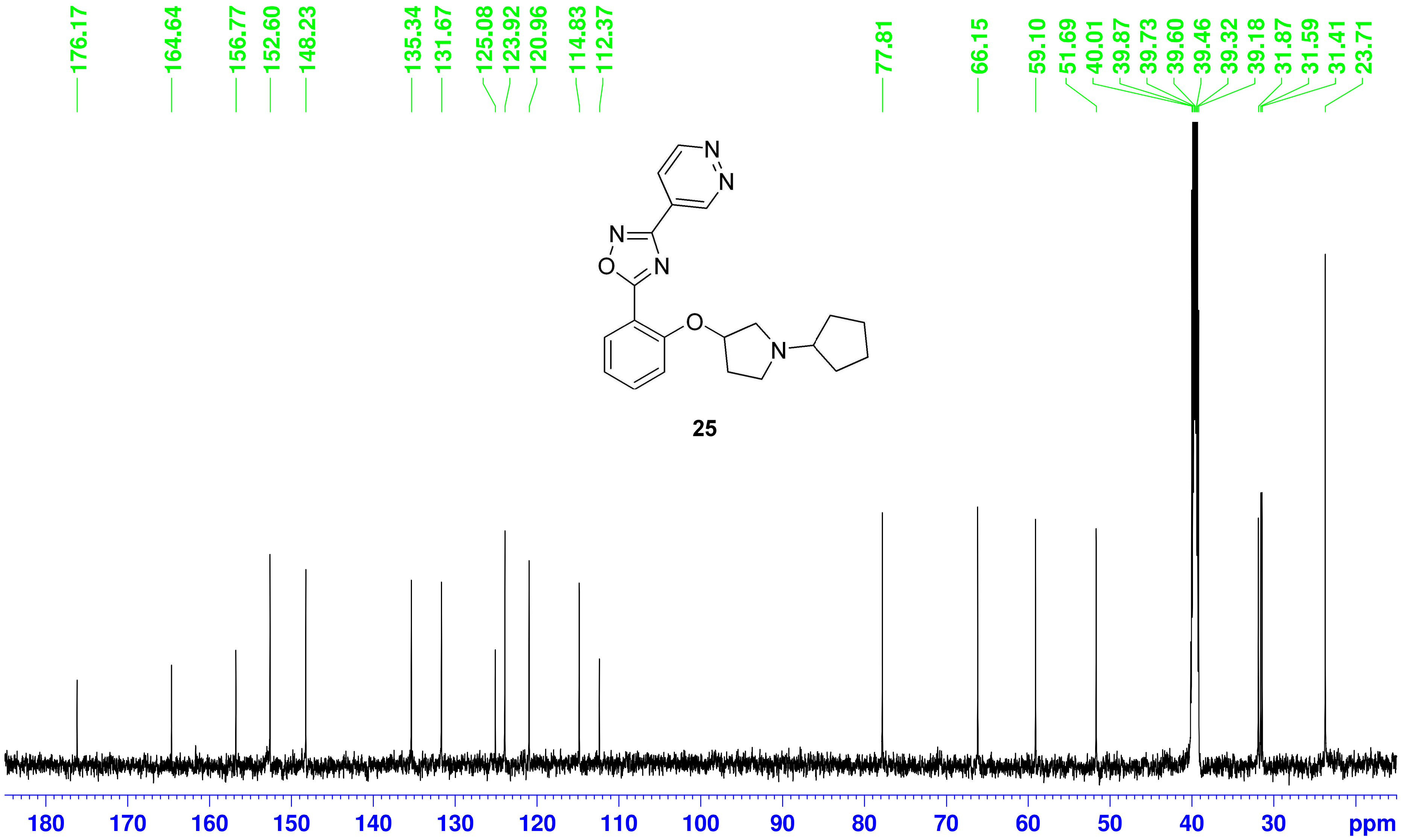

Figure S24. ^{1}H NMR of compound **26** in DMSO-d_6

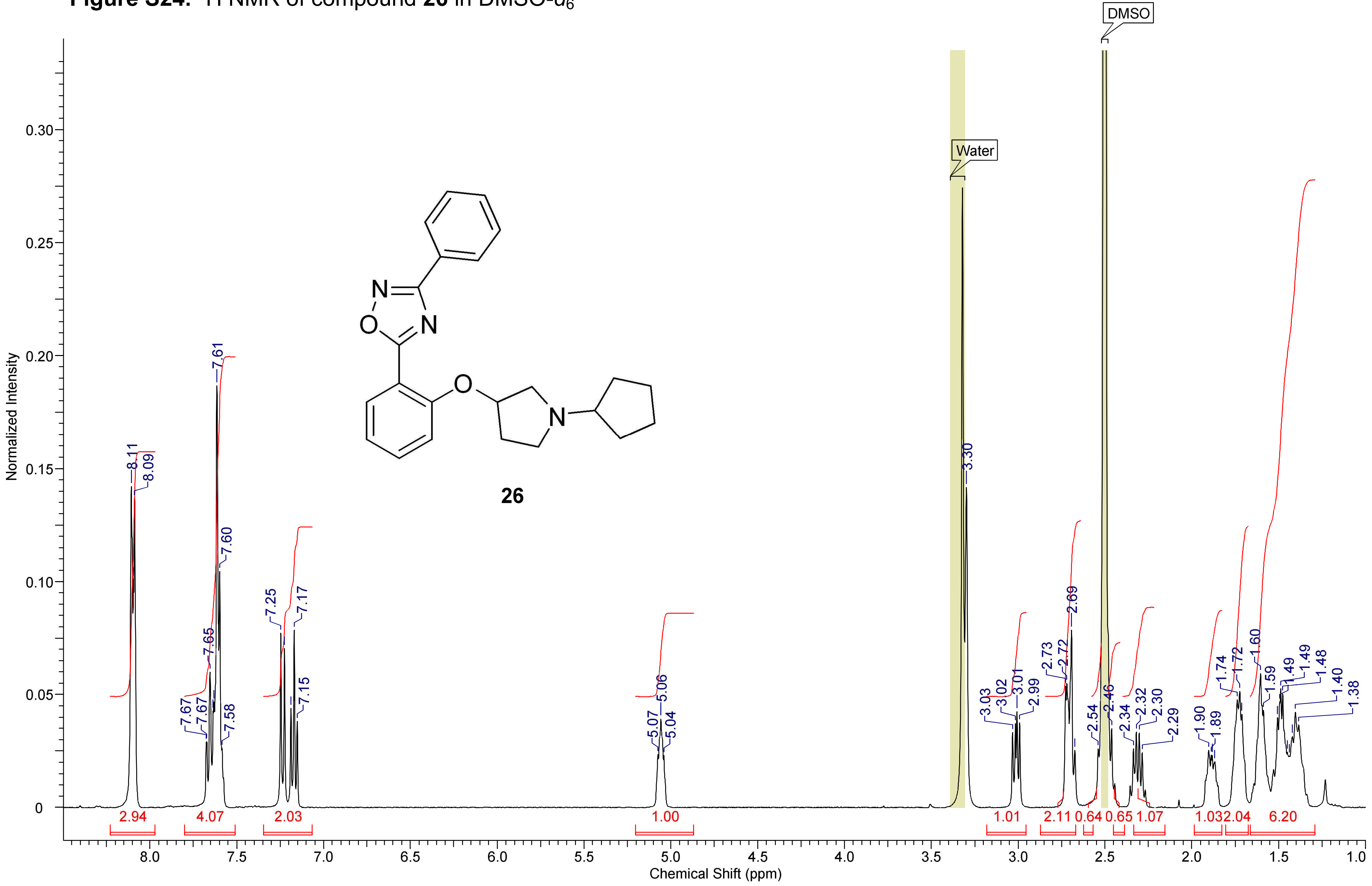

Figure S25. ^{13}C NMR of compound **26** in DMSO-d_6

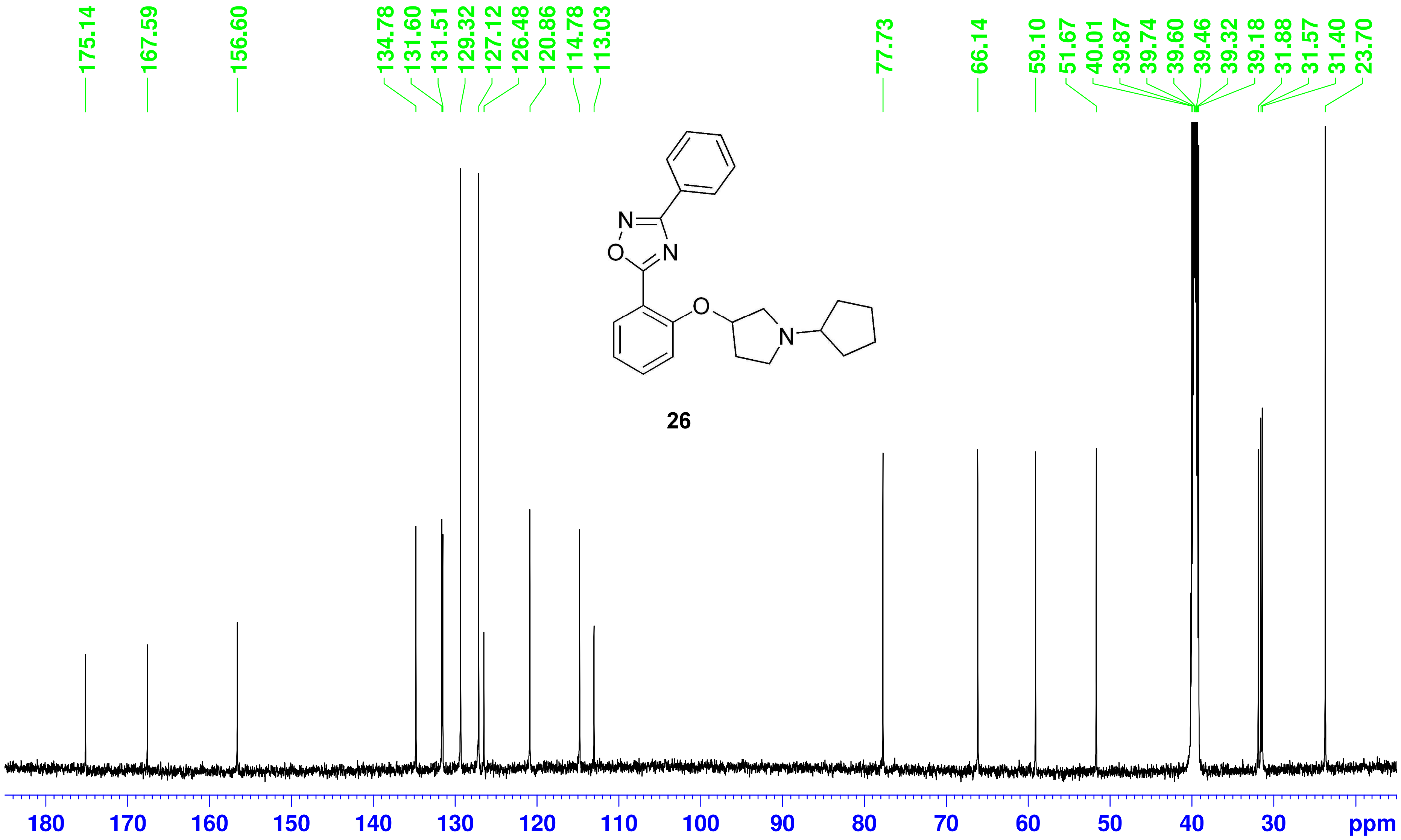

Figure S26. ^{1}H NMR of compound **27** in DMSO-d_6

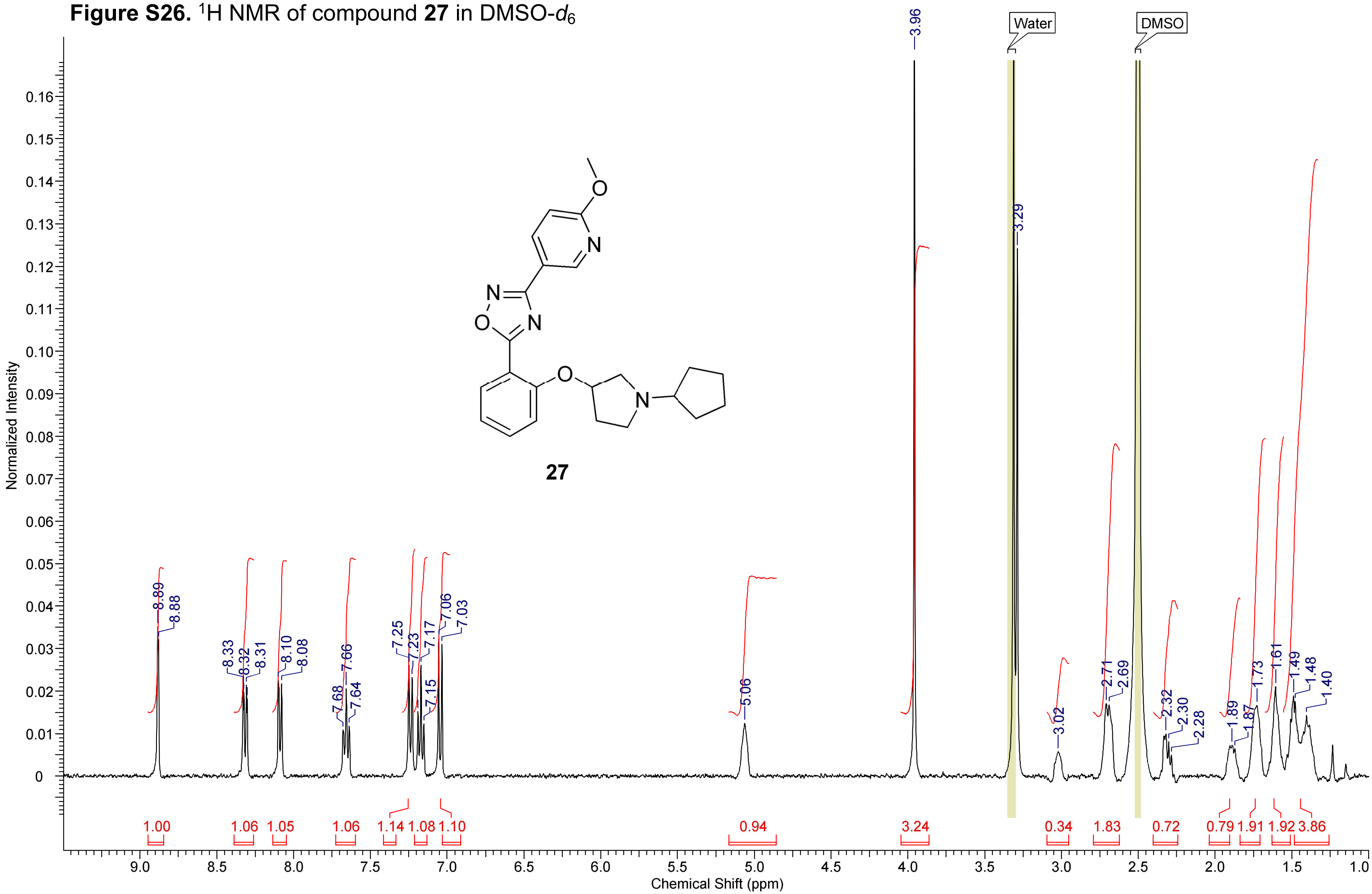

Figure S27. ^{1}H NMR of compound **28** in DMSO-d_6

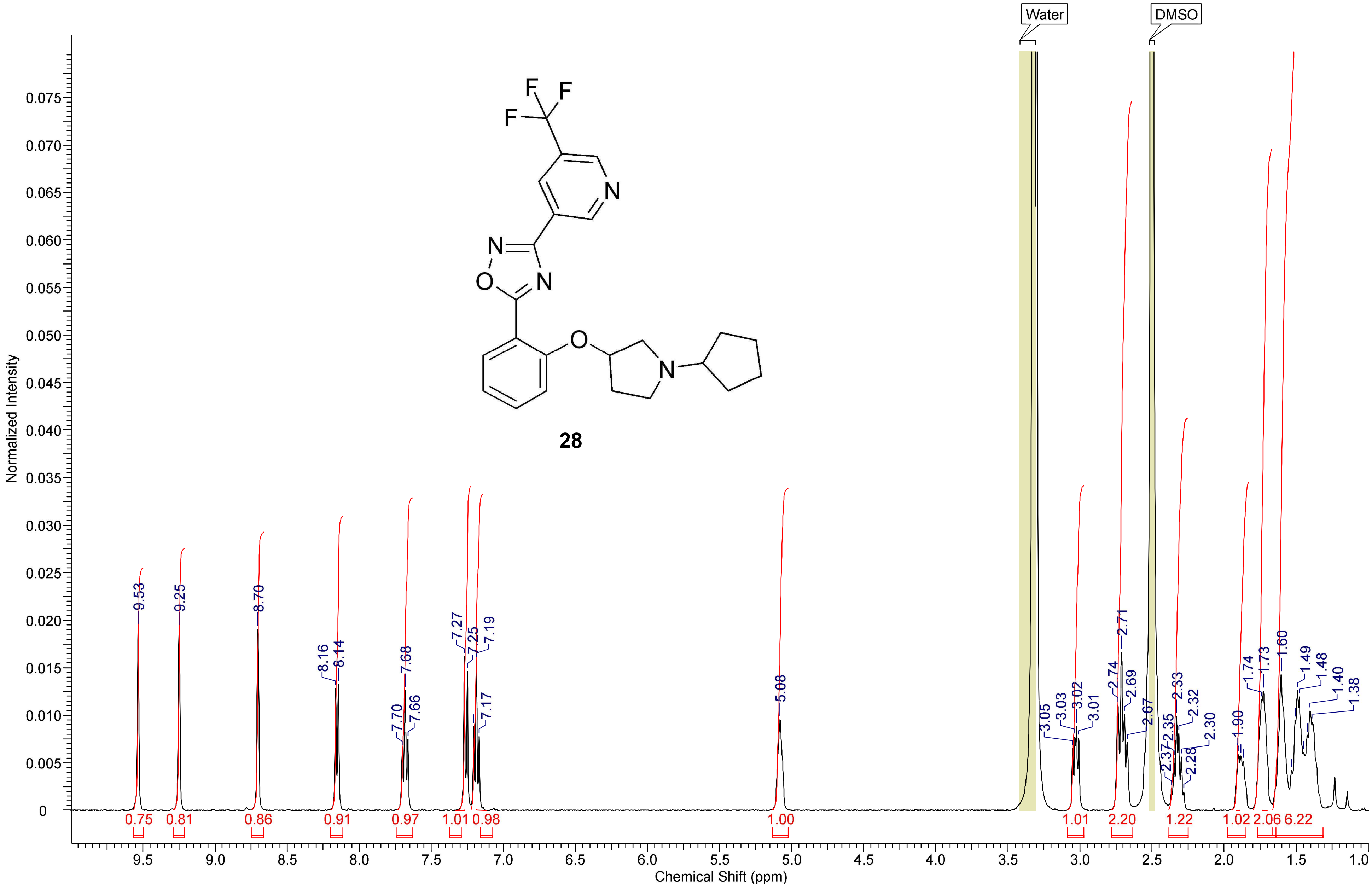

Figure S28. ^{13}C NMR of compound **28** in DMSO-d_6

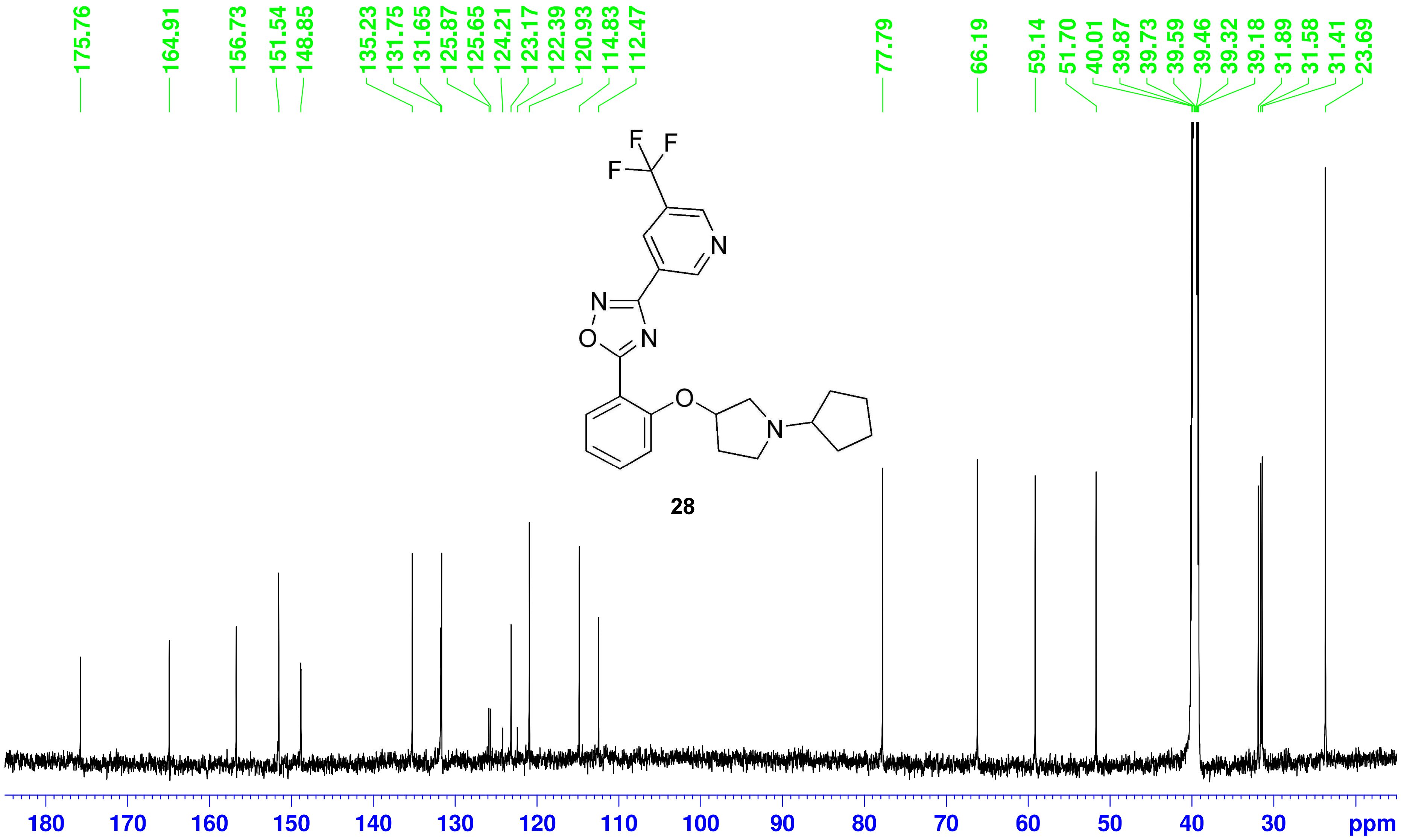

Figure S29. ^{1}H NMR of compound **29** in DMSO-d_6

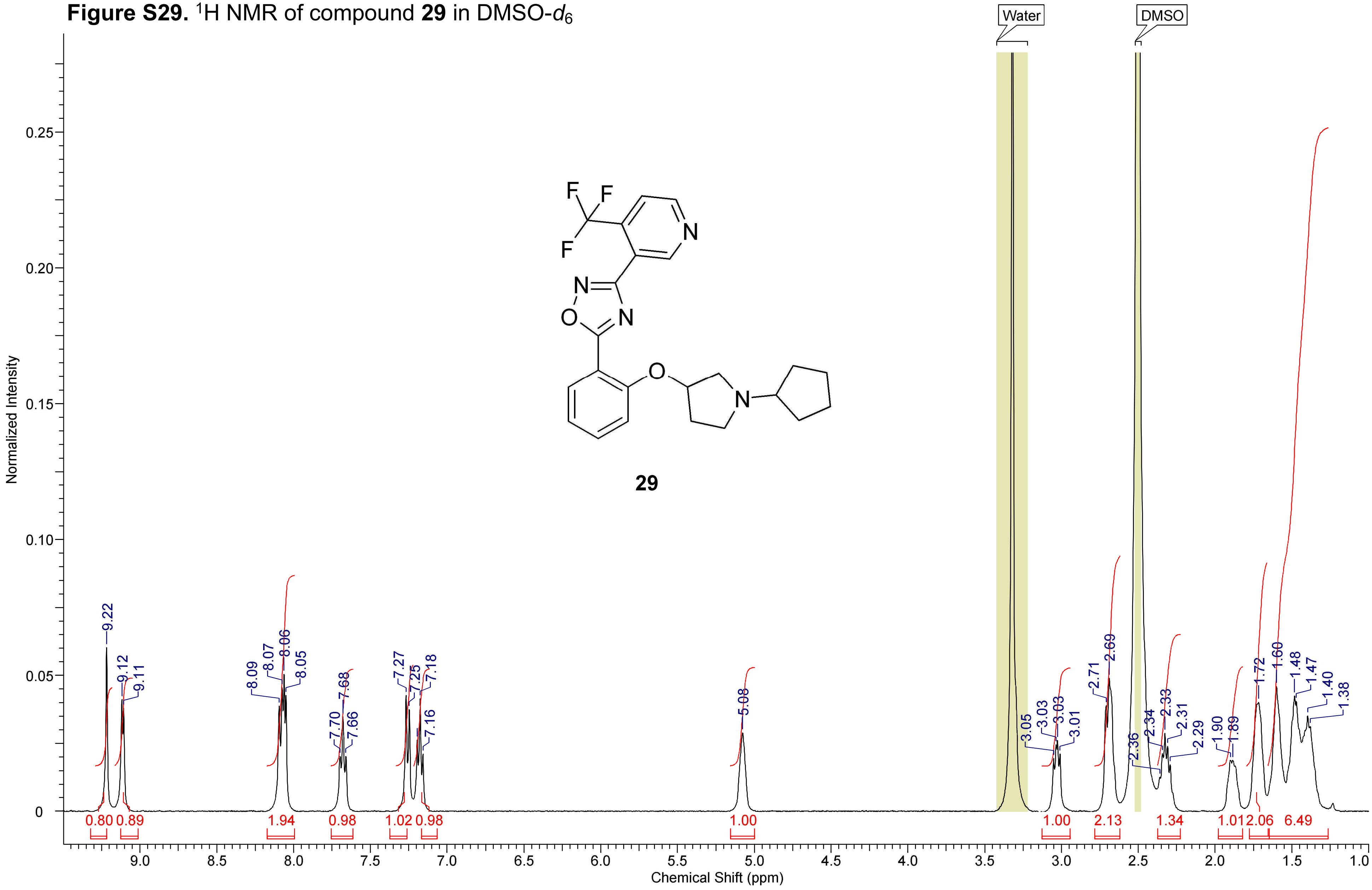

Figure S30. ^{13}C NMR of compound **29** in DMSO-d_6

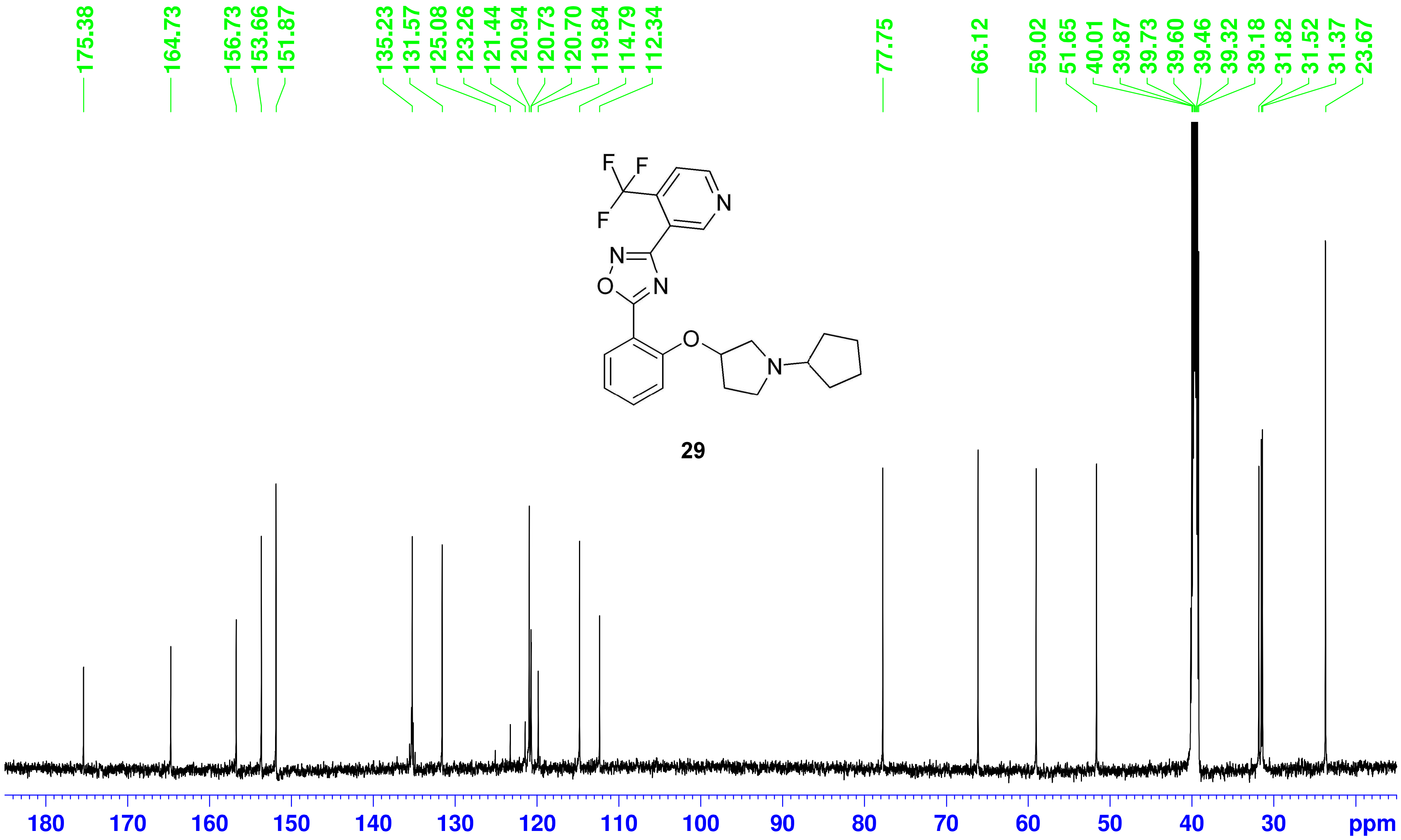

Figure S31. ^{1}H NMR of compound **30** in DMSO-d_6

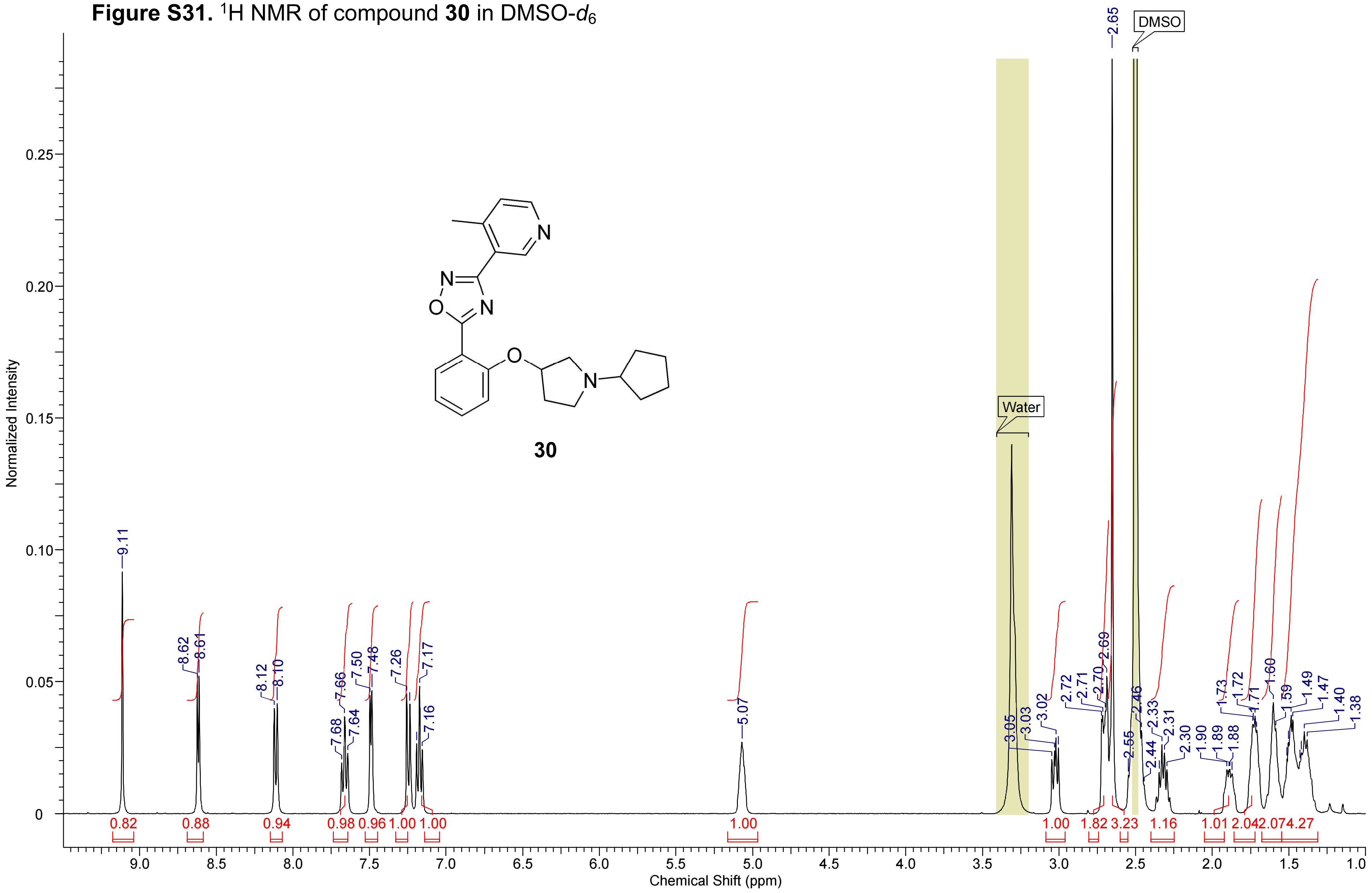

Figure S32. ^{13}C NMR of compound **30** in DMSO-d_6

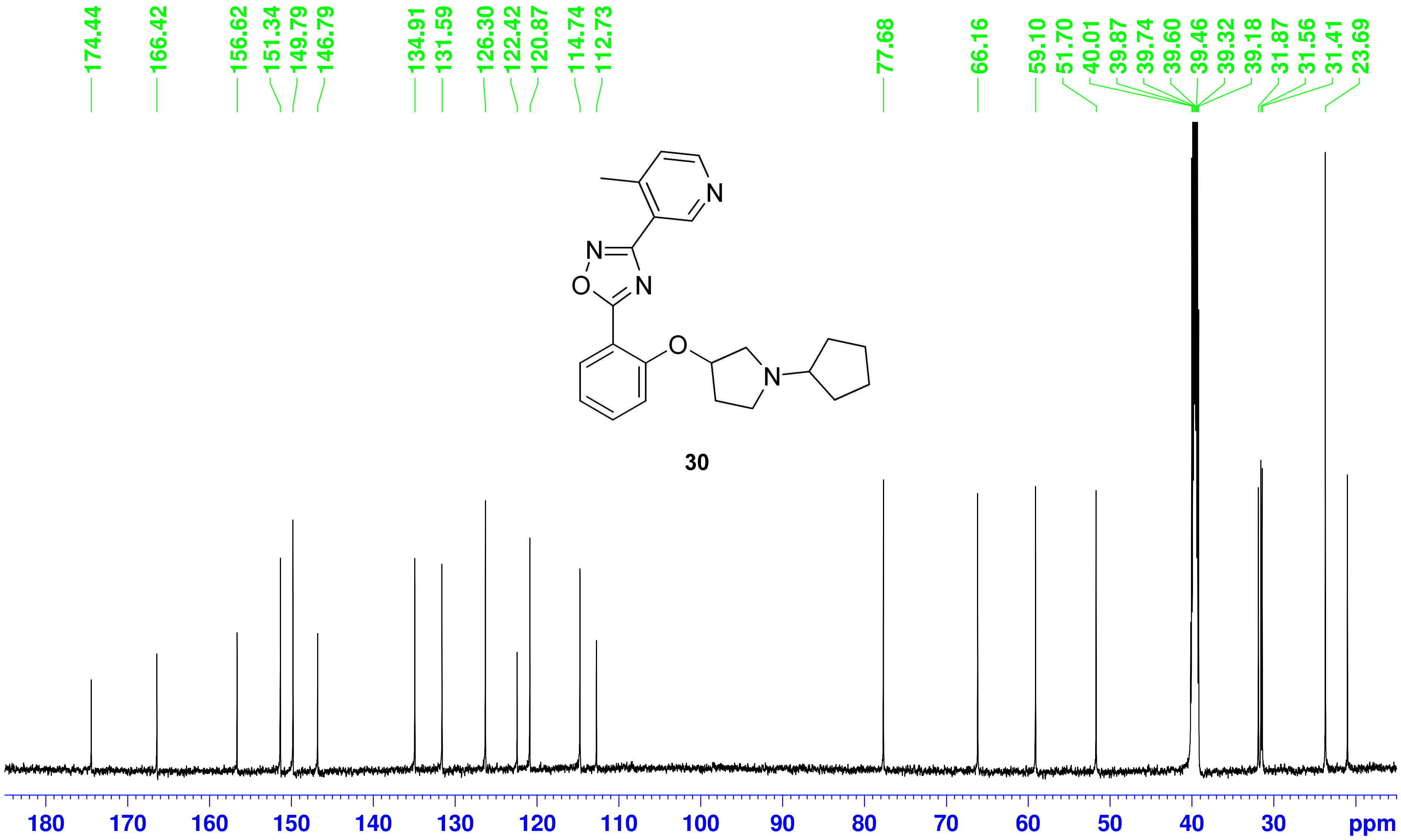

Figure S33. ^{1}H NMR of compound **31** in DMSO-d_6

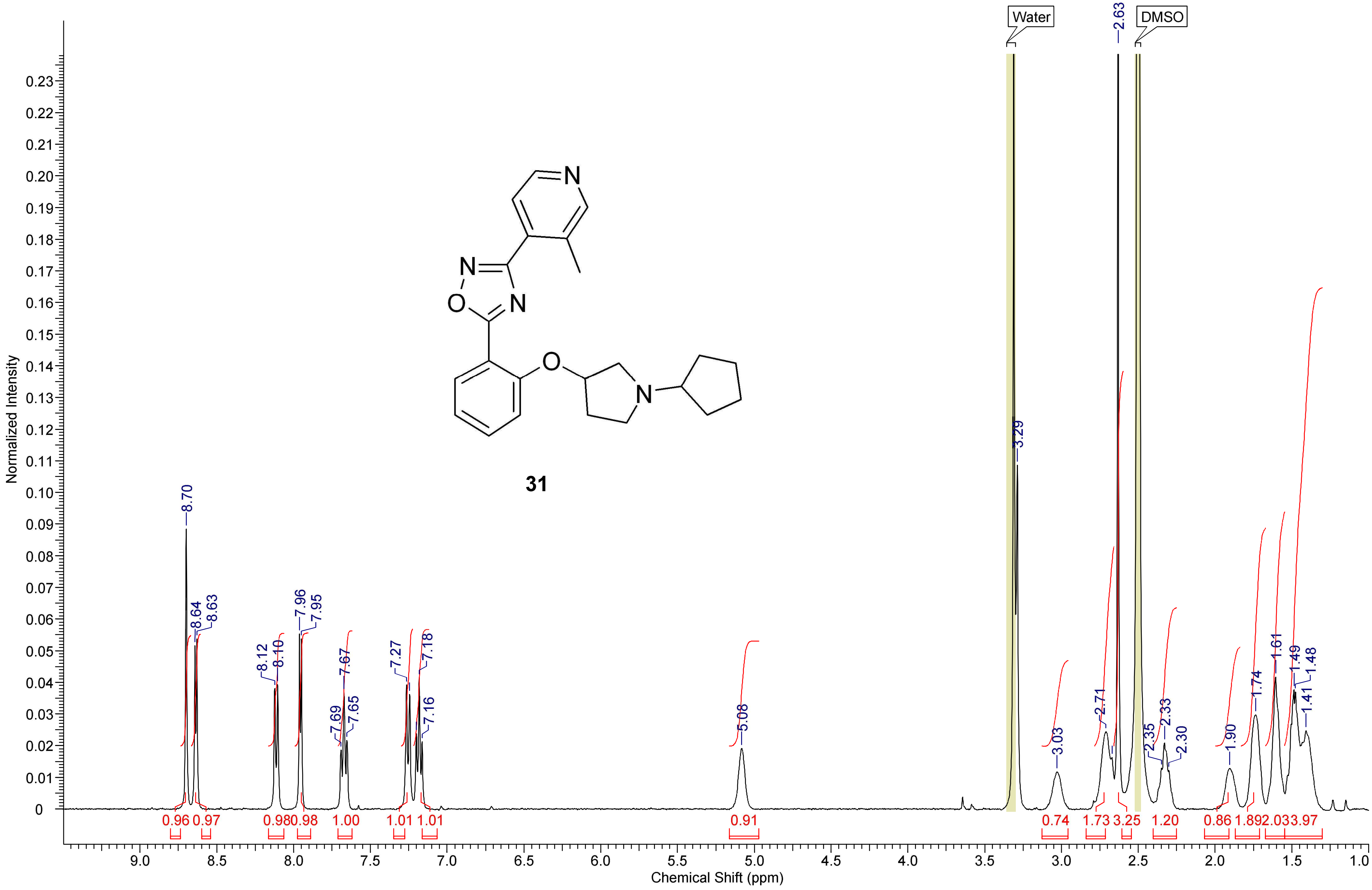

Figure S34. ^{13}C NMR of compound **31** in DMSO-d_6

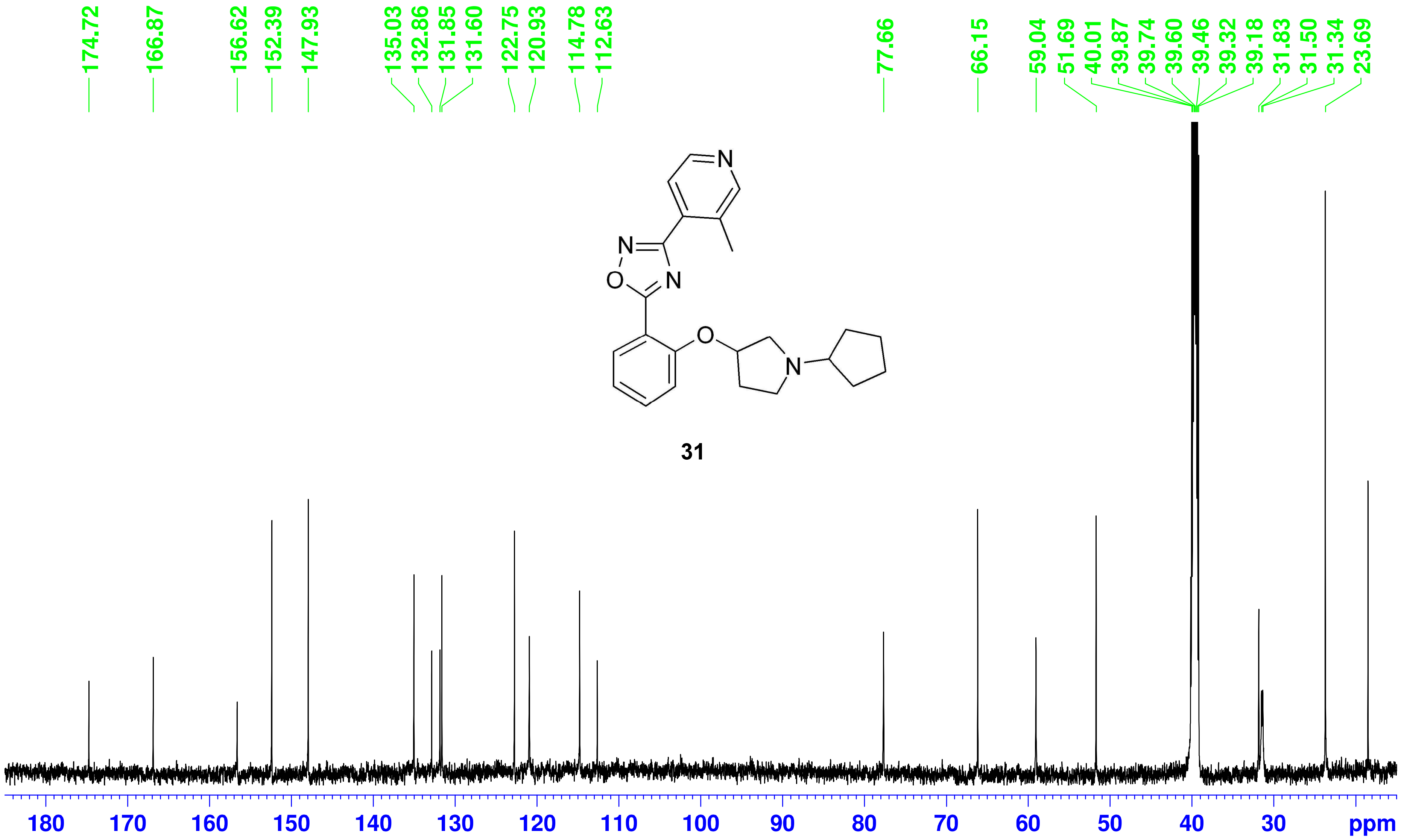

Figure S35. ^1H NMR of compound **35** in DMSO-d_6

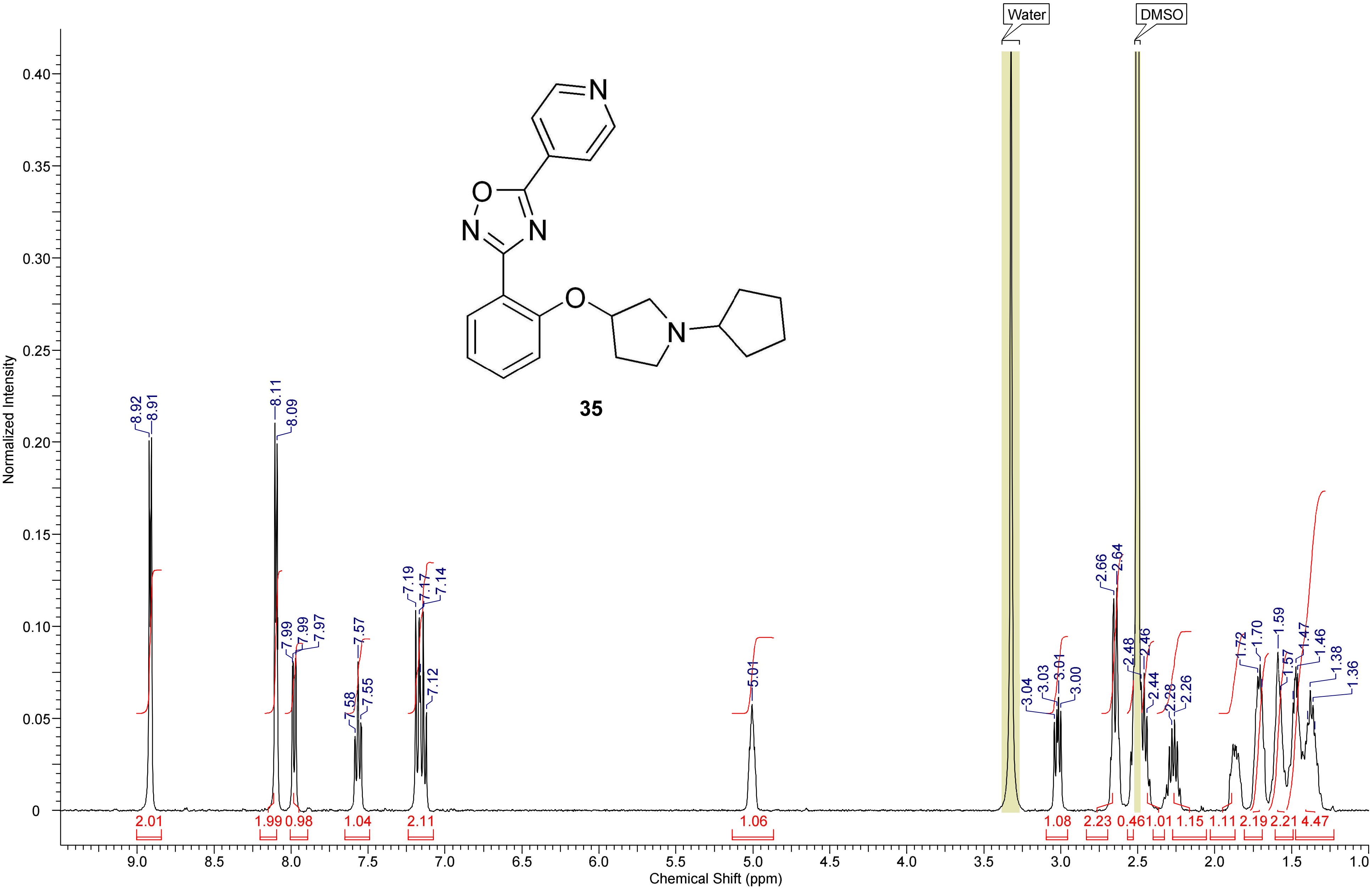

Figure S36. ^{13}C NMR of compound **35** in DMSO-d_6

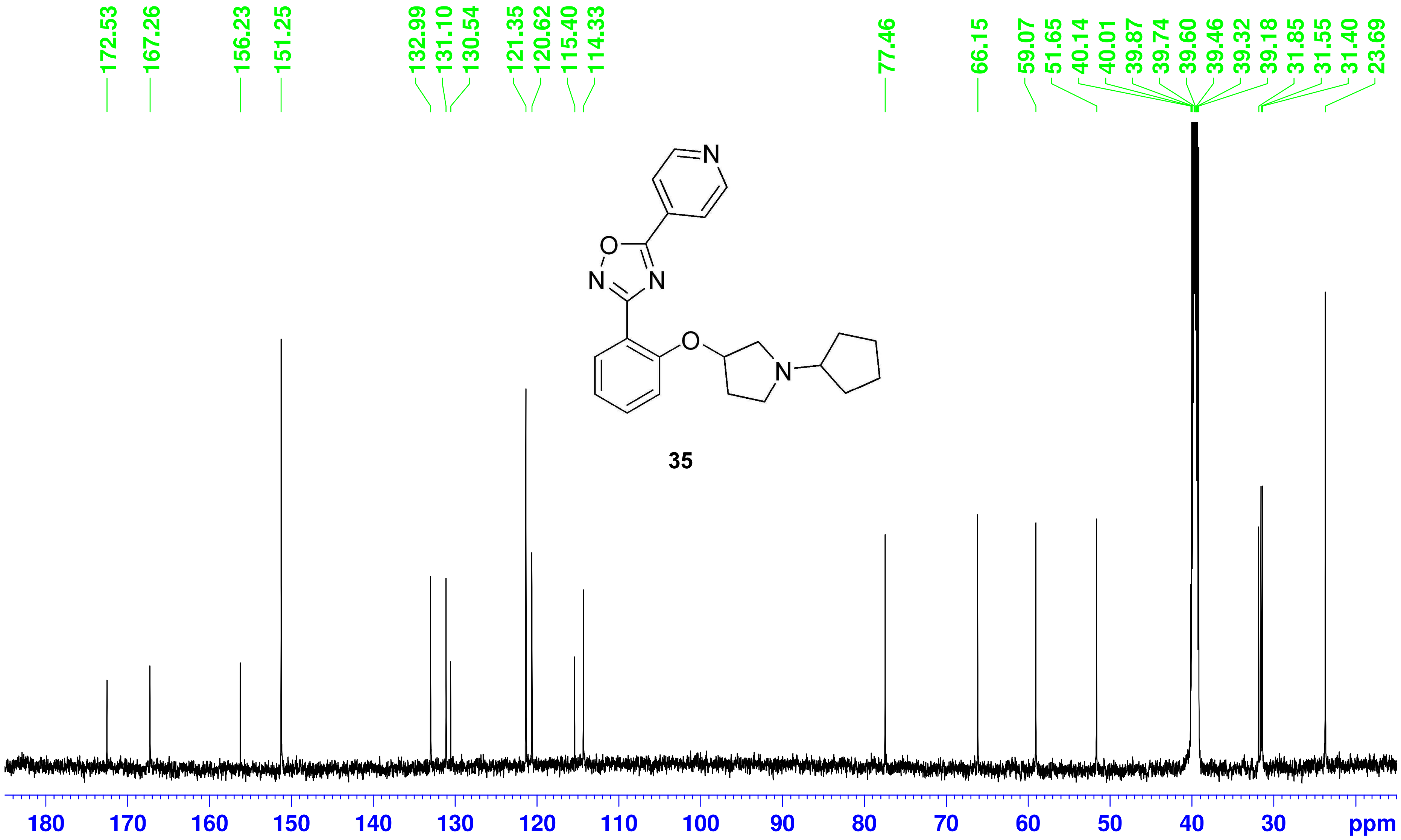

Figure S37. ^{1}H NMR of compound **38** in DMSO-d_6

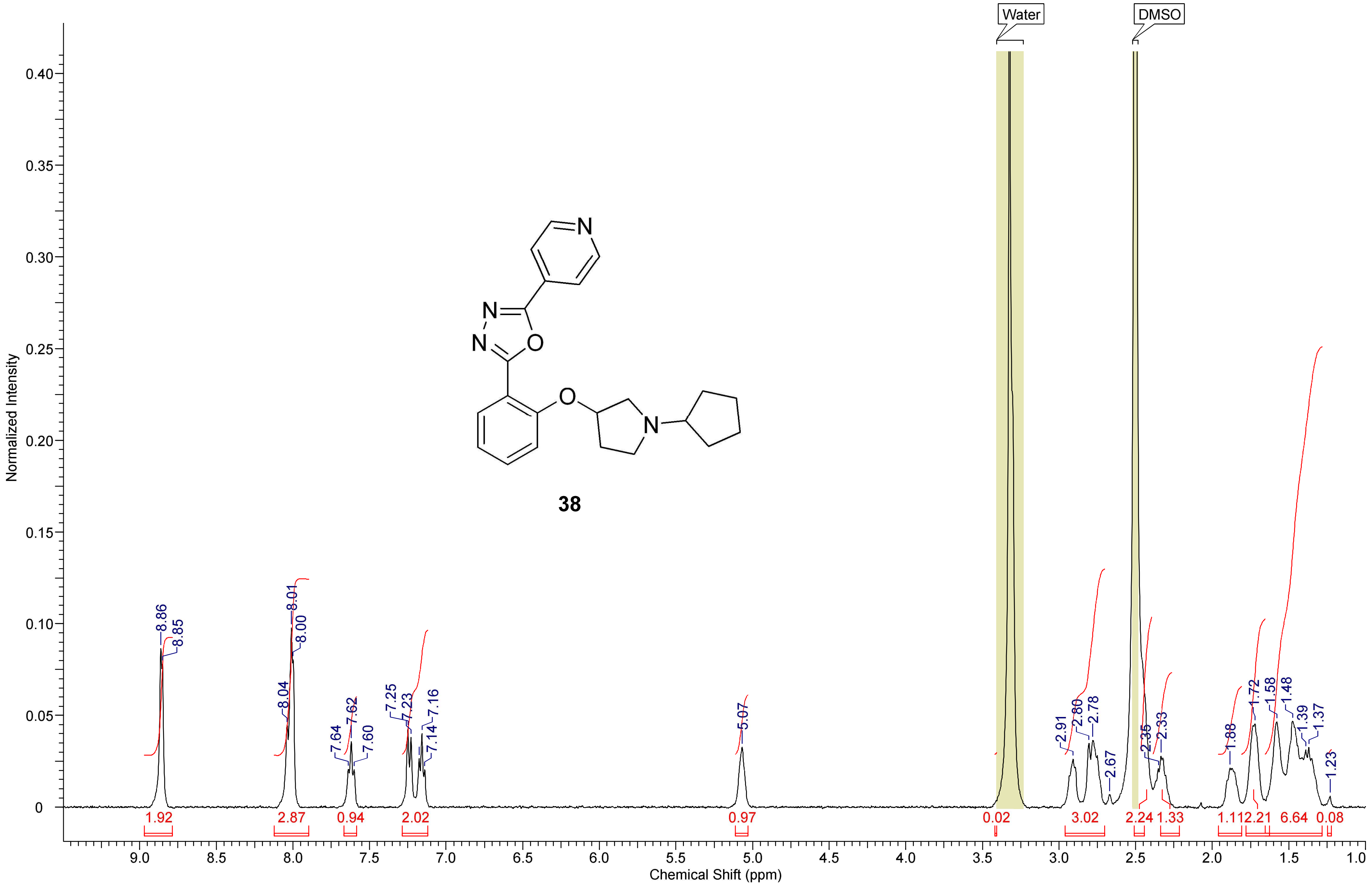

Figure S38. ^{13}C NMR of compound **38** in DMSO-d_6

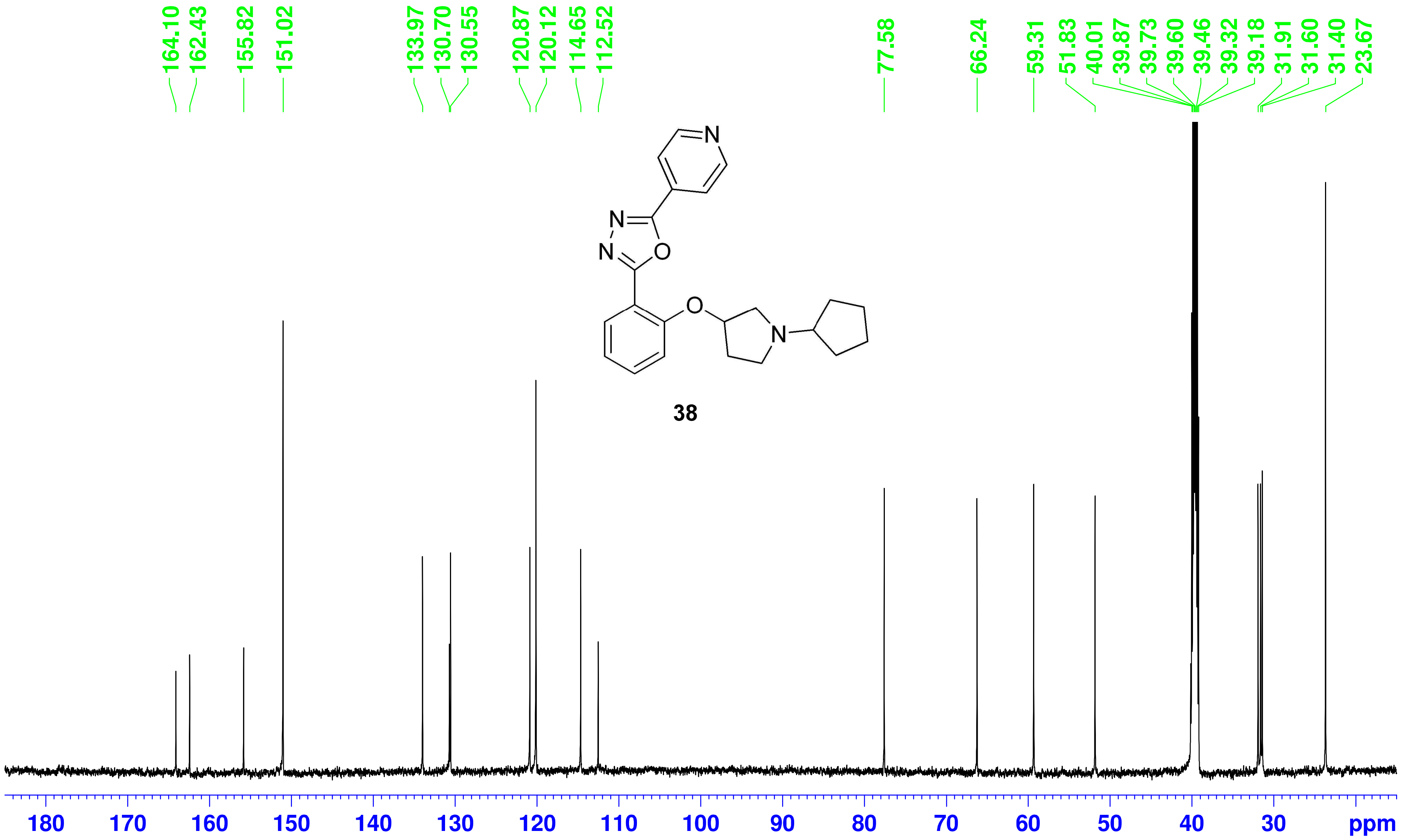

Figure S39. ^{1}H NMR of compound **46** in DMSO-d_6

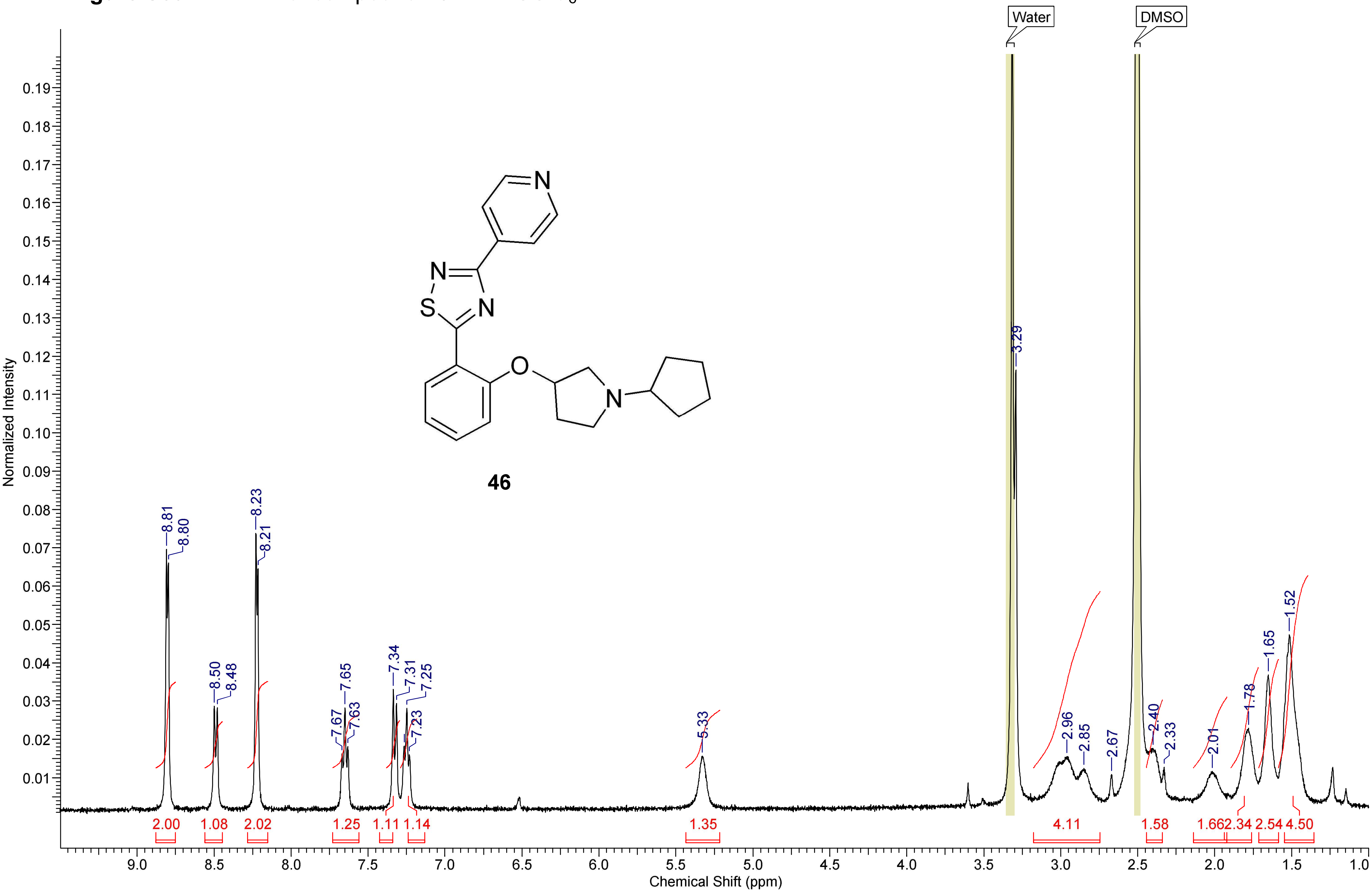

Figure S40. ^{1}H NMR of compound **49** in DMSO-d_6

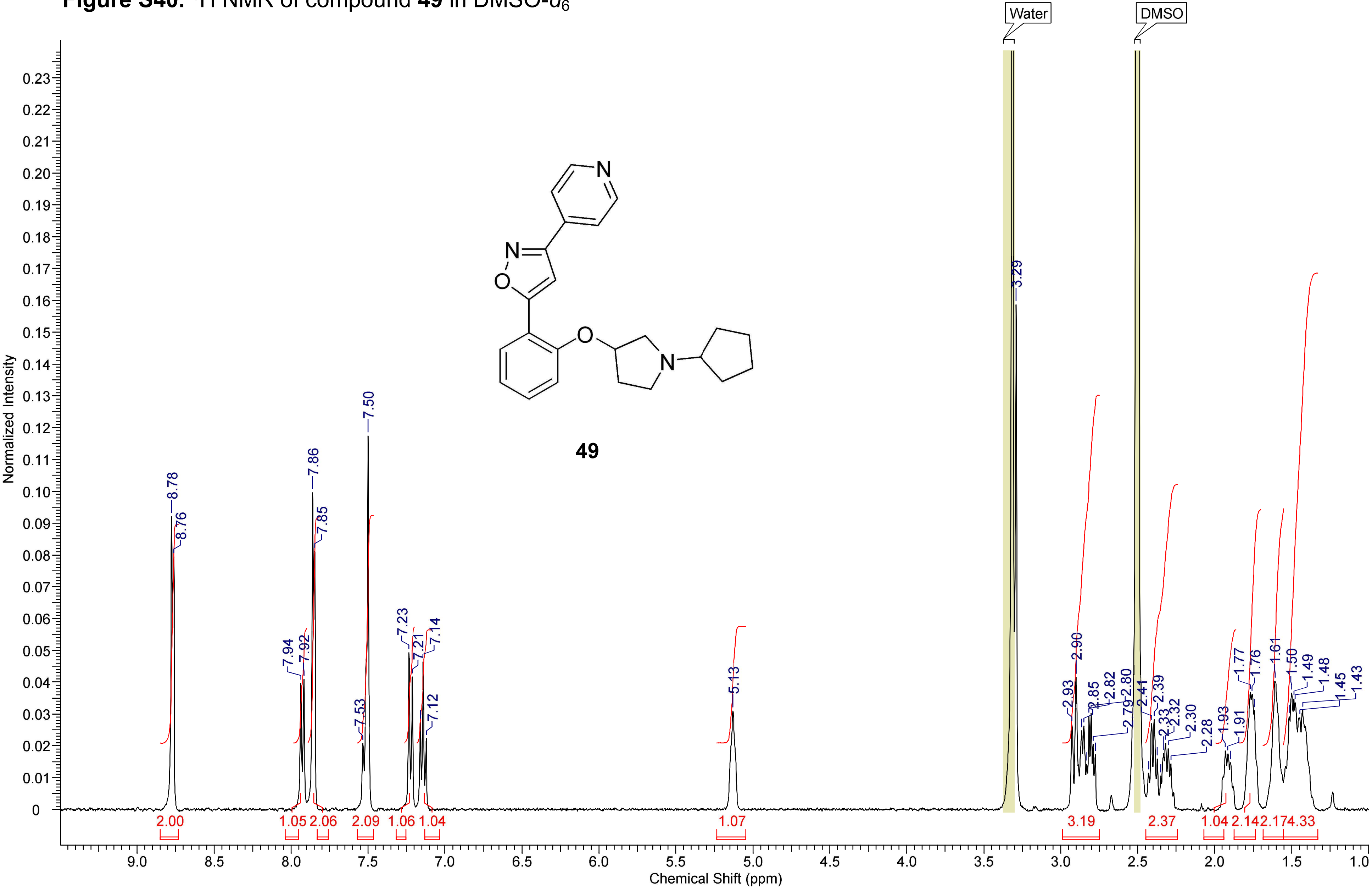

Figure S41. ^{13}C NMR of compound **49** in DMSO-d_6

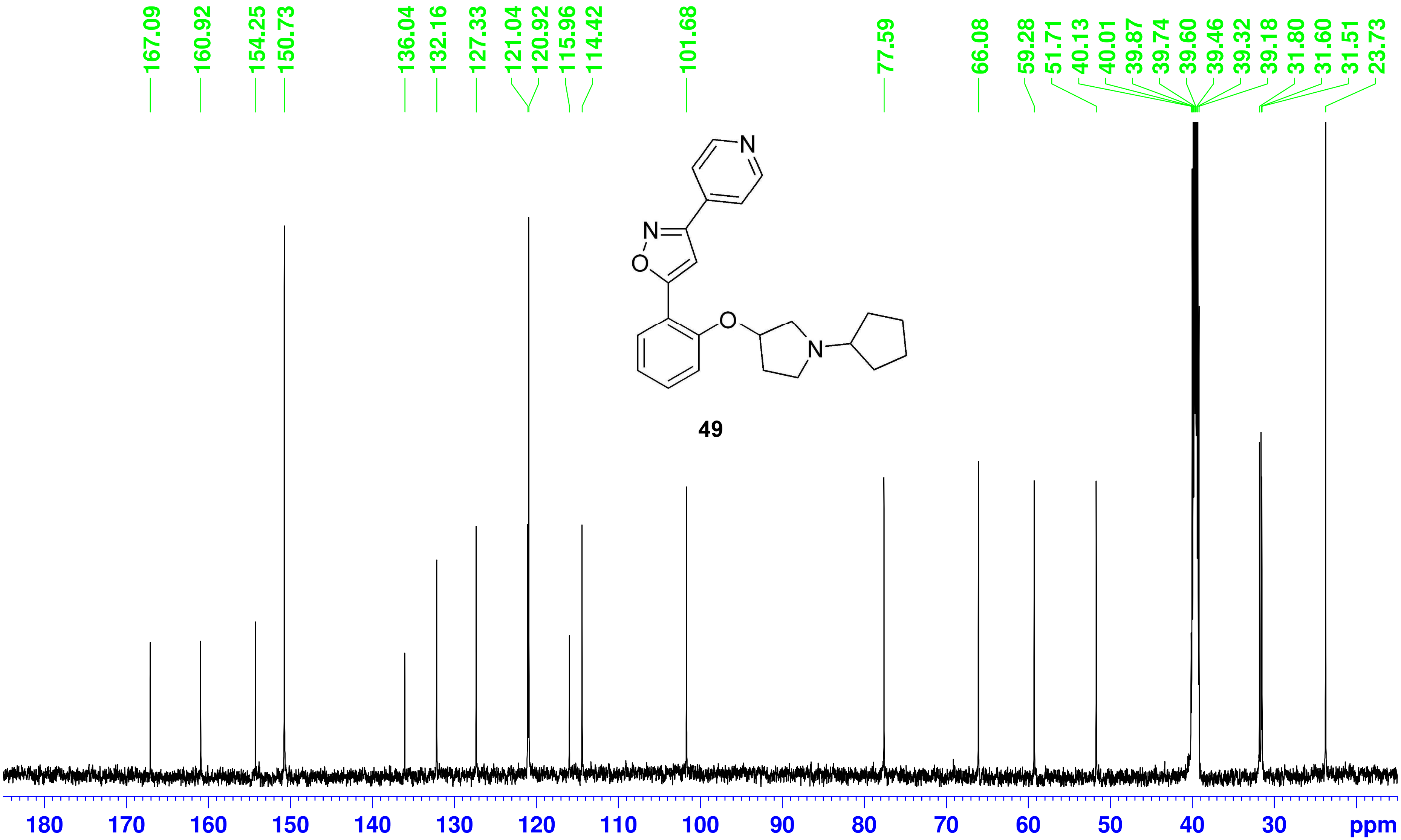

Supplement: Supplementary file 1 [file molecules-27-01052-s001.zip › molecules-1540527-supplementary.pdf]
